# Comprehensive analytical approach identifies a subtype of CTCF+ tumor-associated neutrophils associated with CRC development and as a biomarker for immunotherapy

**DOI:** 10.7150/ijbs.111529

**Published:** 2025-08-16

**Authors:** Jie-pin Li, Yuan-jie Liu, Yi Zhang, Qian-wen Ye, Guo Xu, Jin-chen Chong, Yi Yin, Yang Li, Shuang-shuang Wang, Jin-yong Zhou, Jun Qian, Shen-lin Liu, Xi Zou, Yu-gen Chen

**Affiliations:** 1 Department of Colorectal Surgery, The Affiliated Hospital of Nanjing University of Chinese Medicine, Jiangsu Province Hospital of Chinese Medicine, Nanjing, Jiangsu, 210029, China.; 2 Jiangsu Province Key Laboratory of Tumor Systems Biology and Chinese Medicine, Nanjing, Jiangsu 210029, China.; 3 No. 1 Clinical Medical College, Nanjing University of Chinese Medicine, Nanjing, Jiangsu 210023, China.; 4 Department of Endoscopy Center, Affiliated Hospital of Nanjing University of Chinese Medicine, Jiangsu Province Hospital of Chinese Medicine, Nanjing, Jiangsu 210029, China.; 5 Department of Oncology, Affiliated Hospital of Nanjing University of Chinese Medicine, Jiangsu Province Hospital of Chinese Medicine, Nanjing, Jiangsu 210029, China.; 6 Jiangsu Collaborative Innovation Center of Traditional Chinese Medicine in Prevention and Treatment of Tumor, Nanjing, Jiangsu 210029, China.

**Keywords:** colorectal cancer, tumor-associated neutrophils, CTCF, MIEN1, IL-1β

## Abstract

**Background:** Colorectal cancer (CRC) is an aggressive and heterogeneous tumor with limited therapeutic options. Tumor-associated neutrophils (TANs) play multifaceted roles in the tumor microenvironment (TME) depending on their polarization. Understanding TAN heterogeneity and the mechanisms underlying their tumor-promoting activities is critical for advancing CRC treatment.

**Methods:** This study integrated single-cell RNA sequencing, spatial transcriptomics, bulk RNA sequencing, and in vivo/ex vivo experiments to characterize TANs in CRC.

**Results:** We identified a distinct subpopulation of TANs characterized by high CCCTC-binding factor (CTCF) expression (CTCF+ TANs) enriched in hypoxic tumor regions. CTCF+ TANs exhibit enhanced migratory capacity and IL-1β secretion, correlating with poor prognosis and resistance to immunotherapy. Mechanistically, CTCF regulates the expression of Migration and Invasion Enhancer 1 (MIEN1), which promotes TAN recruitment and migration without triggering inflammation. Functional studies revealed that CTCF+ TANs suppress T-cell immunity, facilitate epithelial-to-mesenchymal transition (EMT), and contribute to CRC progression and metastasis. In vivo, targeting CTCF-MIEN1-IL-1β signaling rescued the immunosuppressive microenvironment and improved the efficacy of anti-PD-L1 therapy.

**Conclusions:** CTCF+ TANs represent a novel TAN subtype that drives CRC progression and immunosuppression via the CTCF-MIEN1-IL-1β axis. These findings highlight the potential of targeting CTCF+ TANs to overcome immunotherapy resistance and improve patient outcomes.

## Introduction

Colorectal cancer (CRC) ranks among the top three most prevalent cancers worldwide, accounting for approximately 10% of all cancer cases 1. Its incidence varies geographically, with higher rates in North America and Western Europe compared to Asia, Africa, and Latin America 1. This disparity is attributed to differences in lifestyle and dietary habits, particularly the consumption of high-fat and high-protein diets, which are associated with increased CRC risk 2, 3.

Recent advancements in immunotherapy have shown promising results in the treatment of CRC, especially in metastatic settings 4. Studies evaluating the combination of PD-1/PD-L1 inhibitors with standard chemotherapy or anti-angiogenic agents have demonstrated significant improvements in progression-free and overall survival among advanced CRC patients 5-7. These innovative approaches provide new and promising therapeutic options for this challenging disease.

Tumor-associated neutrophils (TANs) have emerged as significant players in the tumor microenvironment (TME) of CRC, where they exhibit a dual role in cancer progression. Depending on their polarization state, TANs can adopt either an antitumorigenic N1-like phenotype or a protumorigenic N2-like phenotype 8, 9. In CRC, TANs have been shown to influence tumor growth, metastasis, and immune evasion through mechanisms such as the release of cytokines, modulation of T-cell activity, and promotion of angiogenesis 10-12. Notably, the presence of TANs in CRC has been associated with poor prognosis, underscoring their potential as therapeutic targets 13, 14. The mechanisms underlying TAN polarization and function in CRC remain incompletely understood, warranting further investigation.

CCCTC-binding factor (CTCF), a transcription regulator known for its role in chromatin organization and gene expression 15, has recently been implicated in immune cell function 16. In neutrophils, CTCF may influence gene expression programs that dictate their migratory behavior and inflammatory responses. Given the critical role of TANs in CRC and the emerging evidence of CTCF's involvement in immune regulation, investigating CTCF in TANs could provide novel insights into CRC progression and therapy.

Here, we identified a subpopulation of TANs with high CTCF signaling activity in CRC by analyzing single-cell RNA sequencing (scRNA-seq) data. Combined with spatial transcriptomic and bulk RNA-seq data, we found that the abundance of CTCF+ TANs was enriched in CRC tissues and negatively correlated with patient prognosis. Through in vivo and ex vivo experiments, we demonstrated that CTCF promotes the transcription of Migration and Invasion Enhancer 1 (MIEN1), enhancing neutrophil migration toward cancer cells, and stimulates IL-1β secretion, leading to a malignant phenotype in CRC cells. Additionally, CTCF+ TANs contribute to an immunosuppressive microenvironment in CRC, potentially reducing the efficacy of immunotherapy. In conclusion, our study identified a class of TANs associated with CRC development, providing new insights into potential therapeutic targets. Thus, CTCF+ TANs represent a promising therapeutic target for CRC.

## Materials and Methods

A comprehensive inventory of all chemical compounds and antibodies used in this study is provided in the Supplementary Material (Table S1). Antibody concentrations were optimized based on the manufacturers' recommendations or previously published research findings. The Supplementary Material also include detailed information on data analysis methods, as well as additional supporting tables, figures, and experimental details to ensure transparency and reproducibility of the study.

### Public data source

Bulk Sequencing Data: The bulk data covered in this study are built-in to the Biomarkers Exploration and Systematic Tool (BEST) database 17. ScRNA-seq Data: The scRNA-seq dataset GSE178318 18, which focuses on CRC, was obtained from the Tumor Immune Single-cell Hub (TISCH) database 19. GSE178318 was primarily utilized to investigate the source of the neutrophil population that we are concerned with.

Spatial transcriptome (ST) data: ST data were retrieved and downloaded from the Comprehensive Repository of Spatial Transcriptomics (CROST) portal 20 and the following keywords were used as a search strategy: colorectal cancer, colon cancer, and rectal cancer. A total of 8 primary CRC samples and 4 CRC liver metastasis (CRLM) samples were included in this study.

### Preparation of Single Cell Suspensions from Surgical Specimen

We performed scRNA-seq to analyze molecular features and cell populations during CRC progression. Tissue samples, including normal epithelium, adenoma, and adenocarcinoma, were collected from the same patient during endoscopic surgery. Clinicopathological details are provided in Figure S1. Nine samples were obtained from three patients who had not received preoperative chemotherapy or radiotherapy. The study was approved by the Jiangsu Province Hospital of Chinese Medicine Ethics Committee (2021NL-206-01), and informed consent was obtained. During endoscopic resection, tissues were confirmed via intraoperative pathology and immediately transferred to the laboratory in ice-cold Phosphate Buffered Saline (PBS) (calcium- and magnesium-free) containing 0.04% Bovine serum albumin (BSA) to maintain cell viability. Specimens were minced into 1-2 mm³ fragments on ice and digested in PBS containing collagenase III (0.5 mg/mL), trypsin (0.25%), and DNase I (10 µg/mL) at 37°C for 30 minutes with gentle shaking (800 rpm), followed by an additional 1-hour incubation.

To preserve RNA integrity and minimize neutrophil degradation, the entire process from tissue collection to Gel-Bead in Emulsions (GEMs) generation and Polymerase chain reaction (PCR) initiation was completed within 2 hours. The resulting suspension was diluted with 4 mL Dulbecco's Modified Eagle Medium (DMEM), filtered through a 40-µm strainer, and centrifuged at 250 × g for 5 minutes at 4°C. Cells were washed twice with cold PBS, treated with red blood cell lysis buffer at 4°C for 10 minutes, and centrifuged again. The final pellet was resuspended in 5 mL PBS with 0.04% BSA to prevent cell clumping and neutrophil activation. To retain neutrophils and avoid bias, no flow cytometry-based enrichment was performed, ensuring their full representation in the dataset. Cell viability (> 90%) and concentration were confirmed using Trypan blue staining and a hemocytometer. Only high-quality suspensions were used for downstream scRNA-seq analysis.

### Library Preparation and Sequencing

We employed the 10× Genomics Chromium Single Cell 3′ Library and the Gel Bead Kit v2 to construct single-cell libraries. GEMs were generated using the Chromium 10 × Single Cell System (10 × Genomics). Cells were barcoded, followed by cDNA synthesis and library construction. Sequencing was performed on an Illumina HiSeq X Ten platform using paired-end 150 bp (PE150) reads.

During Cellranger analysis, we optimized cell-calling parameters to better capture neutrophils. Specifically, the Unique molecular identifier (UMI) count threshold was adjusted from the default ≥ 500 to a range of 100-6000. This adjustment ensured the inclusion of neutrophils with relatively low transcriptional activity, allowing for more comprehensive statistical representation of this cell population in the dataset.

### scRNA-seq data processing

For in-house data, the scRNA-seq data were meticulously processed utilizing the Seurat V5 framework 21. The quality of individual cells was rigorously controlled by two primary criteria: the number of genes detected per cell and the proportion of mitochondria-related genes expressed. Specifically, cells exhibiting fewer than 200 detected genes or a mitochondria gene proportion exceeding 10% were excluded from further analysis. Additionally, gene expression data were cleaned to retain only those genes expressed in at least three cells. The datasets from various sources were integrated using the Merge function, ensuring that only genes common to all databases were retained. Subsequent processing of the merged dataset involved a series of standard steps, including standardization, normalization, and identification of highly variable genes, all conducted using default parameters. Given the occurrence of batch effects across multiple databases, we employed the “Harmony” package 22 to mitigate these effects. The harmony components generated by this package served as substitutes for the principalcomponents (PCs) in subsequent analyses. Within the Seurat framework, the FindNeighbors function was utilized with specific parameters: reduction set to “harmony” and dims ranging from 1 to 25. This approach allowed for the calculation of similarity distances between individual cells. Subsequently, the FindClusters function was applied to perform further clustering analysis on the dimension-reduced cells. The resolution parameter was set to 0.7 for the analysis of all cells, and to 0.1 for the additional dimension reduction and clustering of neutrophils.

For publicly available data, we utilized the uniform manifold approximation and projection (UMAP) coordinates and cell annotation information obtained from the TISCH database without additional processing.

All visualizations and enrichment analyses were performed with the “scRNAtoolVis” 23 and “ggSCvis” package 24. For the analysis of intercellular communication, we employed ligand-receptor interaction data from the “CellChat” package 25. Additionally, pseudotime trajectory analysis was conducted using the “Slingshot” package 26.

### Bulk data analysis

We extracted the top 50 characterized genes from different neutrophil subpopulations and assigned scores to Neu1-3 in each public sample using the Gene Set Variation Analysis (GSVA) method built into the BEST database after excluding ribosomal genes 17. Subsequent advanced analyses have relied on BEST, with the application of default parameters.

### ST data processing

ST data were processed using the “SPACET” package 27, which allows us to annotate ST sopts in unsupervised mode. Additionally, visualizations related to gene expression, cell abundance, gene set score, and cell-cell communication score, were all generated using this package.

In order to infer histology-associated gene expression gradient, we filtered and outlined the spatial extent of data spots based on their GSVA-based Neu2 score with help of “SPATA2” package 28. In order to find, analyze, and visualize differentially expressed genes and gene sets in the spatial organization, we implemented the “SPATA2” package to construct spatial trajectories. This analysis allows to observe whether genes follow the same or different patterns along a given path.

### Transcription factor prediction

“Transcription Factor Target Finder (TFTF)” package 29 is designed for predicting transcription factor target genes and upstream transcription factors of target genes. This tool allows researchers to fully utilize the prediction results from some well-known online tools (hTFTarget, KnockTF, CHEA, TRRUST, GTRD, and ChIP Atlas) and combine them with correlation analysis to maximize the reliability of the predicted transcription factor-target gene regulatory relationships. In this study, the tool was used to predict potential upstream regulators of *MIEN1*.

### Lentiviral vector construction and transfection

Lentiviral vectors were designed and constructed by GeneChem (for human cells) and Applied Biological Materials (ABM) Inc. (for mouse cells). Detailed information on the construction of plasmids, lentivirus production, and vector components is provided in Tables S2-S11. Cells were transduced with lentivirus according to the manufacturers' protocols. Briefly, cells were seeded at an appropriate density in culture plates and incubated with lentiviral supernatant supplemented with 8 μg/mL polybrene to enhance transduction efficiency. The culture was maintained for 24-48 hours to allow for viral integration, after which cells were washed and resuspended in fresh medium. The accuracy of transduction, as well as gene knockdown and over-expression, was verified by Western blotting (WB) (Figure S2).

### Neutrophil isolation and purity assessment

Peripheral blood neutrophils from CRC patients were isolated using the EasySep™ Direct Human Neutrophil Isolation Kit following the manufacturer's protocol. Blood samples were collected in heparinized tubes and processed immediately. Peripheral blood was incubated with the EasySep™ Isolation Cocktail to deplete non-neutrophil cells, followed by the addition of Magnetic Particles. After a 5-minute incubation, the mixture was placed into the EasySep™ Magnet, and the enriched neutrophil fraction was collected. Cells were washed with ice-cold PBS (0.04% BSA) and centrifuged at 250 × g for 5 minutes at 4°C. Neutrophil isolation from colorectal tumors: Fresh CRC tissues were minced and digested in Liberase TL (0.2 mg/mL) + DNase I (50 μg/mL) for 45 min at 37°C. After filtering (40 μm) and RBC lysis, CD45+CD11b+CD66b+ neutrophils were sorted by cell sorter (Sony MA900, Sony Biotechnology, Japan). HL60 granulocytic differentiation was induced by 1.25% DMSO treatment for 5 days 30. To generate TANs, differentiated HL60 cells were exposed to a 1:1 mixture of fresh Iscove's Modified Dulbecco's Medium (IMDM) and conditioned medium from CRC cell lines. All neutrophil purity was confirmed by Giemsa staining and flow cytometry (URIT bf-730, Guilin Ulead Medical Electronics, China) (Figures S3A-C).

RNA sequencing analysis comparing primary TANs isolated from CRC tissues with HL60-induced TANs showed conserved expression of Neu2 signature genes between the two groups (Figures S3D-E). However, tissue-derived TANs exhibited partially elevated expression of certain N2 markers (Figures S3F-G). These findings collectively support the utility of the HL60 system for modeling Neu2-type TANs in tumor microenvironment studies.

### CD8+ T cell isolation and purity assessment

Human peripheral blood CD8+ T cells were isolated using CD8 MicroBeads, following the manufacturer's protocol. After Ficoll density gradient centrifugation to obtain Peripheral blood mononuclear cells (PBMCs), CD8+ T cells were magnetically separated using an MS column and a MACS Separator. The purity of the isolated CD8+ T cells exceeded 90%, as confirmed by flow cytometry (Figure S4).

## Results

### scRNA-seq revealed the microenvironment components of premalignant colorectal mucosa and cancer

**Figure 1:**
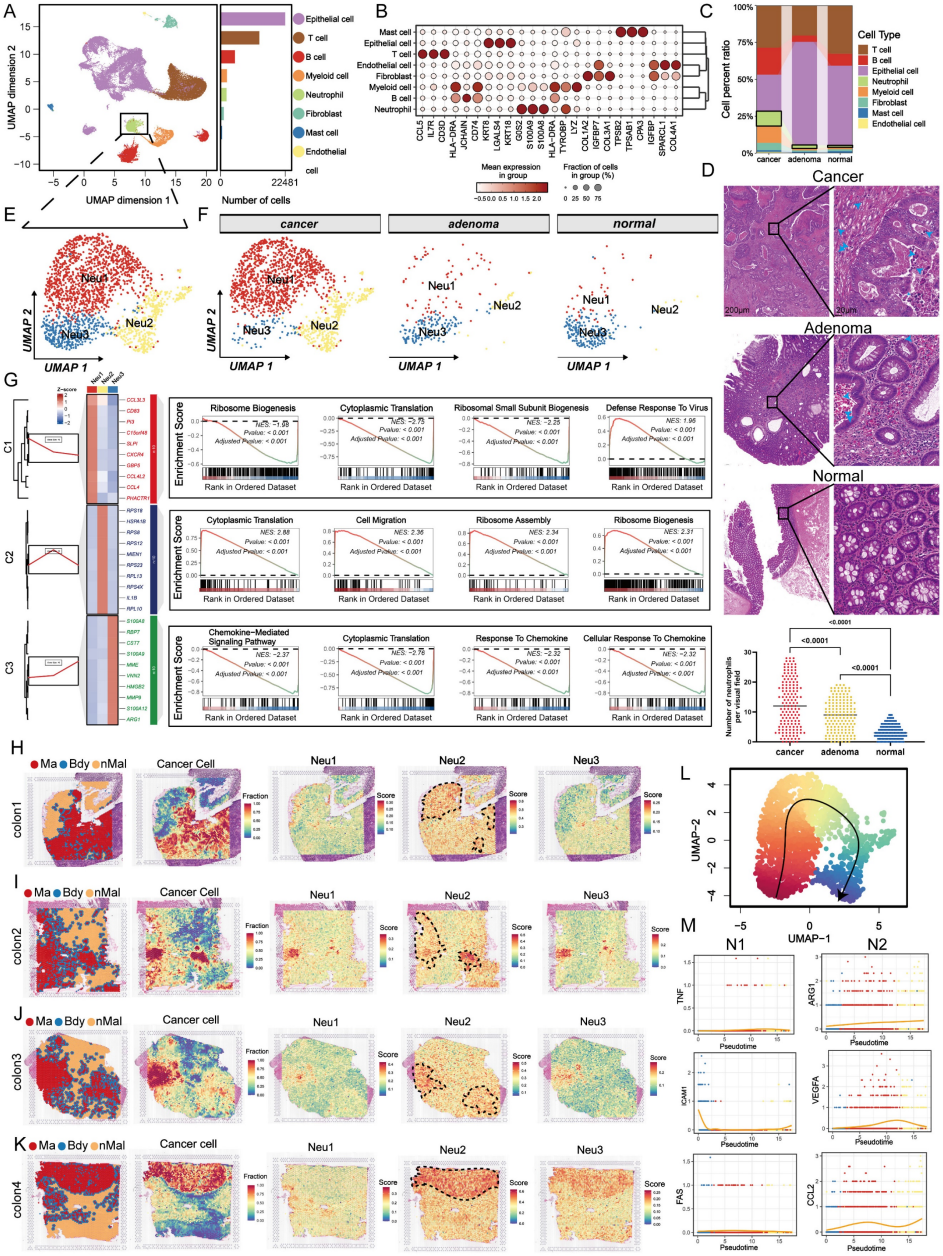
** Comprehensive analysis reveals the cellular composition and neutrophil subpopulations in colorectal cancer (CRC) progression. (A)** Uniform Manifold Approximation and Projection (UMAP) visualization of 47,210 high-quality single cells from 9 biopsies (normal, adenoma, and adenocarcinoma) collected from 3 CRC patients. Cells are color-coded based on cell type. The bar plot on the right shows the relative abundance of each cell type across all samples. **(B)** Dot plot displaying the mean expression levels (color intensity) and fraction of cells expressing key markers (dot size) for each cell type. Dot size represents the proportion of cells expressing the gene, while color indicates expression intensity. **(C)** Stacked bar plot comparing cell type proportions across cancer, adenoma, and normal tissues. **(D)** Representative HE staining images of cancer, adenoma, and normal colorectal tissues. Images are displayed at two magnifications: 200 μm (left) and 20 μm (right). Blue arrows highlight neutrophils, with quantification (bottom right) showing significantly higher neutrophil counts in cancer tissues compared to adenoma and normal tissues. **(E)** Subclustering of neutrophils reveals three distinct subpopulations (Neu1, Neu2, Neu3), visualized in a UMAP plot. Neu1 (red), Neu2 (yellow), and Neu3 (blue) exhibit unique gene expression profiles. **(F)** UMAP visualization of neutrophil subpopulations across cancer, adenoma, and normal tissues. **(G)** Functional analysis of neutrophil subpopulations. (Left) Heatmap displaying differentially expressed genes grouped into three clusters (C1-C3) for Neu1, Neu2, and Neu3. (Right) Gene Set Enrichment Analysis (GSEA) identifies biological pathways enriched in each subpopulation. Neu2 shows enhanced ribosome biogenesis, migration, and chemokine signaling. **(H-K)** Spatial transcriptomics analysis of four colon tumor samples (Colon1-Colon4). Left panels: Spatial regions classified as malignant (Ma, red), boundary (Bdy, blue), and non-malignant (nMal, yellow). Second panels: Cancer cell fractions predominantly enriched in malignant regions. Third to fifth panels: Spatial distribution and scores of Neu1, Neu2, and Neu3 subpopulations. **(L)** UMAP visualization of pseudotemporal differentiation trajectory for neutrophils. The black trajectory line indicates a linear progression from Neu3 to Neu1 to Neu2. **(M)** Pseudotime analysis of gene expression dynamics for N1 (pro-inflammatory) and N2 (pro-tumoral) markers. N1 markers (*TNF*, *ICAM1*, *FAS*) exhibit consistent expression across pseudotime, while N2 markers (*ARG1*, *VEGFA*, *CCL2*) show progressive up-regulation along the trajectory toward Neu2, supporting its tumor-promoting role.

To dissect the TME across the continuum of colorectal tumorigenesis, we performed scRNA-seq on biopsies from three patients, yielding 47,210 high-quality cells (Figure 1A). Unsupervised clustering identified eight major cell lineages: epithelial, T, B, myeloid, neutrophil, fibroblast, mast, and endothelial cells (Figure 1B). Notably, malignant tissues harbored increased proportions of neutrophils, B cells, and myeloid populations, suggesting their potential involvement in tumor progression (Figure 1C). Given the increasing attention is being paid to the role of TANs in cancer progression 9, 31, 32, we investigated neutrophil heterogeneity across normal intestine, colorectal adenomas, and CRC. Consistent with the existing evidence, HE staining revealed a stepwise increase in neutrophil infiltration along this progression (Figure 1D). We then further re-clustered neutrophils into three distinct subpopulations (Neu1-Neu3), as shown in Figures 1E-F. Notably, Neu2 was predominantly derived from cancer. Neu2 exhibited enhanced ribosome function and migration capacity, driven by distinct gene expression programs (Figure 1G and Table S12). These data suggest that Neu2 may be a tumor-specific TAN subset promoting CRC progression. Given the pivotal role of ribosomes in cellular function as the factories for protein synthesis 33, we formulated the hypothesis that Neu2 may embody an abnormally functioning, cancer-specific subset of neutrophils.

To investigate the spatial distribution of TANs, we mapped Neu1-3 subpopulations onto spatial transcriptomics (ST) data from eight CRC patients using scRNA-seq-derived gene signatures. Signature specificity was confirmed by strong correlations with canonical neutrophil markers across bulk RNA-seq datasets (Figure S5). Unbiased clustering identified malignant, boundary, and non-malignant regions (Figures 1H-K and S6). Neu2 showed consistent colocalization with malignant areas. Although in the “colon1” sample Neu2 appeared elevated in adjacent non-tumor regions, quantitative analysis across all patients revealed significantly higher Neu2 levels within tumors. In contrast, Neu1 and Neu3 were weakly expressed and lacked consistent tumor association, underscoring Neu2 as the dominant infiltrating subset. This spatial pattern was validated across nine independent bulk transcriptomic datasets, where Neu2 signature genes were recurrently upregulated in tumors (Figures S7A-D). Furthermore, Cox regression linked Neu1 and Neu2 signatures to poor prognosis in CRC (Figures S7E-G). To explore lineage dynamics, we applied pseudotime trajectory analysis using the Monocle algorithm 34, which revealed a differentiation path from Neu3 → Neu1 → Neu2 (Figure 1L). While N1 marker expression remained stable, Neu2 (differentiation end) exhibited mild up-regulation of N2-associated markers, including *ARG1*, *VEGFA*, and *CCL2* (Figure 1M), consistent with an immunosuppressive, tumor-promoting phenotype. These data identify Neu2 as the neutrophil subset most closely associated with tumor progression.

### Neu2 migrates through Migration and Invasion Enhancer 1 (MIEN1)-related mechanisms without causing inflammation

**Figure 2:**
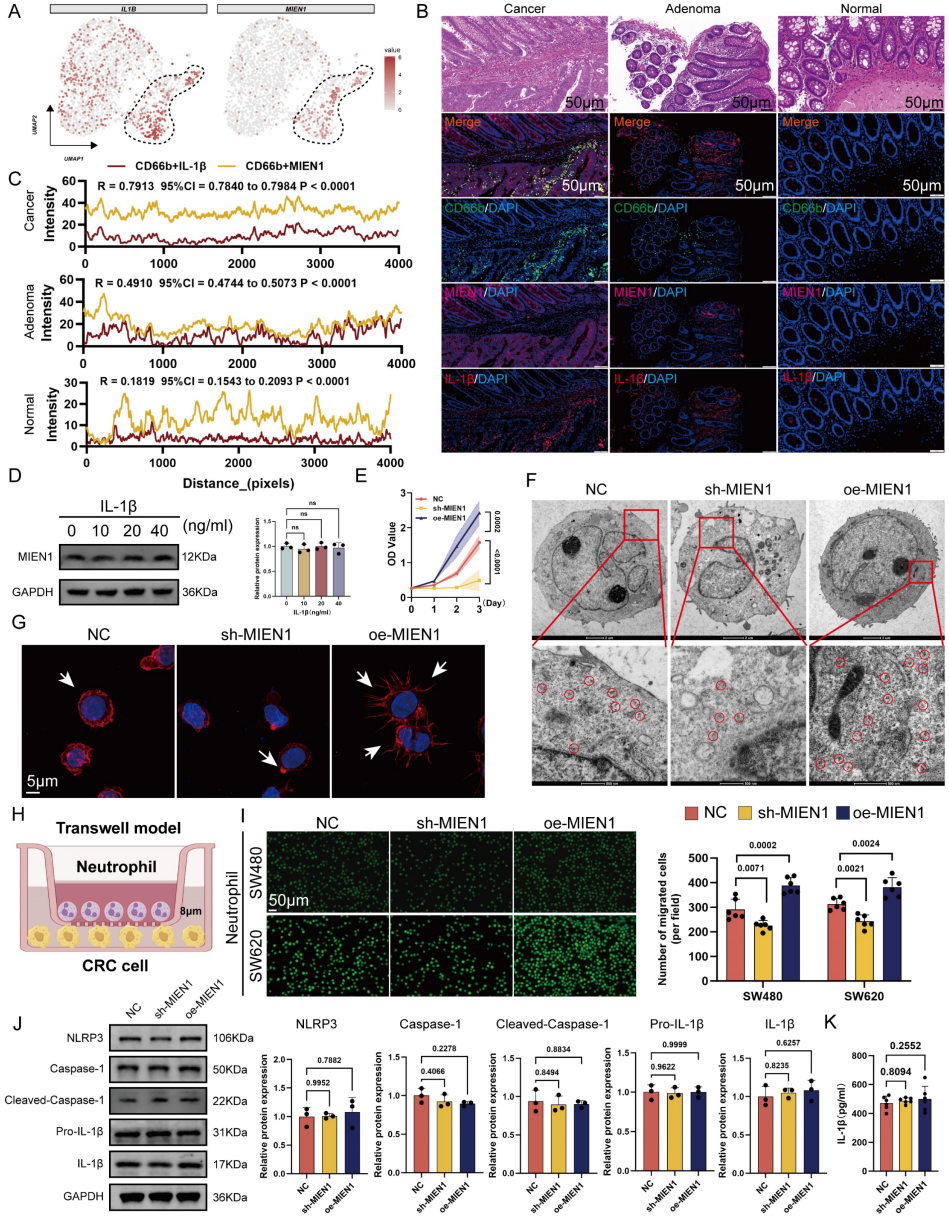
** MIEN1 promotes Neu2 migration and chemotaxis towards cancer cells without inducing inflammation. (A)** UMAP plots showing the expression patterns of *IL1B* (left) and *MIEN1* (right) within the neutrophil subpopulation. Cells with higher expression levels are colored in red, while cells with lower or no expression are shown in grey. The dashed lines outline specific regions within the Neu2 cluster exhibiting high expression of *IL1B* and *MIEN1*, highlighting their specificity to Neu2. **(B)** HE staining provides structural context, while merged fluorescence images show the co-localization of CD66b with MIEN1 and IL-1β, which are significantly elevated in cancer tissues compared to adenoma and normal tissues. Scale bar: 50 μm. **(C)** Line plots of co-localization intensity between CD66b and MIEN1 (yellow) or CD66b and IL-1β (red) across tissue distances (pixels). Cancer and adenoma tissues demonstrate stronger positive correlations compared to normal tissues, with correlation coefficients (R), 95% confidence intervals (CI), and P-values indicating statistical significance. **(D)** Western blot showing MIEN1 expression in HL60 cells treated with increasing concentrations of IL-1β (0, 10, 20, 40 ng/mL). MIEN1 levels remained unchanged, indicating IL-1β does not regulate its expression. ns = not significant (one-way ANOVA). **(E)** Growth curve of differentiated HL60 cells (neutrophils) under three conditions: NC (negative control), sh-MIEN1 (*MIEN1* knockdown), and oe-MIEN1 (*MIEN1* over-expression). oe-MIEN1 significantly enhances cell proliferation, while sh-MIEN1 reduces it. P-values indicate statistical significance. **(F)** Transmission electron microscopy (TEM) images of differentiated HL60 cells under NC, sh-MIEN1, and oe-MIEN1 conditions. Top row: Cellular ultrastructure. Scale bar: 2 μm. Bottom row: Magnified views showing the distribution of free ribosomes (highlighted by red circles). Scale bar: 500 nm. oe-MIEN1 cells exhibit an increased number of ribosomes. **(G)** Phalloidin staining of differentiated HL60 cells under NC, sh-MIEN1, and oe-MIEN1 conditions. Cells treated with oe-MIEN1 show significant cytoskeletal remodeling, including extensive pseudopodia formation (arrows), indicative of increased migratory capacity. DAPI (blue) stains nuclei. Scale bar: 5 μm. **(H)** Schematic diagram of the Transwell model used to assess neutrophil chemotaxis. Neutrophils are placed in the upper chamber, separated by an 8 μm porous membrane, allowing migration toward CRC cells seeded in the lower chamber. **(I)** Representative images (left) and quantification (right) of neutrophil migration towards SW480 and SW620 CRC cells in the Transwell assay under NC, sh-MIEN1, and oe-MIEN1 conditions. Fluorescently labeled neutrophils migrating through the membrane are visualized. Scale bar: 100 μm. oe-MIEN1 significantly increases neutrophil migration, while sh-MIEN1 reduces it. P-values indicate statistical significance. **(J)** Western blot analysis of inflammasome-related proteins, including NLRP3, Caspase-1, Cleaved-Caspase-1, Pro-IL-1β, and IL-1β, in differentiated HL60 cells under NC, sh-MIEN1, and oe-MIEN1 conditions. Bar graphs (right) show relative protein expression, with no significant changes across conditions, suggesting that MIEN1 may not influence inflammatory pathways. **(K)** Quantification of IL-1β secretion (pg/ml) in the supernatant of differentiated HL60 cells under NC, sh-MIEN1, and oe-MIEN1 conditions. No significant differences were observed, indicating that MIEN1-mediated chemotaxis does not induce IL-1β secretion or inflammatory responses.

After excluding ribosomal genes from the top 10 most enriched transcripts in Neu2,* IL1B* and *MIEN1* emerged as the most specifically up-regulated genes in this neutrophil subset (Figure 2A). Immunofluorescence analysis confirmed co-localization of MIEN1 with CD66b+ neutrophils, with a progressive increase in MIEN1+ neutrophil infiltration observed from normal mucosa to adenoma and carcinoma tissues (Figures 2B-C). These data underscored that the Neu2 may exist mainly in cancer status. Within the TME, neutrophils exert a crucial function by secreting IL-1β 35, which acts as an important autocrine signal to sustain an intense recruitment of neutrophils to promote the complex interplay of immune responses and tumor progression 36. However, as shown in Figure 2D, IL-1β stimulation at varying concentrations had no effect on MIEN1 expression in differentiated HL60 cells, suggesting that IL-1β is unlikely to act as a direct upstream regulator. To evaluate MIEN1's potential oncogenic function in neutrophils, we performed CCK-8 assays in HL60 cells with MIEN1 knockdown (sh-MIEN1) or overexpression (oe-MIEN1). Suppression of MIEN1 significantly reduced cell proliferation over time (Figure 2E). Given that prior enrichment analyses pointed to enhanced ribosomal activity in the Neu2 subset, we examined ribosome density via transmission electron microscopy. As illustrated in Figure 2F, MIEN1-overexpressing HL60 cells exhibited markedly elevated ribosome content, highlighted by red circles, suggesting a link between MIEN1 and translational capacity. Since MIEN1 is implicated in cell motility, we hypothesized that its upregulation might enhance neutrophil migration. Supporting this, confocal microscopy revealed that MIEN1 overexpression promoted pseudopod formation in HL60 cells (Figure 2G). To directly assess chemotactic behavior, we employed a Transwell co-culture system with HL60 cells seeded in the upper chamber and CRC cells in the lower compartment (Figure 2H). MIEN1 overexpression significantly enhanced HL60 recruitment toward CRC cells, whereas knockdown impaired this migration (Figure 2I). Notably, this MIEN1-driven chemotaxis did not trigger inflammatory responses: neither NLRP3 activation (Figure 2J) nor IL-1β secretion (Figure 2K) showed significant change. These findings suggest that MIEN1 facilitates neutrophil recruitment through a non-inflammatory mechanism, highlighting a distinct migratory pathway in the Neu2 subset.

### CCCTC-Binding Factor (CTCF) is a key upstream factor responsible for the unique phenotype of Neu2

**Figure 3:**
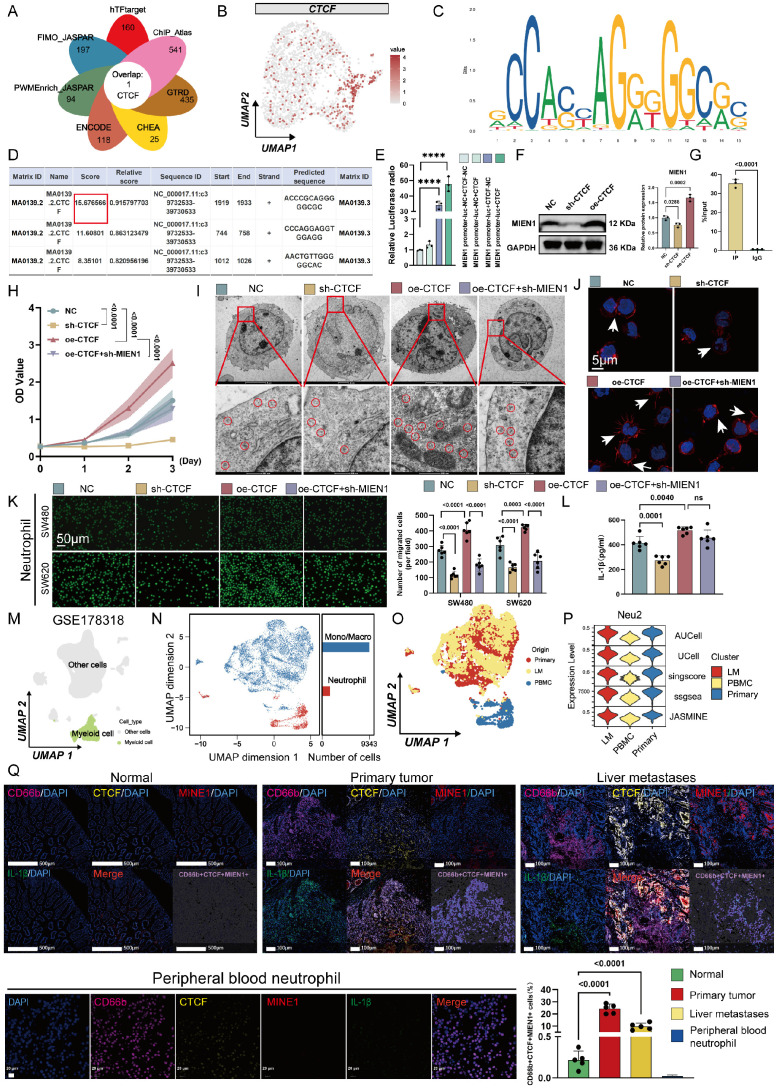
** CTCF is a key transcriptional regulator of Neu2 and promotes its functional phenotype in CRC. (A)** Venn diagram summarizing the overlap of CTCF target predictions from seven databases (hTFtarget, ChIP_Atlas, GTRD, CHEA, ENCODE, FIMO_JASPAR, and PWMEnrich_JASPAR). CTCF was identified as a common target across all databases. **(B)** UMAP plot displaying the expression pattern of *CTCF* in neutrophil populations. The color scale represents expression levels, with higher levels shown in darker red. *CTCF* expression is predominantly concentrated in the Neu2 subcluster, similar to *MIEN1* and *IL1B*. **(C-D)** Predicted CTCF binding sites on the MIEN1 promoter region: (C) Sequence logo depicting the CTCF binding motif. The height of each nucleotide reflects the conservation level at that position. (D) Table summarizing the predicted binding sites, including Matrix ID, Score, Relative score, Start/End positions, Strand, and Predicted sequence. The site with the highest score (highlighted in red) was selected for further validation. **(E)** Relative luciferase activity assay indicating the regulatory effect of *CTCF* on *MIEN1* promoter activity. The presence of CTCF significantly enhances luciferase activity, particularly in the MIEN1 promoter-luc + CTCF group, suggesting a strong positive regulatory role of *CTCF* on *MIEN1* promoter. Bars represent mean ± SD of three independent experiments. Statistical significance was analyzed using a two-tailed Student's t-test. **(F)** Western blot analysis showing MIEN1 expression levels under NC (control), sh-CTCF (*CTCF* knockdown), and oe-CTCF (*CTCF* over-expression) conditions. The bar graph (right) quantifies relative MIEN1 protein levels. Statistical analysis was performed using a one-way ANOVA with Tukey's post-hoc test. **(G)** ChIP-qPCR analysis of CTCF binding to the MIEN1 promoter region. The bar graph represents the percentage of input recovered from immunoprecipitation (IP) with CTCF antibody (IP) and control immunoglobulin G (IgG). CTCF binding is significantly enriched at the MIEN1 promoter, as indicated by the higher %Input in the IP sample compared to the IgG control. **(H)** Growth curves of differentiated HL60 cells under NC, sh-CTCF, oe-CTCF, and oe-CTCF + sh-MIEN1 conditions. Data are presented as mean ± SD. Statistical significance was determined using a two-way ANOVA with Bonferroni correction. **(I)** Transmission electron microscopy images of differentiated HL60 cells under NC, sh-CTCF, oe-CTCF, and oe-CTCF + sh-MIEN1 conditions. Top row: overall cellular ultrastructure. Scale bar: 2 μm. Bottom row: magnified views with ribosomes circled in red. Scale bar: 500 nm. **(J)** Immunofluorescence images showing cytoskeletal (red) and nuclear (blue) staining in differentiated HL60 cells under different conditions. oe-CTCF cells exhibit pronounced pseudopodia (arrows), indicating enhanced cytoskeletal remodeling, whereas MIEN1 knockdown (oe-CTCF + sh-MIEN1) reduces this effect. **(K)** Neutrophil migration assay using Transwell chambers: Representative images (left) and quantification (right) of neutrophil migration towards SW480 and SW620 CRC cells (Scale bar: 50 μm). (P values are indicated, one-way ANOVA with post hoc tests). **(L)** IL-1β secretion in differentiated HL60 cells under the same conditions. CTCF over-expression led to increased IL-1β secretion, but MIEN1 knockdown did not significantly alter IL-1β levels compared to oe-CTCF alone. Data are presented as mean ± SD. Statistical analysis was performed using a one-way ANOVA with Tukey's post-hoc test (ns = not significant). **(M-P)** Analysis of myeloid cells from the GSE178318 scRNA-seq dataset: (L) UMAP plot distinguishing myeloid cells (green) from other cell types (gray). (M) UMAP plot showing clustering of monocytes/macrophages (blue) and neutrophils (red). Bar chart quantifies the relative abundance of each cell type. (N) UMAP plot depicting the distribution of cells from different origins: Primary tumor (red), liver metastasis (yellow), and PBMC (blue). (O) Violin plots showing Neu2 scores across primary tumors, liver metastases, and PBMCs, based on five scoring methods (AUCell, UCell, singscore, ssgsea, JASMINE). **(Q)** mIF analysis of normal intestinal epithelium, CRC primary tumors, liver metastases, and CRC patient peripheral blood neutrophils using the HALO platform. Panels (Top Row): Representative mIF images of normal tissue, CRC primary tumors, and liver metastases stained for CD66b (magenta, neutrophil marker), CTCF (yellow), MIEN1 (red), IL-1β (green), and nuclei (DAPI, blue). The merge panels combine all markers, with CD66b+CTCF+MIEN1+ triple-positive cells highlighted in the composite image. Panel (Bottom Left): Representative mIF images of Peripheral blood neutrophil stained for the same markers as tissue samples. Scale bars: 20 μm. Panel (Bottom Right): Quantification of CD66b+CTCF+MIEN1+ triple-positive cells as a percentage of total cells in the field of view. In primary tumors (red) and liver metastases (yellow), triple-positive cells are significantly enriched compared to normal tissue (green). Importantly, peripheral blood neutrophil exhibit negligible percentages of triple-positive cells, underscoring their near absence in circulation. Statistical analysis was performed using one-way ANOVA with Tukey's test.

Neutrophil differentiation involves tightly regulated transcriptional programs 37, 38. To uncover potential upstream regulators of the Neu2 phenotype, we integrated predictions from seven transcriptional regulatory databases, which converged on CTCF as a putative transcription factor targeting *MIEN1*. scRNA-seq data confirmed robust *CTCF* expression within Neu2 cells. JASPAR analysis identified high-affinity CTCF binding motifs on the *MIEN1* promoter (Figures 3A-B); the top-scoring site was selected for downstream validation (Figures 3C-D, red box). Dual-luciferase assays confirmed CTCF-driven transcriptional activation of *MIEN1* (Figure 3E), while Western blot analysis demonstrated that CTCF overexpression elevated MIEN1 protein levels, whereas knockdown suppressed it (Figure 3F). ChIP-qPCR further verified direct binding of CTCF to the predicted site: fragments containing the motif were significantly enriched in CTCF-ChIPed DNA but not in IgG controls (Figure 3G). Functionally, CTCF modulated neutrophil survival, ribosomal function, and recruitment toward tumor cells through MIEN1 (Figures 3H-K). Notably, CTCF knockdown reduced IL-1β expression in neutrophils (Figure 3L), consistent with its reported role in hepatocytes 39. To further explore the broader role of CTCF in TANs, we performed RNA-sequencing and revealed CTCF-driven signaling networks for immune response, cell cycle, and metabolism (Figure S8).

To characterize the presence of the Neu2 phenotype in CRC, we analyzed an independent scRNA-seq dataset (GSE178318). Myeloid cells were reclustered into Monocyte/Macrophage and Neutrophil populations (Figures 3M-O). Neu2 scores, calculated using five algorithms, were significantly elevated in primary and metastatic tumors compared to PBMCs (Figure 3P). Multiplex immunofluorescence (mIF) staining of clinical CRC specimens further confirmed that CTCF-MIEN1-IL-1β-positive Neu2 cells were enriched in tumor tissues (Figure 3Q), supporting their tumor-specific role.

### Hypoxia as an initiating factor for Neu2 features

**Figure 4:**
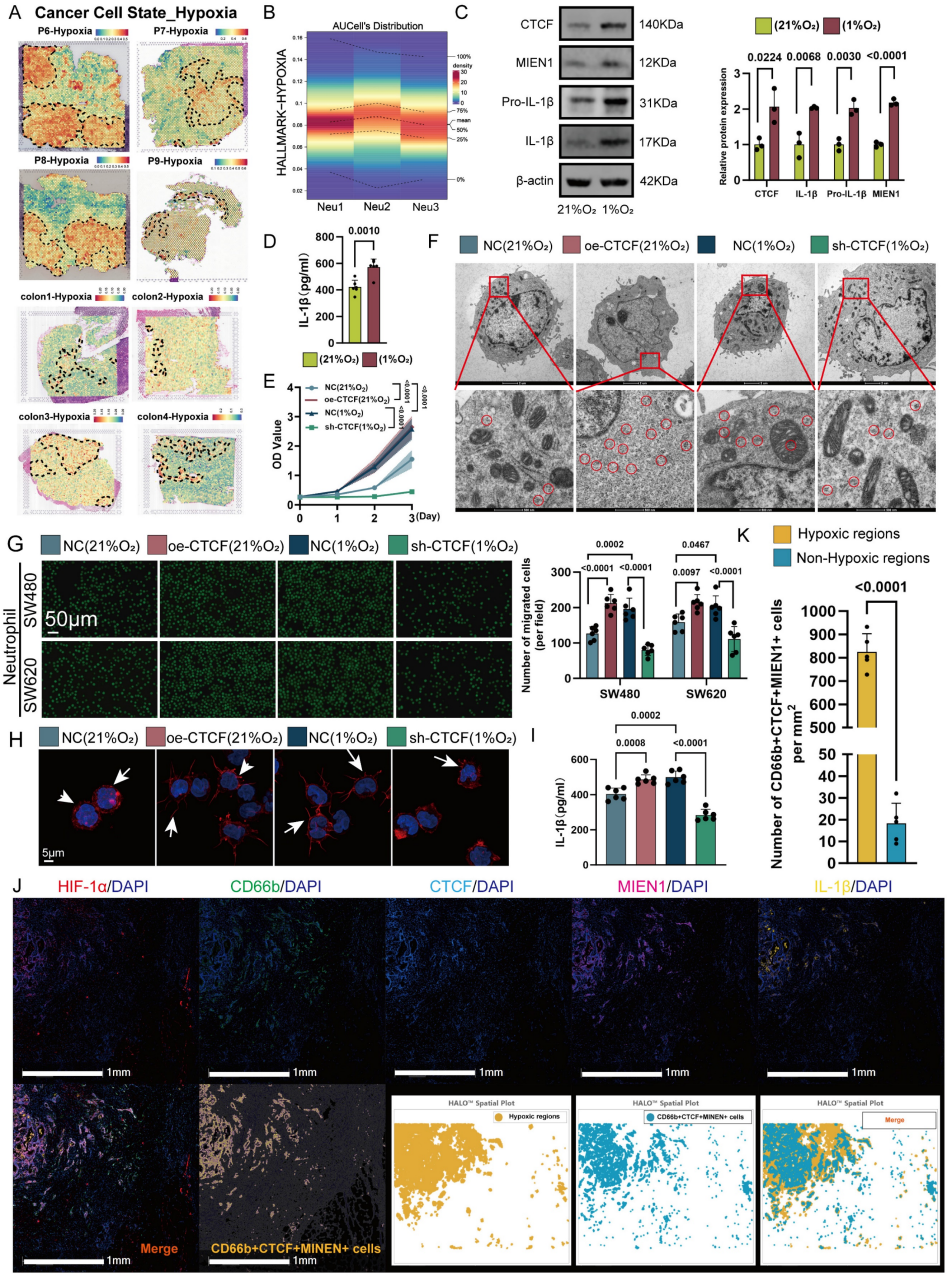
**Hypoxia induces Neu2 phenotype through CTCF-related signaling in CRC. (A)** ST analysis demonstrating co-localization of hypoxia-enriched regions and Neu2 signatures in colorectal cancer tissues. Top panel (P6-P9): Hypoxia enrichment scores were calculated using the “Cancer Cell State - Hypoxia” gene set and visualized with a blue-to-red gradient, where red represents higher hypoxia levels. Neu2 regions (outlined in dashed lines) spatially overlap with hypoxia-enriched areas. Bottom panel (Colon1-Colon4): Similar spatial analysis confirms the consistent co-localization of Neu2 with hypoxia-enriched regions across multiple tumor sections. **(B)** Heatmap showing hypoxia-related scores for neutrophil subpopulations (Neu1, Neu2, Neu3) based on “Hallmark Hypoxia” gene set enrichment analysis using AUCell. Rows represent individual cells, and columns represent hypoxia-related scores. Neu2 subpopulation exhibits significantly higher hypoxia scores compared to Neu1 and Neu3, consistent with its spatial association with hypoxic regions. **(C-D)** Hypoxia up-regulates CTCF-MIEN1-IL-1β signaling in differentiated HL60 cells: (C) Western blot showing increased expression of CTCF, MIEN1, Pro-IL-1β, and IL-1β under hypoxic (1% O₂) compared to normoxic (21% O₂) conditions. Relative protein expression is quantified in the bar graph (right). (D) Quantification of IL-1β secretion in the supernatant of differentiated HL60 cells under normoxic and hypoxic conditions, showing a significant increase under hypoxia (two-tailed Student's t-test). **(E)** Growth curves of differentiated HL60 cells under normoxia (NC, oe-CTCF) and hypoxia (NC, sh-CTCF) conditions. *CTCF* over-expression (oe-CTCF) significantly enhances cell proliferation under normoxia, while *CTCF* knockdown (sh-CTCF) suppresses growth under hypoxia (two-way ANOVA). **(F)** Transmission electron microscopy (TEM) images of differentiated HL60 cells under normoxia (NC, oe-CTCF) and hypoxia (NC, sh-CTCF). Top row: Overview of cellular ultrastructure (scale bar: 2 μm). Bottom row: Magnified views showing ribosomes (circled in red, scale bar: 500 nm). Increased ribosome abundance is observed under hypoxia and in cells with oe-CTCF. **(G)** Migration assay of differentiated HL60 cells towards SW480 and SW620 colorectal cancer cells in a Transwell system. Representative fluorescent images (top) and bar graph (bottom) quantifying migrated cells under different conditions. *CTCF* over-expression promotes migration under normoxia, while *CTCF* knockdown reduces migration under hypoxia (one-way ANOVA). Scale bar: 50 μm. **(H)** Immunofluorescence images of differentiated HL60 cells stained for cytoskeletal proteins (red) and nuclei (blue). White arrows indicate pseudopodia, which are more pronounced under oe-CTCF conditions. Scale bar: 5 μm. **(I)** Quantification of IL-1β levels in the supernatant of differentiated HL60 cells under normoxia and hypoxia conditions. *CTCF* over-expression increases IL-1β secretion under normoxia, while *CTCF* knockdown reduces secretion under hypoxia (one-way ANOVA).** (J)** HALO analysis platform was used to evaluate CRC specimens, classifying regions with high HIF-1α expression as hypoxic and regions with low HIF-1α expression as relatively normoxic. mIF revealed the spatial distribution of HIF-1α (red), CD66b (green), CTCF (blue), MIEN1 (magenta), IL-1β (yellow), and DAPI (nuclei, white). The merged image highlights hypoxic regions enriched with CD66b+CTCF+MIEN1+ triple-positive cells. HALO spatial plots further delineate hypoxic regions (yellow) and the distribution of triple-positive cells (cyan). Scale bar: 1 mm. **(K)** Density of CD66b+CTCF+MIEN1+ cells in hypoxic and non-hypoxic regions. Bar plot showing the density of CD66b+CTCF+MIEN1+ cells (per mm²) in hypoxic regions (yellow) versus non-hypoxic regions (blue). Hypoxic regions exhibit a significantly higher density of these cells compared to non-hypoxic regions (P < 0.0001).

The intricate interplay between the CTCF transcription factor and tumor hypoxia constitutes a fascinating area of investigation within the broader landscape of cancer biology 40. Emerging evidence suggests that CTCF may be intricately involved in the adaptive responses of cancer cells to hypoxic stress 41. By regulating the expression of genes implicated in metabolic reprogramming, angiogenesis, and cell survival, CTCF could potentially facilitate the resilience of cancer cells to hypoxic conditions. Furthermore, the interaction between CTCF and hypoxia-responsive pathways, such as those governed by hypoxia-inducible factors (HIFs) 40, may unveil novel mechanisms underlying tumor progression and resistance to therapeutic interventions. Thus, Neu2, characterized by CTCF positivity, may represent a responsive mechanism to hypoxia in solid tumors. We next analyzed the distribution of hypoxic signal scores in the TME using ST data. Malignant CRC tissues exhibited elevated hypoxia signaling, with Neu2 TANs localized to hypoxic regions (Figure 4A). scRNA-seq confirmed higher hypoxia levels in Neu2 compared to Neu1 and Neu3 (Figure 4B). To confirm the induction of CTCF-MIEN1-IL-1β signaling by hypoxia in TANs, we cultured HL60-derived TANs under hypoxic (1% O₂) versus normoxic (21% O₂) conditions for 24 hours. Western blot analysis showed increased expression of CTCF, MIEN1, pro-IL-1β, and mature IL-1β, with elevated IL-1β secretion in cell supernatants (Figures 4C-D).

Given that HIF-1α is a key transcription factor in hypoxic responses 42, we examined its role in regulating CTCF. siRNA-mediated knockdown of HIF-1α markedly reduced CTCF protein levels in hypoxic HL60-derived TANs (Figure S9A). Functionally, CTCF overexpression in TANs enhanced cytoskeletal remodeling, ribosome biogenesis, and migration toward CRC cells—effects that were attenuated by HIF-1α silencing under hypoxia (Figures S9B-D), suggesting that HIF-1α-dependent CTCF induction promotes the Neu2 phenotype. This aligns with previous reports that HIF-1α binds the CTCF promoter and activates its transcription under hypoxic conditions 40. Notably, CTCF overexpression under normoxia recapitulated Neu2-like features, while its knockdown under hypoxia reversed them, including impaired migration and reduced ribosomal activity (Figures 4E-I). To further dissect this regulatory axis, we performed transcriptomic profiling of CTCF-knockdown TANs under hypoxia. The results revealed coordinated downregulation of Neu2-associated markers and alterations in immune and metabolic pathways (Figure S10). mIF confirmed that CTCF, MIEN1, and IL-1β expression positively correlated with HIF-1α levels in CRC tissues, with high-HIF-1α regions exhibiting strong co-expression of these markers (Figures 4J-K). Collectively, these findings support a model in which hypoxia-driven HIF-1α upregulates CTCF, thereby orchestrating the Neu2 phenotype within the TME.

### Neu2 enhances the invasive phenotype of cancer cells

**Figure 5:**
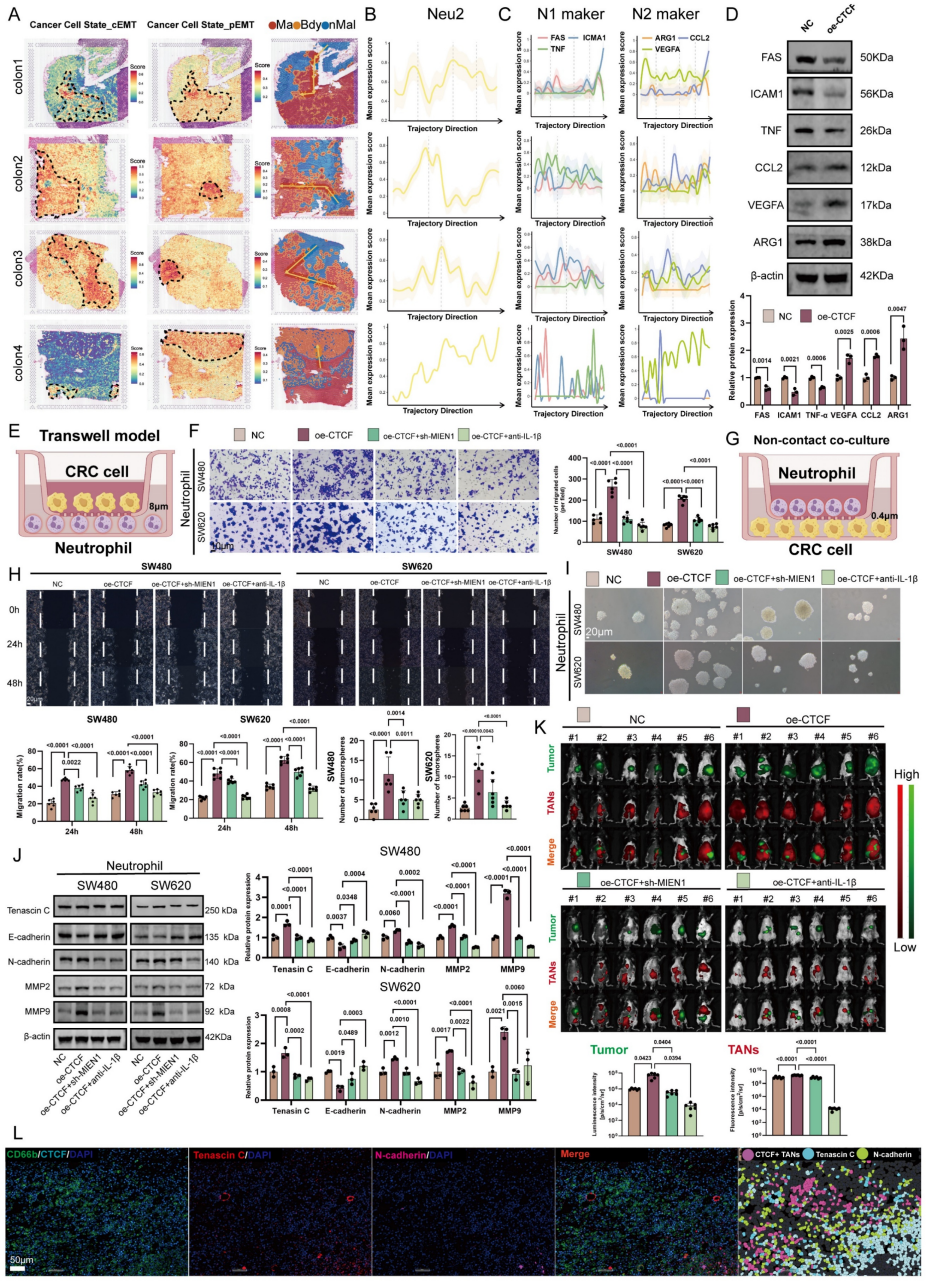
**Neu2 promotes EMT progression and enhances tumor growth through CTCF-MIEN1-IL-1β signaling. (A)** Spatial Transcriptomics of CRC Specimens. The analysis was performed on four colorectal cancer (CRC) tissue samples (Colon1-Colon4). On the left, spatial maps of complete epithelial-to-mesenchymal transition (cEMT) scores were generated using the “Cancer Cell State_cEMT” gene set, highlighting regions with strong epithelial-to-mesenchymal transition signatures. In the middle, partial EMT (pEMT) maps, based on the “Cancer Cell State_pEMT” gene set, show areas with intermediate epithelial and mesenchymal features. On the right, the tumor regions are classified into malignant areas (Ma, red), boundary zones (Bdy, yellow), and non-malignant regions (nMal, blue), with yellow arrows indicating predicted evolutionary trajectories of tumor progression from non-malignant to malignant states. This detailed categorization allows visualization of the heterogeneity in EMT progression across different regions of the tumor microenvironment. **(B)** Neu2 Expression Along Malignant Trajectories. This panel shows the expression of Neu2-related genes along spatial trajectories in the four CRC samples. The y-axis represents the average expression level of Neu2-associated genes, while the x-axis traces the transition from non-malignant to malignant regions. The data are represented by yellow lines for the mean expression values, with shaded regions indicating the 95% confidence intervals. Vertical dotted lines highlight significant transitions in the gene expression profile across the tissue regions. These results illustrate how Neu2 is spatially regulated in the tumor microenvironment. **(C)** Neutrophil Polarization in Tumor Progression. Expression of canonical N1- and N2-type neutrophil markers (*FAS*, *ICAM1*, *TNF* for N1 and *ARG1*, *VEGFA*, *CCL2* for N2) was assessed across spatial trajectories. The data show a decrease in N1 markers and an increase in N2 markers as the trajectory progresses from non-malignant to malignant regions. These findings suggest that the tumor microenvironment shifts towards a pro-tumorigenic, immunosuppressive N2 phenotype as the tumor progresses. The shaded areas represent the 95% confidence intervals, helping to visualize the robustness of the observed trends. **(D)** Western Blot Analysis of N1 and N2 Markers. Western blotting was performed on differentiated HL60 neutrophil-like cells under negative control (NC) and CTCF overexpression (oe-CTCF) conditions. The upper panel shows representative blots for N1 (FAS, ICAM1, TNF) and N2 (ARG1, VEGFA, CCL2) markers. Quantification (lower panel) reveals a shift from N1 to N2 phenotype upon CTCF overexpression (three independent experiments, one-way ANOVA). **(E)** Schematic illustration of the transwell migration model showing the co-culture system of CRC cells and neutrophils. CRC cells were seeded in the upper chamber with an 8μm pore membrane, while neutrophils were placed in the lower chamber. **(F)** Representative images and quantification of CRC cell invasion in the transwell assay. SW480 and SW620 cells were co-cultured with differentiated HL60 cells under four conditions: NC, oe-CTCF, oe-CTCF + sh-MIEN1 (CTCF overexpression with MIEN1 knockdown), and oe-CTCF + anti-IL-1β (CTCF overexpression with IL-1β neutralization). Scale bar: 10 μm. Quantification shows the number of invading CRC cells; data represent mean ± standard deviation from three independent experiments; statistical analysis by one-way ANOVA. **(G)** Schematic of the non-contact co-culture system between neutrophils and CRC cells, designed to assess CRC migration and stemness effects without direct cell-cell contact. **(H)** Wound healing assay evaluating CRC cell migration at 0 h, 24 h, and 48 h under the four treatment conditions. White lines indicate wound edges. Scale bar: 20 μm. **(I)** Tumor sphere formation assay assessing CRC stemness under the four treatment conditions. Representative images of spheres formed by SW480 and SW620 cells; scale bar: 20 μm. **(J)** Western blot analysis of EMT-related markers in CRC cells co-cultured with differentiated HL60 cells under NC, oe-CTCF, oe-CTCF + sh-MIEN1, and oe-CTCF + anti-IL-1β conditions. β-actin was used as a loading control. Quantification was based on three independent experiments; statistical analysis by one-way ANOVA. **(K)** In vivo imaging of orthotopic CRC mouse models showing primary tumor growth (green signal from luminescent CRC cells) and infiltration of DiR-labeled ER-Hoxb8-derived neutrophils (red fluorescence). Six mice were included per treatment group. Representative merged images show colocalization of tumor and neutrophil signals. Quantitative bar graphs display tumor burden (luminescence intensity) and neutrophil infiltration (DiR fluorescence intensity) across groups. Statistical analysis was performed using one-way ANOVA with Tukey's multiple comparisons test. **(L)** Using the HALO analysis platform, CRC specimens were analyzed to determine the spatial distribution of CTCF+ tumor-associated neutrophils (TANs) and the expression of epithelial-mesenchymal transition (EMT) markers. Immunofluorescence staining revealed CD66b+CTCF+ cells, partial EMT (pEMT) marker Tenascin C, complete EMT (cEMT) marker N-cadherin, and DAPI for nuclei (blue). The HALO spatial plot (right) visualizes the spatial proximity of CTCF+ TANs (pink) to Tenascin C (cyan) and N-cadherin (light green), revealing the association of neutrophils with pEMT and cEMT within the tumor microenvironment. Scale bar: 50 µm.

Neutrophil-mediated signaling is known to induce epithelial-to-mesenchymal transition (EMT) in cancer cells 43, a dynamic process involving progressive loss of epithelial traits and gain of mesenchymal properties 44-46. EMT exists along a continuum 47, from partial EMT (pEMT) 48, where cells retain some epithelial features, to complete EMT (cEMT), marked by full mesenchymal transformation 49. To explore the role of Neu2 TANs in CRC progression, we mapped cancer cell trajectories using a pseudo-temporal spatial algorithm (Figure 5A). Cancer cells transitioned from normal tissue through pEMT to cEMT within tumors. Gene expression analysis revealed that N1 markers (*FAS*, *ICAM1*, *TNF*) negatively correlated with this trajectory, whereas Neu2 and N2 markers (*ARG1*, *CCL2*, *VEGFA*) showed positive correlations (Figures 5B-C), suggesting Neu2 drives EMT, resembling N2 TANs. In HL60-derived TANs, CTCF up-regulation reduced N1 markers and increased N2 markers (Figure 5D).

We next assessed Neu2's pro-tumor effects in CRC cells co-cultured with HL60-derived TANs. CTCF over-expression enhanced CRC invasiveness, an effect blocked by MIEN1 over-expression or IL-1β neutralization (Figures 5E-F). Similarly, CTCF-MIEN1-IL-1β signaling amplified CRC migration and stemness in wound healing and sphere formation assays (Figures 5G-I). Spatial transcriptomics revealed peak Neu2 expression during pEMT, prompting us to investigate its role in this phenotype. CTCF up-regulation increased mesenchymal markers (Tenascin C, N-cadherin, MMP2, MMP9) and suppressed E-cadherin, effects reversed by sh-MIEN1 or IL-1β neutralization (Figure 5J).

In an orthotopic CRC mouse model, CTCF-over-expressing TANs increased tumor growth and TAN infiltration, which was attenuated by MIEN1 knockdown or IL-1β neutralization (Figure 5K). Multiplex immunofluorescence confirmed these findings (Figure 5L). Together, these data establish that Neu2 promotes CRC invasiveness and tumor growth through CTCF-MIEN1-IL-1β signaling, particularly during pEMT.

### Neu2 promotes liver metastasis of tumors

**Figure 6:**
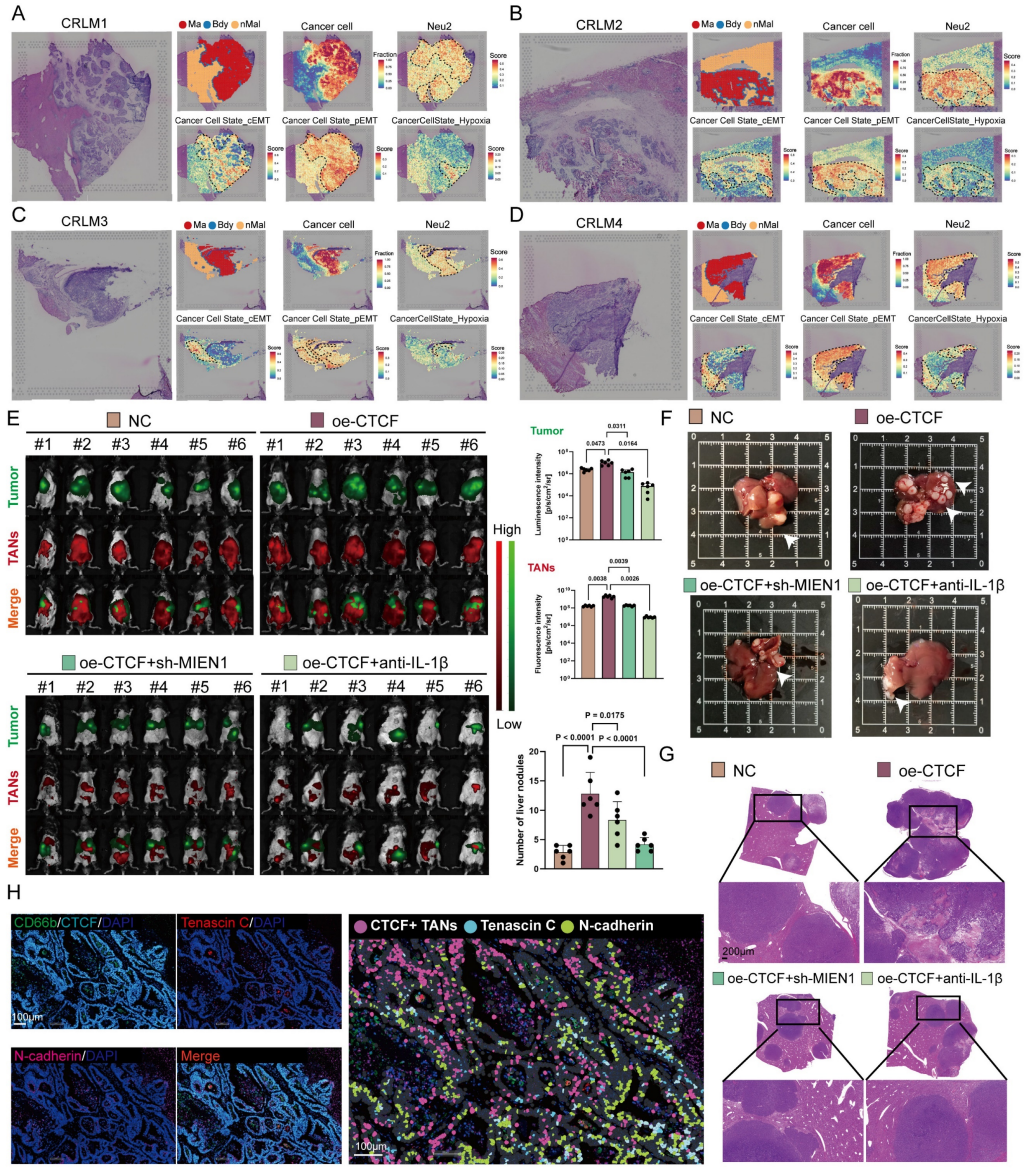
** Neu2 promotes liver metastasis by driving EMT in CRC. (A-D)** Spatial transcriptomics analysis of colorectal cancer liver metastasis samples CRLM1 (A), CRLM2 (B), CRLM3 (C), and CRLM4 (D). Left panels: HE staining of liver metastasis sections. Middle panels: Spatial distribution of distinct regions: malignant (Ma), boundary (Bdy), and non-malignant (nMal), along with cancer cell populations and Neu2 neutrophil signatures. Bottom panels: Spatial mapping of cancer cell states, including complete EMT (cEMT), partial EMT (pEMT), and hypoxia signatures. Score bars indicate relative expression levels, with higher scores reflecting stronger feature enrichment. **(E)** In vivo bioluminescence imaging of liver metastasis models. Liver metastases were established by injecting mc38-luc cells into the spleen to promote metastatic seeding in the liver. One week later, DiR iodide-labeled ER-Hoxb8-DNs were administered intraperitoneally under different treatment conditions. Neutrophils were injected every three days, and bioluminescence imaging was performed on day 28 after intrasplenic vein injection. **(F-G)** Gross liver morphology and HE staining of liver metastases in each treatment group. Upper panels: Representative gross images of livers with metastatic nodules (indicated by white arrowheads). (G) H&E-stained sections showing histological features of metastatic nodules. Scale bar: 200 μm. Bar graphs display the number of liver metastatic nodules across treatment groups. Statistical significance was determined using one-way ANOVA with Tukey's multiple comparisons test. **(H)** mIF staining and HALO platform analysis of colorectal cancer liver metastases to investigate the spatial relationship between CTCF+ TANs and EMT markers. Left panels show individual and merged fluorescence signals, while the right panel highlights spatial distribution. Scale bar: 100 µm.

pEMT enhances tumor invasiveness and plasticity by allowing cells to retain epithelial traits while acquiring mesenchymal features, ultimately contributing to metastasis and poor prognosis 50-52. Having established Neu2's role in EMT regulation, we next examined its involvement in CRLM. ST analysis of CRLM samples from four patients revealed co-localization of Neu2 and pEMT signatures within metastatic foci areas (Figures 6A-D), implying a tumor-promoting role for Neu2 in metastatic progression.

To test this hypothesis, we employed a mouse model of CRLM by injecting luciferase-labeled MC-38 cells into the spleen, followed by intraperitoneal delivery of DiR-labeled ER-Hoxb8-derived neutrophils (DNs) under various treatment conditions. Up-regulation of CTCF in ER-Hoxb8-DNs increased liver metastases and TANs; these effects were partially reversed by sh-MIEN1 or anti-IL-1β treatment (Figure 6E). Histological analysis further confirmed that Neu2 significantly increased the number of liver metastatic nodules (Figures 6F-G). Supporting these findings, mIF staining of CRLM tissues showed that N-cadherin/Tenascin C double-positive cells—indicative of pEMT-accumulated around Neu2+ neutrophils marked by CD66b, CTCF, MIEN1, and IL-1β (Figure 6H).

### Neu2 mediates stromal cell activation

To investigate whether Neu2 contributes to TME remodeling during CRC progression, we used the CellChat package to compare intercellular communication networks across normal, adenoma, and cancer tissues. Cancer samples exhibited the most extensive and complex signaling landscapes, with notably intensified stromal interactions (FiguresS11A-C). Neutrophils are known to interact robustly with fibroblasts and macrophages, promoting a tumor-permissive microenvironment via EMT induction, matrix remodeling, and immune suppression 53-56. Further analysis revealed that cancer-associated fibroblasts (CAFs) and tumor-associated macrophages (TAMs) in CRC tissues received markedly stronger Nicotinamide phosphoribosyl transferase (NAMPT) signaling compared to normal or adenoma tissues (Figures S12-S13).

**Figure 7:**
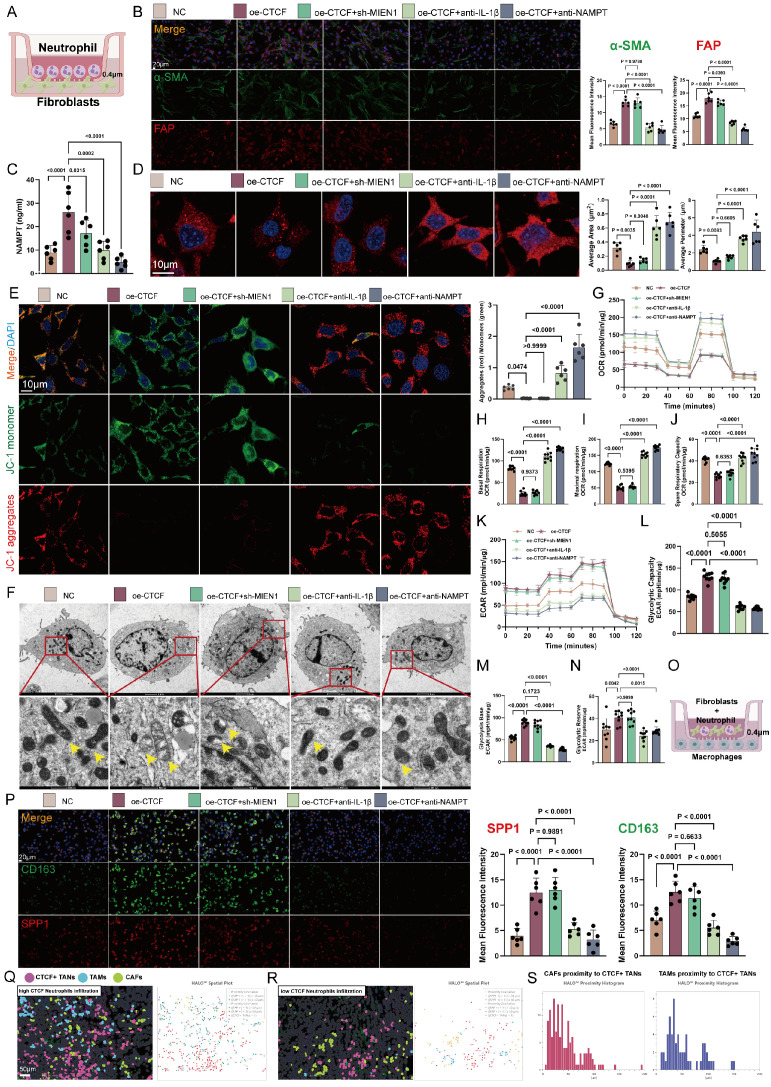
** Neu2 facilitates stromal cell activation and metabolic reprogramming in CRC. (A)** Schematic illustration of the co-culture system using fibroblasts and differentiated HL60 cells separated by a 0.4μm transwell insert. **(B)** Representative immunofluorescence images showing fibroblast activation markers α-SMA (green) and FAP (red) in fibroblasts co-cultured with differentiated HL60 cells treated under different conditions (NC, oe-CTCF, oe-CTCF + sh-MIEN1, oe-CTCF + anti-IL-1β, and oe-CTCF + anti-NAMPT). Scale bar: 20 μm. Right panels: Quantification of fluorescence intensity for α-SMA and FAP, demonstrating increased expression in the oe-CTCF group, which was significantly reduced by rescue treatments (one-way ANOVA). **(C)** ELISA quantification of extracellular NAMPT levels, demonstrating significantly increased NAMPT secretion with *CTCF* over-expression, which was suppressed by rescue treatments (one-way ANOVA). **(D)** Representative images of mitochondrial morphology in fibroblasts visualized by mitochondria-specific fluorescent probe after co-culture with differently treated differentiated HL60 cells. Blue indicates DAPI nuclear staining, and red shows mitochondrial network. Right panels show quantification of average mitochondrial area and perimeter in fibroblasts. Scale bar: 10μm. **(E)** Mitochondrial membrane potential analysis of fibroblasts after co-culture with differently treated differentiated HL60 cells using JC-1 staining. Representative merged images (top) show JC-1 monomer (green) indicating depolarized mitochondria, and J-aggregates (red) indicating polarized mitochondria, with DAPI nuclear counterstain (blue). Scale bar: 10μm. Right panel shows quantification of the red/green fluorescence ratio. Quantitative analysis of mitochondrial membrane potential using JC-1 staining, presented as the ratio of aggregates (red) to monomers (green) (one-way ANOVA). **(F)** Transmission electron microscopy (TEM) analysis confirming mitochondrial morphological changes in fibroblasts after co-culture. Yellow arrows indicate representative mitochondria. Higher magnification images (bottom row) highlight detailed ultrastructural changes in mitochondria morphology under different treatment conditions. Top row: Scale bar: 2 μm. Bottom row: Scale bar: 500 nm. **(G-J)** Seahorse Oxygen consumption rate (OCR) analysis shows that *CTCF* over-expression (oe-CTCF) in HL60-derived neutrophils significantly suppresses mitochondrial respiration in co-cultured fibroblasts. This is reflected by reduced basal respiration (H), maximal respiration (I), and spare respiratory capacity (J) in the oe-CTCF group. Neutralization of IL-1β (oe-CTCF + anti-IL-1β) or NAMPT (oe-CTCF + anti-NAMPT) restores mitochondrial function, while *MIEN1* knockdown (oe-CTCF + sh-MIEN1) has no significant effect. Statistical significance was determined using one-way ANOVA with Tukey's multiple comparisons test. **(K-N)** Seahorse ECAR analysis demonstrates that *CTCF* over-expression (oe-CTCF) in HL60-derived neutrophils significantly enhances glycolytic capacity (L), glycolysis base (M), and glycolytic reserve (N) in co-cultured fibroblasts. Neutralization of IL-1β or NAMPT effectively reverses these effects, while *MIEN1* knockdown (oe-CTCF + sh-MIEN1) shows no significant impact. Statistical analysis was performed using one-way ANOVA with Tukey's multiple comparisons test. **(O)** Schematic representation of the non-contact co-culture system. Macrophages were seeded in the lower chamber, separated by a 0.4 μm porous membrane, while HL60-derived neutrophils and fibroblasts were cultured in the upper chamber. **(P)** Representative immunofluorescence images of macrophages cultured under different conditions in a non-contact co-culture system. Cells were stained for CD163 (green) and SPP1 (red), with nuclei counterstained using DAPI (blue). Scale bar: 20 μm. Quantification of mean fluorescence intensity (MFI) for CD163 and SPP1 was performed using one-way ANOVA followed by Tukey's post hoc test for statistical analysis. **(Q-R)** Spatial comparison of colorectal cancer specimens with high (Q) and low (R) CTCF neutrophil infiltration, based on HALO platform analysis of mIF-identified cell populations. Left panels depict cell distributions with CTCF+ TANs (pink), TAMs (blue), and CAFs (green). High CTCF infiltration (Q) shows denser clustering and closer spatial proximity among these cell types, indicating enhanced stromal activation. In contrast, low CTCF infiltration (R) reveals sparser distributions and weaker stromal interactions. Right panels display HALO spatial plots, with solid lines indicating proximity between cell types. High CTCF infiltration (Q) highlights stronger and more frequent interactions among CTCF+ TANs, TAMs, and CAFs, while low CTCF infiltration (R) reflects fewer and weaker tumor-stroma connections. Scale bar: 50 μm. **(S)** Proximity histograms based on HALO platform analysis of colorectal cancer specimens, illustrating the spatial relationship of CAFs (left) and TAMs (right) to CTCF+ TANs. The left histogram shows that CAFs predominantly cluster within 50 μm of CTCF+ TANs, indicating a close spatial association that reflects stromal activation. The right histogram highlights a similar pattern for TAMs, with the majority residing within 50 μm of CTCF+ TANs, suggesting strong interactions between these immune cells and the tumor-associated neutrophils. Both proximity distributions underline the role of CTCF+ TANs in shaping the tumor microenvironment.

We first examined how neutrophils influence fibroblast behavior. In a co-culture model (Figure 7A), CTCF overexpression in HL60-derived TANs induced a prototypical CAF phenotype, characterized by increased expression of α-SMA, FAP, COL1A1, and GJA4. These effects were abrogated by IL-1β or NAMPT blockade (Figure 7B and S14). Additionally, CTCF enhanced NAMPT secretion (Figure 7C), implicating paracrine signaling.

CAF activation is closely tied to metabolic reprogramming, particularly a shift from oxidative phosphorylation to glycolysis, driven by reactive oxygen species (ROS) accumulation and calcium dysregulation 58, 59. This glycolytic switch supports a tumor-permissive microenvironment and contributes to tumor progression, making it a promising therapeutic target 60. In our model, CTCF-overexpressing TANs disrupted mitochondrial integrity in CAFs, causing fragmentation and reduced membrane potential, as visualized by microscopy and JC-1 staining—effects reversed by IL-1β or NAMPT inhibition (Figures 7D-F). Seahorse analysis confirmed suppressed mitochondrial respiration—including reduced basal/maximal oxygen consumption rate (OCR), ATP production, and spare capacity—which was restored upon treatment (Figures 7G-J). Concurrently, CTCF elevated glycolytic flux (ECAR), glycolysis-derived ATP, and lactate levels, all of which were reversed by intervention (Figures 7K-N).

Beyond fibroblasts, stromal remodeling also involves TAM activation, which can be driven by NAMPT signaling 57. This stimulation promotes a phenotypic switch, increasing M2-associated markers (arginase-1, CD163, IL-10) 58 while suppressing M1 markers (iNOS, TNF-α) 59. Herein, we demonstrated in vivo that CTCF up-regulation in HL60-derived TANs enhanced M2 macrophage activation (Figures 7O-P). Spatial analysis of 30 CRC samples via HALO™ revealed that in regions with high neutrophil infiltration, CTCF+ TANs were closely associated with CAFs (FAP+) and TAMs (SPP1+), a pattern absent in low-infiltration regions (Figures 7Q-R). Proximity histograms (Figure 7S) further confirmed significant clustering of CAFs and TAMs within 50 μm of CTCF+ TANs, underscoring Neu2's role in promoting stromal cell activation and aggregation in CRC.

### Neu2 maintains an immunosuppressive phenotype

**Figure 8:**
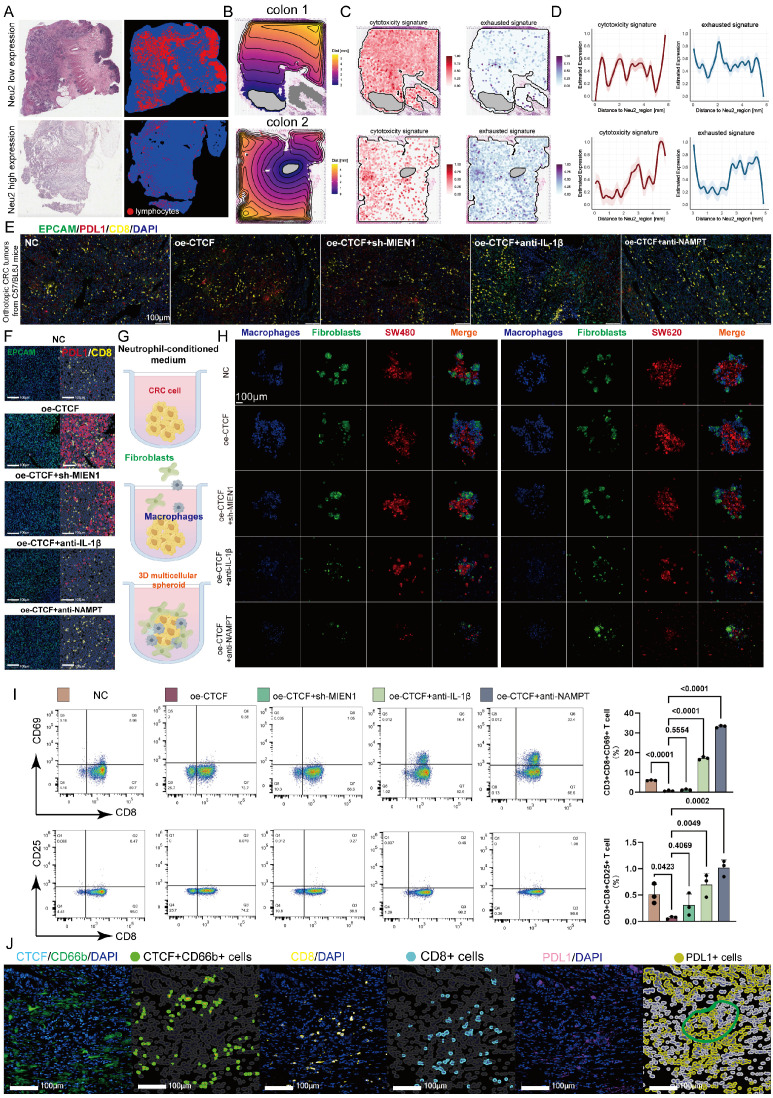
**Neu2 induces immune exclusion and dysfunction in colorectal cancer. (A)** HE staining and lymphocyte distribution in colorectal cancer tissues with low and high Neu2 neutrophil expression (TCGA-CRC dataset). Left row: Representative HE-stained section from a sample with low Neu2 expression. Adjacent panels show lymphocyte distribution (red) overlaid on tissue architecture (blue). Right row: Samples with high Neu2 expression display fewer lymphocytes across tissue regions. **(B)** Spatial contour maps of tissue distance from Neu2-enriched regions in colorectal cancer samples. Representative samples (colon 1 and colon 2) illustrate spatial proximity to Neu2-enriched areas. Distance is measured in millimeters, with purple indicating closer proximity and yellow representing farther distances. Gray regions represent Neu2-enriched zones with the highest expression levels.** (C)** Expression patterns of cytotoxicity and exhaustion signatures. Spatial transcriptomics data illustrate the distribution of cytotoxicity (left) and exhaustion (right) gene signatures across tissue sections.** (D)** Distance-dependent expression of T cell signatures relative to Neu2-enriched regions. Line plots display cytotoxicity (left) and exhaustion (right) signature expression as a function of distance from Neu2-enriched regions. **(E)** Immunofluorescence staining of orthotopic CRC tumors from C57BL/6 mice. Tumor sections were stained for EPCAM (green, marking epithelial cancer cells), PDL1 (red, indicating immune checkpoint expression), CD8 (yellow, marking cytotoxic T cells), and DAPI (blue, for nuclei). NC (negative control) tumors exhibit sparse CD8+ T cell infiltration and low PDL1 expression. In contrast, oe-CTCF tumors show increased spatial proximity of CD8+ T cells to PDL1+ regions, suggesting enhanced immune suppression. Intervention groups (oe-CTCF + sh-MIEN1 and oe-CTCF + anti-IL-1β) show reduced CD8/PDL1 co-localization. Scale bar: 100 μm.** (F)** HALO platform-based analysis of spatial proximity between CD8+ T cells and PDL1 expression within EPCAM-positive tumor regions. Immunofluorescence staining highlights EPCAM (green) as a marker of epithelial cancer cells, CD8 (yellow) representing cytotoxic T cells, and PDL1 (red) as an immune checkpoint molecule. The oe-CTCF group shows a marked increase in CD8/PDL1 physical proximity, reflecting enhanced immune suppression within tumor regions. Interventions such as oe-CTCF + sh-MIEN1, oe-CTCF + anti-IL-1β, and oe-CTCF + anti-NAMPT reduce this colocalization. Scale bar: 100 μm.** (G)** Schematic of the experimental setup for studying neutrophil-conditioned medium (NCM) effects on CRC cells and the tumor microenvironment.** (H)** Representative images of 3D multicellular spheroids comprising macrophages (blue), fibroblasts (green), and colorectal cancer (CRC) cells (red: SW480, SW620) under different conditioned medium (NCM) treatments. CM was derived from co-cultures of HL60-derived neutrophils under different conditions. Spheroids treated with oe-CTCF CM show increased clustering of macrophages and fibroblasts around CRC cells, suggesting enhanced stromal recruitment and tumor-stroma interactions. oe-CTCF + anti-NAMPT showing the most pronounced reduction.** (I)** Flow cytometry analysis of CD8+ T cells expressing activation markers CD69 and CD25 stimulated by CM from 3D multicellular spheroids. Neutrophils were treated under different conditions. CM from oe-CTCF-treated co-cultures significantly increased CD69+ and CD25+ T cell proportions compared to NC (P < 0.0001). Interventions (anti-IL-1β, anti-NAMPT) reduced this effect, with anti-NAMPT showing the strongest inhibition.** (J)** Using the HALO platform for AI-based spatial analysis, mIF staining reveals that CTCF+ neutrophils (green) promote direct physical proximity between CD8+ T cells (cyan) and PD-L1+ cells (yellow) in colorectal cancer tissues. This spatial clustering suggests that CTCF+ neutrophils facilitate PD-L1-mediated CD8+ T cell exhaustion, contributing to immune suppression. Scale bars: 100 μm.

TANs contribute to CD8+ T cell exhaustion in the TME by secreting immunosuppressive factors and interacting with T cells 60, 61. TCGA-CRC samples revealed that low Neu2 levels correlated with higher CD8+ T cell infiltration (Figure 8A). We analyzed the spatial relationship between Neu2 expression and T cell features, noting distinct expression gradients of cytotoxic and exhausted signatures associated with Neu2 in CRC samples (Figures 8B-D, S15A-C), suggesting Neu2's role in T cell function. Given the well-known roles of PD-1/PD-L1 interactions in T cell exhaustion and immune suppression in the TME 62, we performed in mIF staining to assess the effect of Neu2 on PD-L1 expression. Further investigation into PD-L1 expression, a key regulator of T cell exhaustion, showed that CTCF-MIEN1-IL1B signaling enhanced PD-L1 expression and promoted spatial proximity between PD-L1 and CD8+ T cells (Figure 8E). These results indicate that Neu2 may contribute to T cell exhaustion (Figure 8F). Thus, we further hypothesized that Neu2 facilitates immune evasion by impairing T cell migration and function. To test this, we performed co-culture experiments with macrophages, fibroblasts, and CRC cells using neutrophil-conditioned media (Figure 8G). CTCF over-expression enhanced encapsulation of CRC cells by fibroblasts and macrophages, an effect attenuated by sh-MIEN1, anti-IL-1β, or anti-NAMPT treatment (Figure 8H). This suggested that Neu2 may contribute to T cell exclusion from the TME. Flow cytometry confirmed that CTCF over-expression suppressed CD8+ T cell activation, as indicated by reduced CD69+ and CD25+ expression, which was partially rescued by the above interventions (Figure 8I). Additionally, mIF of clinical samples further revealed that PD-L1+ cells clustered around CD8+ T cells in regions enriched with CTCF+ TANs, confirming spatial coordination that may contribute to immune suppression (Figure 8J).

### Neu2 is a deleterious factor for immunotherapy

**Figure 9:**
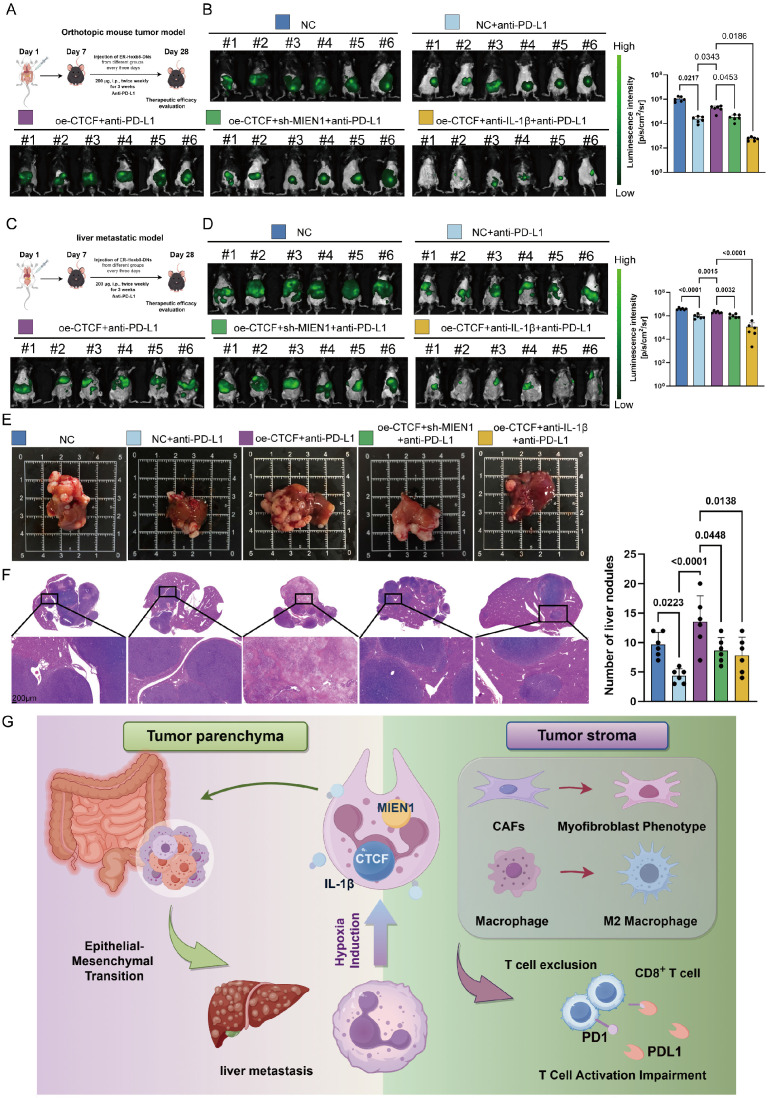
** Neu2 impairs the efficacy of anti-PD-L1 therapy in CRC. (A)** Schematic representation of the orthotopic colorectal cancer model and treatment strategy. mc38-luc cells were injected into the cecal wall to establish orthotopic tumors. Beginning one-week post-injection, mouse ER-Hoxb8-DNs neutrophils were administered intraperitoneally every three days, while anti-PD-L1 (200 µg/mouse) was given twice weekly. **(B)** Representative bioluminescence images of orthotopic CRC tumors. Quantification of luminescence intensity shows significantly increased tumor burden in the oe-CTCF group, while MIEN1 knockdown (oe-CTCF + sh-MIEN1) or IL-1β neutralization (oe-CTCF + anti-IL-1β) restores the efficacy of anti-PD-L1 therapy.** (C)** Schematic representation of the liver metastasis treatment model. Liver metastases were established by splenic vein injection of mc38-luc cells. Mice received intraperitoneal injections of mouse ER-Hoxb8-DNs neutrophils from different experimental groups every three days, along with twice weekly anti-PD-L1 therapy.** (D)** Representative bioluminescence images of liver metastases. Quantification of luminescence intensity reveals that oe-CTCF neutrophils impair anti-PD-L1 efficacy, while MIEN1 knockdown or IL-1β neutralization improves treatment response.** (E)** Gross images of liver tissues from liver metastasis models. Visible liver metastases are larger and more numerous in the oe-CTCF group, while MIEN1 knockdown or IL-1β neutralization reduces metastatic burden.** (F)** Representative HE-stained liver sections showing metastatic nodules. Quantitative analysis of nodule counts demonstrates a significant reduction in the oe-CTCF + sh-MIEN1 and oe-CTCF + anti-IL-1β groups compared to the oe-CTCF group. Scale bar: 200 µm. Statistical significance was assessed using one-way ANOVA followed by Tukey's post hoc test. P values are indicated for each comparison (mean ± SD).** (G)** Schematic summary of the proposed mechanism involving Neu2 and its role in CRC progression.

In many circumstances, TAN accumulation and diversity in tumors can negatively interfere with immune checkpoint inhibitors (ICIs) 63. Now that we have demonstrated the potent immunosuppressive function of Neu2, the effect of CTCF-MIEN1-IL-1β signaling on anti-PD-L1 therapy in CRC was investigated, both in primary tumors and liver metastases. Silencing CTCF in ER-Hoxb8-DNs combined with anti-PD-L1 treatment, as well as anti-Ly6G combined with anti-PD-L1 treatment, both showed significant therapeutic enhancement (Figure S16). Furthermore, we found that up-regulation of CTCF in ER-Hoxb8-DNs resulted in impaired anti-PD-L1 treatment efficacy, which was partially rescued by sh-MIEN1 or anti-IL-1β treatment (Figures 9A-E). Quantitative analysis of Hematoxylin/eosin (HE) sections further supported these findings (Figure 9F). Our results suggest that Neu2 may regulate immune checkpoint therapy in CRC and is detrimental to immunotherapy. Importantly, reduction of Neu2 or TANs would enhance the therapeutic potential of anti-PD-L1.

## Discussion

TANs play pivotal roles in cancer initiation and progression, exhibiting remarkable functional plasticity within the TME 64. Through interactions with cancer and immune cells, TANs regulate key processes such as angiogenesis, inflammation, and immune suppression 65. TAN polarization into antitumorigenic (N1-like) or tumorigenic (N2-like) subsets significantly influences tumor behavior, including proliferation, invasion, and metastasis 66. In this study, we identified a distinct TAN subset, termed Neu2, associated with colorectal malignant transformation and marked by elevated CTCF-MIEN1-IL-1β signaling (Figure 9G). Notably, Neu2 recruitment to tumor cells was largely dependent on MIEN1, a gene best known for its role in promoting cancer cell motility and invasion 67, 68. While MIEN1 has been studied extensively in tumor biology, its involvement in neutrophil chemotaxis has been poorly characterized. Our findings reveal a novel, non-inflammatory MIEN1-dependent mechanism by which TANs are drawn toward tumor cells—potentially allowing immune cell infiltration without triggering tumor-damaging inflammatory responses. Beyond chemotaxis, Neu2 is also characterized by IL-1β secretion, a process likely independent of MIEN1 but potentially regulated by CTCF. Transcriptomic profiling following CTCF overexpression identified HOXA13 as a compelling candidate mediator of IL-1β expression. HOXA13 has been implicated in promoting IL-1β secretion and cytoskeletal remodeling in other cell types 69, and CTCF has been shown to dysregulate HOX gene transcription, including HOXA13, particularly in hematologic malignancies 70. This suggests a possible transcriptional axis in which CTCF upregulates IL-1β via HOXA13. In addition, emerging evidence links CTCF to the caspase-1/IL-1β axis. For instance, CTCF has been implicated in regulating caspase-1 activity through upstream effectors such as DPP4, thereby influencing IL-1β maturation 39, 71, Together, these findings suggest that CTCF may regulate IL-1β at both transcriptional and post-transcriptional levels. IL-1β itself is a pleiotropic cytokine with well-established roles in shaping an immunosuppressive TME. It promotes the recruitment and activation of suppressive immune populations 72, 73, while simultaneously enhancing the invasive and metastatic capacity of tumor cells 74. Thus, Neu2 may represent a multifaceted paradigm in tumor biology.

Neu2 exhibits heterogeneous distribution in CRC, with a predominant localization in hypoxic tumor regions. Hypoxia is a well-established modulator of CTCF expression and function 40, altering chromatin accessibility and disrupting topologically associated domain (TAD) boundaries through changes in DNA-binding affinity 75. This spatial association highlights the contribution of the hypoxic microenvironment to CRC progression.

TANs frequently infiltrating the TME, exhibit complex interactions with cancer cells, particularly in the context of EMT 76. EMT facilitates tumor invasion and metastasis by enabling epithelial cancer cells to acquire mesenchymal traits 77. TANs have been shown to influence EMT through cytokine and chemokine signaling, with their impact varying by polarization state (N1-like vs. N2-like) 78. In our study, Neu2 was found to promote EMT, specifically the partial EMT (pEMT) program, which confers hybrid epithelial-mesenchymal properties and is closely linked to increased tumor plasticity, invasiveness, and resistance to therapy 79, 80. pEMT is regulated by signaling pathways such as TGF-β, Wnt, and Notch 81, and we validated its induction by Neu2 through both in vivo assays and ST analysis. Importantly, Neu2 presence was associated with enhanced liver metastasis.

Tumor progression is often accompanied by increased stromal infiltration, including fibroblasts, immune cells, and endothelial cells 82, which collectively remodel the TME to support tumor growth, angiogenesis, and immune evasion 83, 84. Neu2 appears to be a key driver of this process, stimulating the activation of CAFs and inducing M2-like polarization of TAMs. These cell types often cooperate to construct an immunosuppressive niche and facilitate tumor progression 85. Neu2 may thus reinforce this crosstalk, further sustaining the TME's tumor-supportive functions.

Functionally, Neu2 also contributes to immune exclusion and T cell dysfunction. We observed that Neu2 presence was associated with T cell exclusion and exhaustion, likely due to the establishment of a physical stromal barrier and the secretion of immunosuppressive factors. Such an environment hampers effector T cell activity and undermines the efficacy of immunotherapy 86, 87. Therefore, Neu2 infiltration may represent a previously underappreciated mechanism of immunotherapy resistance in CRC.

Multiple TAN subsets have been described in other cancers, each contributing uniquely to hypoxia adaptation, EMT induction, immune suppression, and therapy resistance 88, 89. CD71+ TANs in glioblastoma promote immunosuppression via lactate-driven histone lactylation and ARG1 upregulation 90; T3-TANs (marked by dcTRAIL-R1) in pancreatic cancer remodel vasculature through VEGFα secretion and accelerate tumor growth 91; CD10+ALPL+ TANs in hepatocellular carcinoma induce CD8+ T-cell exhaustion via NAMPT signaling 92; and RLSNs (Retnlg+Lcn2+ senescence-like neutrophils) in bladder cancer, impaired in ferroptosis, create a metastasis-promoting niche 93. Additionally, neutrophil extracellular traps (NETs), triggered by mitochondrial respiration-induced hypoxia, form feedforward loops that facilitate cancer dissemination 94, 95. While Neu2 shares many functional similarities with these TAN populations—such as localization to hypoxic regions and promotion of immunosuppression—it exhibits a unique feature of non-inflammatory chemotaxis, which may reflect a novel adaptive mechanism within the TME.

To enhance translational relevance of Neu2 research, future studies should prioritize in vivo validation using patient-derived xenograft models to confirm CTCF/MIEN1 axis targeting efficacy. Development of Neu2-specific functional assays, including high-dimensional flow cytometry panels and spatial transcriptomics, would enable precise quantification of its immunosuppressive capacity. Clinical translation could be accelerated through biomarker-guided cohort stratification in immunotherapy trials, coupled with exploration of small-molecule inhibitors targeting hypoxia-induced CTCF stabilization to disrupt Neu2-mediated tumor progression.

Despite the promising avenues explored in this study, several limitations merit consideration. Firstly, the clinical cohort employed in our analysis is characterized by a relatively small sample size, which may limit the generalizability of our findings and increase the potential for bias. This constraint necessitates caution in interpreting the statistical significance and clinical relevance of our observations. We are collecting patient data in multicenter clinical cohorts for further analysis and validation. Although some immunohistochemistry and fluorescence experiments have been completed, in-depth analysis of large-scale protein sequencing is still needed. Secondly, the precise regulatory mechanism underlying CTCF's modulation of IL-1β remains elusive, posing some unavoidable obstacles to fully understanding the biological pathways implicated in our study. Lastly, technical limitations imposed by the computational resources available to us have restricted our capacity to perform high-resolution spatial transcriptomic analyses of the CRC microenvironment. Consequently, subtle spatial heterogeneities and complex interactions within the TME, which could offer invaluable insights into CRC progression and response to treatment, remain unresolved. Addressing these limitations through larger sample sizes, deeper mechanistic investigations, and advanced computational capabilities will be crucial for advancing our understanding and treatment strategies for CRC.

## Conclusions

This study identifies tissue-resident tumor-associated neutrophils (Neu2) as a critical driver of CRC progression and immune evasion. Neu2, characterized by CTCF-MIEN1-IL-1β signaling, promotes EMT, enhances tumor invasion, and fosters an immunosuppressive TME. These TANs, enriched in hypoxic tumor regions, interact with stromal cells and macrophages to amplify metabolic and immunological reprogramming, contributing to tumor growth and resistance to immunotherapy. Our findings position Neu2 as a key player in CRC aggressiveness and as a major factor limiting the efficacy of immune checkpoint blockade therapies. Targeting tissue-resident Neu2 TANs could serve as a novel therapeutic strategy to overcome immunotherapy resistance.

## Supplementary Material

Supplementary figures, methods and tables.

## Abbreviations

CRC: Colorectal cancer
TANs: Tumor-associated neutrophils
CTCF: CCCTC-Binding Factor
TME: Tumor microenvironment
ST: Spatial transcriptome
scRNA-seq: Single-cell RNA sequencing
MIEN1: Migration and Invasion Enhancer 1
CRLM: CRC liver metastasis
mIF: Multicolor immunofluorescence
EMT: Epithelial-to-Mesenchymal Transition
pEMT: partial EMT
cEMT: complete EMT
TAMs: Tumor-associated macrophages
CAFs: Cancer-associated fibroblasts
NAMPT: Nicotinamide phosphoribosyl transferase
ICIs: Checkpoint inhibitors
