# Supplementary material for: Comprehensive analytical approach identifies a subtype of CTCF+ tumor-associated neutrophils associated with CRC development and as a biomarker for immunotherapy
Source: Int J Biol Sci. 2025 Aug 16;21(12):5305–27. doi: 10.7150/ijbs.111529 (PMC12435489; doi:10.7150/ijbs.111529)
Supplement: Supplementary file 1 — Supplementary figures, methods and tables. [file ijbsv21p5305s1.pdf]

Supplementary Material

Supplementary Table

Table S1

Antibodies and Reagents	Manufacturer, Country, Cat number, Lot number	Concentration
Roswell Park Memorial Institute 1640 (RPMI-1640)	Gibco, USA, 31870082, 8123063	-
Dulbecco's modified Eagle's medium (DMEM)	Gibco, USA, C11995500BT, 8122778	-
Phosphate buffered saline (PBS)	Gibco, USA, 70011-044, 8123148	-
Fetal bovine serum (FBS)	Gibco, USA, 10099-141, 2441561RP	-
Collagenase III	Biosharp, China, BS164-100mg, B0013K030100	-
Trypsin	Gibco, USA, 25300-054, 2509042	-
Matrigel	Corning, USA, 356234, 10124002	-
Puromycin	Beyotime Biotechnology, China，ST551-10mg, 050823230613	-
4% Paraformaldehyde	Biosharp, China, BL539A,23159313	-
Electron microscope fixative	Servicebio, China, G1102-1.5ML, CR2208118	-
Mitochondrial Membrane Potential Assay Kit with JC-1	Solarbio life sciences, China, M8650,2307001	-
MitoTracker Red	Solarbio life sciences, China, M9940,2311006	-
Rhodamine phalloidin	Solarbio life sciences, China, CA1610, 20240704	-
Luciferase Assay Kit	Promega, USA, N1610, XI358119	-
Seahorse XF Cell Mito Stress Test Kit	Agilent Seahorse Bioscience, USA, 103015-100, 17601020	-
Seahorse XF Glycolytic Rate Assay Kit	Agilent Seahorse Bioscience, USA, 103344-100,17592084	-
DiR iodide (DilC(7)) deep red fluorescent probe	YEASEN, China, 40757ES25, D3411220	-
CellTracker Blue CMAC (7-amino-4chloromethylcoumarin)	Thermo Fisher Scientific, USA, C2110, 3112785	-
4',6-diamidino-2-phenylindole (DAPI)	Beyotime Biotechnology, China, C1002, 091620210520	-
Immunastaining Permeabilization	Beyotime Biotechnology, China, P0096-	-

Buffer with TritonX-100	100ml,042921211027	
Protease and phosphatase inhibitor cocktail for genneraluse,50X	Beyotime Biotechnology, China, P1045,051823230618	-
RBC Lysis Buffer (10×)	Biosharp, China, CS003,220903	-
bovine serum albumin (BSA)	Vazyme, USA, B2270DBA,027E2270DA	-
Deoxyribonuclease I	Beyotime Biotechnology, China, D7073, 112522230619	-
Crystal violet	Beyotime Biotechnology, China, C0121-100ml,121322230524	-
Radioimmunoprecipitation assay buffer (RIPA buffer)	Beyotime Biotechnology,China , P0013B,052523230703	-
Tricolor Prestained Protein Marker	EpiZyme, China, WJ106,027352000	-
Dimethyl sufoxide (DMSO)	Sigma-Aldrich, USA, D2650-100, RNBM2943	-
phorbol 12-myristate 13-acetate (PMA)	Sigma-Aldrich, USA, Lot: SLBX8899	100 nM
Recombinant Human EGF Animal-Free manufactured	Peprotech, USA, AF-100-15, 111908	20 ng/ml
Recombinant Human FGF-basic (154 a.a.)	Peprotech, USA, 100-18B-100, 0820AFC05	20 ng/ml
Recombinant Human IL-1beta	Peprotech, USA, 200-01B-2UG, 0606B95-1	0-40 ng/ml
InVivoMab anti-mouse/rat IL-1β, Clone: B122, Size: 1 mg	Lebanon, USA, BE0243, 50-562-789 Clone: ALF-161	1.0 mg/kg, i.p., twice weekly
InVivoMAb anti-mouse PD-L1	Lebanon, USA, BE0101, 50-562-316, 10F.9G2	200 μg, i.p., twice weekly for 3 weeks
Daporinad (Synonyms: FK866; APO866), anti-NAMPT	MCE, China, HY-50876,09317	Mouse: 20mg/kg, i.p., once daily Cell: 5 μM
Raleukin (Synonyms: AMG-719; Anakinra), anti- IL-1β	MCE, China, HY-108841, 227797	10 μg/ml
Isoflurane	Ruiwode Lifescience, China, R510-22-10, 2024082201	2%
D-Luciferin Potassium Salt	BLT Photon Tech, China, LS003, 92410493154	100 mg/kg
B27 supplement	Gibco, USA, 17504044, 2814927	2%
Calcein-AM Solution	Solarbio life sciences, China, CA1630-1, 240005001	-
CD8 MicroBeads	Miltenyi Biotec, Bergisch Gladbach, Germany, 130-045-201, 5240902334	-
LS Columns	Miltenyi Biotec, Bergisch Gladbach,	-

	Germany, 130-042-401, 9240223460	
EasySep^{TM} Human Neutrophil Enrichment Kit	STEMCELL Technologies, Canada, 17957, 1000140311	-
Cell Counting Kit-8 (CCK-8)	APExBIO, USA, K1018-5ml, K10182533EF5E	-
Lipofectamine 3000	Invitrogen, USA, L3000-015, 2455275	-
Protein Quantification Kit (BCA Assay)	Abbkine, China, KTD3001, ATXC07081	-
Tyramide signal amplification biotin system kit	UElady Biotechnology, China, Y6082L,210413L2-1	-
12% SurePAGE,Bis-Tris	GenScript, China, M00669, C35352408	-
10% SurePAGE,Bis-Tris	GenScript, China, M00666, C35652407	-
Tris-MOPS-SDS Running Buffer	GenScript, China, M00138, C31382407	-
Hematoxylin-Eosin staining Kit	Solarbio life sciences, China, G1120, 20230913	-
Human NAMPT ELISA Kit	Shanghai Enzyme Linked Biology, China, ml060212, 05/2024	-
Human IL-1β ELISA Kit	Shanghai Enzyme Linked Biology, China, ml058059, 05/2024	-
Anti-CD66b Rabbit monoclonal antibody	Abcam, England, ab300122, 1067934-23	IF 1:100
IL1 beta Antibody	Affinity Biosciences, China, AF5103, 83h9328	WB 1:1000<br>IF 1:100
CTCF Rabbit mAb	Cell Signaling Technology, USA, 3418, 6	WB 1:1000<br>IF 1:100
XTP4 (MIEN1) Monoclonal Antibody	Thermo Fisher Scientific, USA, MA5-26355, 3ADD8A32	WB 1:1000<br>IF 1:100
NLRP3 Monoclonal antibody	Proteintech, USA, 68102-1-Ig, 10040218	WB 1:1000
Caspase-1 Antibody	Cell Signaling Technology, USA, 2225,4	WB 1:1000
Cleaved-Caspase-1 Rabbit mAb	Cell Signaling Technology, USA, 4199T, 6	WB 1:1000
Pro-IL-1β Rabbit mAb	Cell Signaling Technology, USA, 83186T, 2	WB 1:1000
Anti-HIF-1 alpha antibody	Abcam, England, ab113642, GR116444-2	IF: 1:100
Fas Rabbit mAb	Cell Signaling Technology, USA, 4233T, 3	WB 1:1000
TNF-α Rabbit mAb	Cell Signaling Technology, USA, 6945,12	WB 1:1000
Arginase-1 Rabbit mAb	Cell Signaling Technology, USA, 93668T, 4	WB 1:1000
VEGFA Monoclonal Antibody	Thermo Fisher Scientific, USA, MA5-13182, ZL4574921	WB 1:1000

MCP-1 (CCL2) Monoclonal Antibody	Thermo Fisher Scientific, USA, MA5-17040, ZL4560801	WB 1:1000
ICAM1 Rabbit monoclonal antibody	Abcam, England, ab109361, 1083413-2	WB 1:1000
Anti-E-cadherin Rabbit polyclonal Antibody	Cell Signaling Technology, USA, 3195, 6	WB 1:1000
TNC/Tenascin-C Monoclonal antibody	Proteintech, USA, 67710-1-Ig, 10011696	IF: 1:100<br>WB: 1:1000
Anti N-Cadherin Antibody	Abcam, England, ab76011, 1000650-16	WB 1:1000
Anti-MMP2 Rabbit polyclonal Antibody	Cell Signaling Technology, USA, 40994, 3	WB 1:1000
Anti-MMP9 Rabbit polyclonal Antibody	Cell Signaling Technology, USA, 13667, 5	WB 1:1000
Anti-β-actin Mouse polyclonal Antibody	Proteintech, USA, 66009-1-Ig, 10038080	WB 1:5000
Anti-GAPDH Mouse polyclonal Antibody	Proteintech, USA, 60004-1-Ig, 10029187	WB 1:3000
Anti-β-Tubulin (C66) mAb	Abmart. China, M20005S, 10117717	WB 1:5000
EpCAM Monoclonal Antibody	Thermo Fisher Scientific, USA, MA5-12436, ZG396593	IF 1:200
Anti-CD8 alpha antibody	Abcam, England, ab217344, 1006843-42	IF 1:200
PD-L1 Rabbit Polyclonal Antibodies	Abcam, England, ab205921,00052042	IF 1:200
PD-L1/CD274 Monoclonal antibody	Proteintech, USA, 66248-1-Ig,10031805	IF 1:200
α-Smooth Muscle Actin (D4K9N)	Cell Signaling Technology, USA, 19245S, 3	IF: 1:320
FAP (E1V9V) Rabbit mAb	Cell Signaling Technology, USA, 66562, 5	IF: 1:100
Anti-GJA4 Rabbit polyclonal Antibody	Abmart, China, TP72318, 10145898	WB 1:1000
Collagen Type I Polyclonal antibody	Proteibtech, USA, 14695-1-AP, 00105556	WB 1:1000
Anti-CD163 Rabbit Polyclonal Antibody	Proteintech, USA, 16646-1-AP, 20001029	IF 1:200
Osteopontin (SPP1) Polyclonal antibody	Proteintech, USA, 22952-1-AP, 00138205	IF 1:200
Highly Cross-Adsorbed Goat (Polyclonal) Anti-Mouse IgG(H+L) Antibody	LI-COR, USA, 926-68070, Q04695	WB 1:5000
Highly Cross-Adsorbed Goat (Polyclonal) Anti-Rabbit IgG(H+L) Antibody	LI-COR, USA, 926-68071, S11385	WB 1:5000

APC anti-human CD69	BioLegend, USA, 310910, B427120, clone: FN50	5ul/1×10^6 Cell
Brilliant Violet 421™ anti-human CD25	BioLegend, USA, 302630, B393495, clone: BC96	5ul/1×10^6 Cell
Alexa Fluor® 488 anti-human CD3	BioLegend, USA, 317310, B369206	5ul/1×10^6 Cell
PE anti-human CD8	BioLegend, USA, 317310, 344706, clone:SK1	5ul/1×10^6 Cell
APC/Fire™ 750 anti-human CD45	BioLegend, USA, 304062, B402221, clone: HI30	5ul/1×10^6 Cell
Alexa Fluor® 647 anti-human CD66b	BioLegend, USA, 392912, B374318, clone:6/40	5ul/1×10^6 Cell
Brilliant Violet 421™ anti-human CD16	BioLegend, USA, 302038, B397014, Clone:3G8	5ul/1×10^6 Cell
APC anti-human CD69	BioLegend, USA, 310910, B427120, clone: FN50	5ul/1×10^6 Cell

Table S2

Gene information

Gene symbol	GenBank_ID	species
MIEN1	NM_032339.5	Human

Table S3

Target information

NO.	Accession	Target Seq	CDS	GC%
MIEN1-RNAi(136727-1)	NM_032339.5	GGGCTTTCCCTATGAGAAAGA	38..385	47.62%
MIEN1-RNAi(136728-2)	NM_032339.5	GGTGTTCTCCAAGCTGGAGAA	38..385	52.38%

MIEN1-RNAi(136729-1)	NM_032339.5	GCACAGGTGCCTTTGAGATAG	38..385	52.38%

Plasmid name：GV493

Negative control insert sequence：TTCTCCGAACGTGTCACGT

Order of the vector elements: hU6-MCS-CMV-Puromycin

Table S4

Synthetic oligo information

NO.	5’	STEM	Loop	STEM	3’
MIEN1-RNAi(136727-1)-a	ccgg	GGGCTTTCCCTATGAGAAAGA	CTCGAG	TCTTTCTCATAGGGAAAGCCC	TTTTTg
MIEN1-RNAi(136727-1)-b	aattcaaaaa	GGGCTTTCCCTATGAGAAAGA	CTCGAG	TCTTTCTCATAGGGAAAGCCC	
MIEN1-RNAi(136728-2)-a	ccgg	GGTGTTCTCCAAGCTGGAGAA	CTCGAG	TTCTCCAGCTTGGAGAACACC	TTTTTg
MIEN1-RNAi(136728-2)-b	aattcaaaaa	GGTGTTCTCCAAGCTGGAGAA	CTCGAG	TTCTCCAGCTTGGAGAACACC	
MIEN1-RNAi(136729-1)-a	ccgg	GCACAGGTGCCTTTGAGATAG	CTCGAG	CTATCTCAAAGGCACCTGTGC	TTTTTg
MIEN1-RNAi(136729-1)-b	aattcaaaaa	GCACAGGTGCCTTTGAGATAG	CTCGAG	CTATCTCAAAGGCACCTGTGC	

Table S5

Over-expression Plasmid information

**ID**	**seq**
MIEN1-P1	CCAACTTTGTGCCAACCGGTCGCCACCATGAGCGGGGAGCCGGGGCA
MIEN1-P2	CACACATTCCACAGGAATTTCACAGGATGACGCAGGGAG

Plasmid name： GV348

Order of the vector elements: Ubc-MCS-SV40-puromycin

The positive clones sequencing results were analyzed

The comparison results were shown as follows：

TTTTTTGTTAGACGAAGCTTGGGCTGCAGGTCGACTCTAGAGGATCCAACTTTGTGCCAACCGGTCGCCACCATGAGCGGGGAGCCGGGGCAGACGTCCGTAGCGCCCCCTCCCGAGGAGGTCGAGCCGGGCAGTGGGGTCCGCATCGTGGTGGAGTACTGTGAACCCTGCGGCTTCGAGGCGACCTACCTGGAGCTGGCCAGTGCTGTGAAGGAGCAGTATCCGGGCATCGAGATCGAGTCGCGCCTCGGGGGCACAGGTGCCTTTGAGATAGAGATAAATGGACAGCTGGTGTTCTCCAAGCTGGAGAATGGGGGCTTTCCCTATGAGAAAGATCTCATTGAGGCCATCCGAAGAGCCAGTAATGGAGAAACCCTAGAAAAGATCACCAACAGCCGTCCTCCCTGCGTCATCCTGTGAAATTCCTGTGGAATGTGTGTCAGTTAGGGTGTGGAAAGTCCCCAGGCTCCCCAGCAGGCAGAAGTATGCAAAGCATGCATCTCAATTAGTCAGCAACCAGGTGTGGAAAGTCCCCAGGCTCCCCAGCAGGCAGAAGTATGCAAAGCATGCATCTCAATTAGTCAGCAACCATAGTCCCGCCCCTAACTCCGCCCATCCCGCCCCTAACTCCGCCCAGTTCCGCCCATTCTCCGCCCCATGGCTGACTAATTTTTTTTATTTATGCAGAGGCCGAGGCCGCCTCTGCCTCTGAGCTA

Table S6

Gene information

Gene symbol	GenBank_ID	species
CTCF	NM_006565.4	Human

Table S7

Target information

NO.	Accession	Target Seq	CDS	GC%
CTCF-RNAi(136724-1)	NM_006565.4	TGGCAAGACATGCTGATAATT	326..2509	38.10%
CTCF-RNAi(136725-1)	NM_006565.4	TTGCGAAAGCAGCATTCCTAT	326..2509	42.86%
CTCF-RNAi(136726-2)	NM_006565.4	GGCACATGATCATGCACAAGC	326..2509	52.38%

Plasmid name：GV152

Negative control insert sequence：TTCTCCGAACGTGTCACGT

Order of the vector elements: hU6-MCS-CMV-Neomycin

Table S8

Synthetic oligo information

NO.	5’	STEM	Loop	STEM	3’
CTCF-RNAi(136724-1)-a	Ccgg	TGGCAAGACATGCTGATAATT	CTCGAG	AATTATCAGCATGTCTTGCCA	TTTTTg
CTCF-RNAi(136724-1)-b	aattcaaaaa	TGGCAAGACATGCTGATAATT	CTCGAG	AATTATCAGCATGTCTTGCCA	
CTCF-	Ccgg	TTGCGAAAGCAGCATT	CTC	ATAGGAATGCTGCT	TTTTTg

RNAi(136725-1)-a		CCTAT	GAG	TTCGCAA	
CTCF-RNAi(136725-1)-b	aattcaaaaa	TTGCGAAAGCAGCATTCCTAT	CTCGAG	ATAGGAATGCTGCTTTCGCAA	
CTCF-RNAi(136726-2)-a	Ccgg	GGCACATGATCATGCACAAGC	CTCGAG	GCTTGTGCATGATCATGTGCC	TTTTTg
CTCF-RNAi(136726-2)-b	aattcaaaaa	GGCACATGATCATGCACAAGC	CTCGAG	GCTTGTGCATGATCATGTGCC	

Table S9

Over-expression Plasmid information

**ID**	**seq**
CTCF-P1	GAGGATCCCCGGGTACCGGTCGCCACCatggaaggtgatgcagtcgaag
CTCF-P2	CACACATTCCACAGGCTAGCtcaccggtccatcatgctgaggatc

Plasmid name：CV084

Order of the vector elements: Ubc-MCS-SV40-Neomycin

The positive clones sequencing results were analyzed

The comparison results were shown as follows :

GTCGACTCTAGAGGATCCCCGGGTACCGGTCGCCACCATGGAAGGTGATGCAGTCGAA
GCCATTGTGGAGGAGTCCGAAACTTTTATTAAAGGAAAGGAGAGAAAGACTTACCAG
AGACGCCGGGAAGGGGGCCAGGAAGAAGATGCCTGCCACTTACCCCAGAACCAGAC
GGATGGGGGTGAGGTGGTCCAGGATGTCAACAGCAGTGTACAGATGGTGATGATGGA
ACAGCTGGACCCCACCCTTCTTCAGATGAAGACTGAAGTAATGGAGGGCACAGTGGCT
CCAGAAGCAGAGGCTGCTGTGGACGATACCCAGATTATAACTTTACAGGTTGTAAATAT
GGAGGAACAGCCCATAAACATAGGAGAACTTCAGCTTGTTCAAGTACCTGTTCCTGTG
ACTGTACCTGTTGCTACCACTTCAGTAGAAGAACTTCAGGGGGCTTATGAAAATGAAG

TGTCTAAAGAGGGCCTTGCGGAAAGTGAACCCATGATATGCCACACCCTACCTTTGCCT
GAAGGGTTTCAGGTGGTTAAAGTGGGGGCCAATGGAGAGGTGGAGACACTAGAACAA
GGGGAACTTCCACCCCAGGAAGATCCTAGTTGGCAAAAAGACCCAGACTATCAGCCA
CCAGCCAAAAAAACAAAGAAAACCAAAAAGAGCAAACTGCGTTATACAGAGGAGGG
CAAAGATGTAGATGTGTCTGTCTACGATTTTGAGGAAGAACAGCAGGAGGGTCTGCTA
TCAGAGGTTAATGCAGAGAAAGTGGTTGGTAATATGAAGCCTCCAAAGCCAACAAAA
ATTAAAAAGAAAGGTGTAAAGAAGACATTCCAGTGTGAGCTTTGCAGTTACACGTGTC
CACGGCGTTCAAATTTGGATCGTCACATGAAAAGCCACACTGATGAGAGACCACACAA
GTGCCATCTCTGTGGCAGGGCATTCAGAACAGTCACCCTCCTGAGGAATCACCTTAAC
ACACACACAGGTACTCGTCCTCACAAGTGCCCAGACTGCGACATGGCCTTTGTGACCA
GTGGAGAATTGGTTCGGCATCGTCGTTACAAACACACCCACGAGAAGCCATTCAAGTG
TTCCATGTGCGATTACGCCAGTGTAGAAGTCAGCAAATTAAAACGTCACATTCGCTCTC
ATACTGGAGAGCGTCCGTTTCAGTGCAGTTTGTGCAGTTATGCCAGCAGGGACACATA
CAAGCTGAAAAGGCACATGAGAACCCATTCAGGGGAAAAGCCTTATGAATGTTATATT
TGTCATGCTCGGTTTACCCAAAGTGGTACCATGAAGATGCACATTTTACAGAAGCACAC
AGAAAATGTGGCCAAATTTCACTGTCCCCACTGTGACACAGTCATAGCCCGAAAAAGT
GATTTGGGTGTCCACTTGCGAAAGCAGCATTCCTATATTGAGCAAGGCAAGAAATGCC
GTTACTGTGATGCTGTGTTTCATGAGCGCTATGCCCTCATCCAGCATCAGAAGTCACAC
AAGAATGAGAAGCGCTTTAAGTGTGACCAGTGTGATTACGCTTGTAGACAGGAGAGGC
ACATGATCATGCACAAGCGCACCCACACCGGGGAGAAGCCTTACGCCTGCAGCCACTG
CGATAAGACCTTCCGCCAGAAGCAGCTTCTCGACATGCACTTCAAGCGCTATCACGAC
CCCAACTTCGTCCCTGCGGCTTTTGTCTGTTCTAAGTGTGGGAAAACATTTACACGTCG
GAATACCATGGCAAGACATGCTGATAATTGTGCTGGCCCAGATGGCGTAGAGGGGGAA
AATGGAGGAGAAACGAAGAAGAGTAAACGTGGAAGAAAAAGAAAGATGCGCTCGAA
GAAAGAAGATTCCTCTGACAGTGAAAATGCTGAACCAGATCTGGACGACAATGAGGA
TGAGGAGGAGCCTGCCGTAGAAATTGAACCTGAGCCAGAGCCTCAGCCTGTGACCCC
AGCCCCACCACCCGCCAAGAAGCGGAGAGGACGACCCCCTGGCAGAACCAACCAGC
CCAAACAGAACCAGCCAACAGCTATCATTCAGGTTGAAGACCAGAATACAGGTGCAAT
TGAGAACATTATAGTTGAAGTAAAAAAAGAGCCAGATGCTGAGCCCGCAGAGGGAGA

GGAAGAGGAGGCCCAGCCAGCTGCCACAGATGCCCCCAACGGAGACCTCACGCCCGAGATGATCCTCAGCATGATGGACCGGTGAGCTAGCCTGTGGAATGTGTGTCAGTTAGGG

Table S10

Gene symbol	GenBank_ID	species
ctcf	NC_000074.7	mouse

ctcf Lentiviral Vector (Mouse) (CMV) (pLenti-GIII-CMV) (Neo)

Insertion sequence :

ATGGAAGGTGAGGCGGTTGAAGCCATTGTGGAGGAGTCTGAAACTTTCATTAAAGGAAAAGAAAGAAAGACTTACCAGAGACGCCGGGAAGGGGGCCAGGAAGAAGATGCTTGCCACCTGCCCCAGAACCAGACAGATGGGGGTGAGGTGGTCCAGGATGTCAACAGCAGTGTACAGATGGTAATGATGGAACAGCTGGATCCTACCCTTCTCCAGATGAAGACTGAAGTCATGGAGGGTACAGTGGCTCCGGAAGCAGAGGCTGCAGTGGACGATACCCAGATCATAACCTTGCAGGTTGTAAATATGGAGGAACAGCCCATTAACATAGGAGAGCTTCAGCTTGTCCAAGTACCTGTTCCTGTGACGGTACCTGTTGCTACTACTTCAGTAGAAGAACTTCAGGGGGCTTATGAGAATGAAGTGTCTAAAGAGGGCCTTGCAGAAAGTGAACCGATGATATGTCACACCTTACCTTTGCCTGAAGGATTTCAGGTGGTGAAAGTGGGGGCCAATGGAGAAGTGGAGACACTAGAGCAGGGCGAGCTTCCTCCTCAGGAAGACTCTAGCTGGCAAAAAGACCCAGACTATCAGCCACCAGCCAAAAAAACAAAGAAAACCAAAAAGAGCAAACTTCGTTACACAGAAGAGGGCAAAGACGTGGATGTGTCTGTGTATGATTTTGAGGAAGAACAGCAGGAAGGACTGCTGTCTGAGGTTAATGCAGAGAAAGTAGTTGGTAATATGAAGCCTCCGAAGCCAACAAAAATTAAAAAAAAAGGTGTAAAGAAAACATTCCAGTGTGAGCTTTGCAGTTACACATGTCCCCGGCGTTCAAATTTGG

Table S11

Gene symbol	GenBank_ID	species
mien1	NC_000077.7	mouse

mien1-set shRNA in pLenti-U6-shRNA-CMV-Puro Vector (Mouse)

Target a-146:

AGTATCCGGGCATTGAGATTGTTCAAGAGACAATCTCAATGCCCGGATACT

Target b-195:

CGAGATTGAGATCAATGGACATTCAAGAGATGTCCATTGATCTCAATCTCG

Target c-290:

GCAATGGAGAACCTGTAGAAATTCAAGAGATTTCTACAGGTTCTCCATTGC

Table S12

Gene signature table for Neu1, Neu2, and Neu3 subpopulations

cluster	gene	cluster	gene	cluster	gene
Neu1	*HLA-B*	Neu2	*RPS18*	Neu3	*S100A12*
Neu1	*FTH1*	Neu2	*RPL13*	Neu3	*S100A8*
Neu1	*G0S2*	Neu2	*RPL10*	Neu3	*S100A9*
Neu1	*CXCR4*	Neu2	***IL1B***	Neu3	*S100A6*
Neu1	*NEAT1*	Neu2	***MIEN1***	Neu3	*HMGB2*
Neu1	*PHACTR1*	Neu2	*RPS23*	Neu3	*S100A4*
Neu1	*ITM2B*	Neu2	*RPL39*	Neu3	*RGS2*
Neu1	*CCL4L2*	Neu2	*RPS12*	Neu3	*VNN2*
Neu1	*LINC01272*	Neu2	*RPL10A*	Neu3	*CDA*
Neu1	*IGSF6*	Neu2	*RPL18A*	Neu3	*IFRD1*
Neu1	*AIF1*	Neu2	*RPS8*	Neu3	*FABP1*
Neu1	*NFKBIA*	Neu2	*RPS4X*	Neu3	*MME*
Neu1	*PSMB9*	Neu2	*RPL35*	Neu3	*ACSL1*
Neu1	*SEC14L1*	Neu2	*RPS13*	Neu3	*GCA*
Neu1	*CCL3L3*	Neu2	*HSPA1B*	Neu3	*LRRK2*
Neu1	*FCER1G*	Neu2	*RPS6*	Neu3	*PHGR1*
Neu1	*HLA-E*	Neu2	*RPS19*	Neu3	*CD55*
Neu1	*EFHD2*	Neu2	*RPL36*	Neu3	*TSPO*
Neu1	*ISG20*	Neu2	*RPL23A*	Neu3	*MEGF9*
Neu1	*TNFAIP3*	Neu2	*RPL22*	Neu3	*FAM65B*
Neu1	*C15orf48*	Neu2	*IGHG4*	Neu3	*NCF1*
Neu1	*FYB*	Neu2	*RPL11*	Neu3	*CYP4F3*
Neu1	*BRI3*	Neu2	*RPL8*	Neu3	*VIM*
Neu1	*LAPTM5*	Neu2	*RPL29*	Neu3	*FKBP5*
Neu1	*TNFAIP2*	Neu2	*RPL5*	Neu3	*ABTB1*
Neu1	*IGHA1*	Neu2	*RPS3A*	Neu3	*BASP1*
Neu1	*NOP10*	Neu2	*RPLP2*	Neu3	*CORO1A*
Neu1	*FLOT1*	Neu2	*RPL3*	Neu3	*SELL*
Neu1	*RAC1*	Neu2	*HSPA1A*	Neu3	*CTB-61M7.2*
Neu1	*CCL4*	Neu2	*RPL14*	Neu3	*LYZ*

Neu1	*NCF2*	Neu2	*RPSA*	Neu3	*STK17B*
Neu1	*IFITM3*	Neu2	*GSTP1*	Neu3	*LAMTOR4*
Neu1	*IFIT2*	Neu2	*EEF1A1*	Neu3	*GMFG*
Neu1	*SNX10*	Neu2	*NPM1*	Neu3	*SLC11A1*
Neu1	*SERPINA1*	Neu2	*RPL12*	Neu3	*ITGB2*
Neu1	*SLA*	Neu2	*RPS5*	Neu3	*S100P*
Neu1	*MARCKS*	Neu2	*CD24*	Neu3	*NFIL3*
Neu1	*TMEM154*	Neu2	*RPL26*	Neu3	*USP10*
Neu1	*TNFAIP6*	Neu2	*IGHG1*	Neu3	*TOB1*
Neu1	*CD53*	Neu2	*RPL36A*	Neu3	*SAMSN1*
Neu1	*IRF1*	Neu2	*RPLP0*	Neu3	*C10orf54*
Neu1	*YPEL3*	Neu2	*HCAR3*	Neu3	*RTN3*
Neu1	*ATP6V1G1*	Neu2	*PPIA*	Neu3	*TFF3*
Neu1	*LYN*	Neu2	*SLC25A5*	Neu3	*LBR*
Neu1	*TNFRSF1B*	Neu2	*HSPD1*	Neu3	*XPO6*
Neu1	*LYST*	Neu2	*RPL31*	Neu3	*HCLS1*
Neu1	*PAK2*	Neu2	*KRT18*	Neu3	*GPSM3*
Neu1	*RILPL2*	Neu2	*GADD45B*	Neu3	*RP6-159A1.4*
Neu1	*LRRFIP1*	Neu2	*SOCS3*	Neu3	*TKT*
Neu1	*C4orf3*	Neu2	*HCAR2*	Neu3	*TUBA4A*

This table highlights the unique gene expression characteristics of each neutrophil subset identified in the study. *MIEN1* and *IL1B* are highlighted in bold black.

Supplementary Methods

Cell Culture and Treatment Conditions

The study utilized multiple cell lines cultured under specific conditions. THP-1 cells (human monocytic leukemia cells, Cat: TCHu 57) were obtained from the China Academy of Sciences (Shanghai, China) and cultured in RPMI-1640 medium supplemented with 10% fetal bovine serum (FBS) and 1% penicillin/streptomycin (P/S). Differentiation into macrophages was induced using 100 nM phorbol 12-myristate 13-acetate (PMA) for 24 hours, followed by PBS washing and a 24-hour resting period in fresh RPMI-1640 medium. HL-60 cells (CL-0110) from Wuhan Pu-nuo-sai Life

Technology Co., Ltd. were cultured in Iscove's Modified Dulbecco's Medium (IMDM) with 20% FBS and 1% P/S, and granulocyte differentiation was achieved using 1.25% DMSO for 5 days [1]. We induced tumor-associated neutrophils by treating HL-60 cells with conditioned media derived from CRC cell lines. The conditioned media was typically mixed with the base medium at a 1:1 ratio for this purpose. Human colorectal tumor cancer-associated fibroblasts (CAFs, Cat: HUM-iCell-d044) from iCell Bioscience Co., Ltd., as well as SW480 (CL-0223), SW620 (CL-0225), and MC38-luc cells (TCM-C790L), were cultured in Dulbecco's Modified Eagle Medium (DMEM) supplemented with 10% FBS and 1% P/S. CAFs were limited to within 10 generations to minimize passage-related effects. ER-Hoxb8-derived neutrophils (ER-Hoxb8-DNs, Cat: T0202) from abm Inc. were maintained in PriGrow II base medium containing 10% FBS, 100 ng/mL stem cell factor (SCF), and 0.5 μM ß-estradiol. Neutrophil differentiation was induced by completely removing ß-estradiol from the medium[2]. Hypoxic treatment was applied to simulate a low-oxygen environment, wherein cells were incubated at 37°C in a triaxial incubator under a hypoxic atmosphere (1% O_2, 5% CO_2, and 94% N_2) for 24 hours. All cell lines were maintained in a humidified incubator at 37°C with 5% CO_2 to ensure optimal growth conditions.

For IL-1β neutralization experiments, cells were treated with 10 μg/ml Raleukin for 12 hours and then washed with PBS to eliminate residual reagent. For NAMPT neutralization, cells were exposed to 5 μM FK866 under the same conditions, followed by thorough PBS washing. Co-culture experiments were conducted in the absence of inhibitors to ensure accurate and unbiased results.

Cell counting kit 8 (CCK8) Assay

The Cell Counting Kit-8 (CCK-8) assay was performed to assess cell viability and proliferation of differentiated HL-60 cells under different treatment conditions. Differentiated HL-60 cells were seeded into 96-well plates at a density of 1×10^4 cells/well and treated with various experimental interventions. At specific time points (24, 48, and 72 hours), CCK-8 reagent was added to each well according to the manufacturer's protocol. Plates were incubated for 2 hours at 37°C, and absorbance was measured at 450 nm using a Bio-Tek microplate reader (EL800, Bio-Tek, USA). Each condition was tested with a sample size of n = 6, and experiments were conducted in triplicate to ensure statistical robustness and reproducibility. Results were expressed as mean ± SD.

Neutrophil chemotaxis Assay

Neutrophil chemotaxis was assessed using a fluorescent chemotaxis assay. Calcein-AM-labeled differentiated HL-60 cells (neutrophils) were seeded into the upper chamber of 8-μm transwell filters (Corning, USA), while the lower chamber contained tumor cells cultured in appropriate medium as a chemoattractant source. The assay was conducted under different treatment conditions. After a 60-minute incubation at 37°C in a 5% CO_2 incubator, the transwell membranes were carefully removed. Migrated neutrophils adhering to the lower surface of the membrane were imaged using a fluorescence microscope (BX-63, Olympus, Japan). The number of migrated cells was quantified using ImageJ software (NIH, USA). Each experimental condition was

performed in triplicate, and results were expressed as mean ± SD to ensure reproducibility and statistical robustness.

Luciferase reporter Assay

HL60 cells were co-transfected with a luciferase reporter plasmid containing the target promoter region (e.g., MIEN1 promoter) and a Renilla luciferase plasmid as an internal control using Lipofectamine 3000. After 48 hours, luciferase activity was measured using the Dual-Luciferase Reporter Assay System (Promega) and normalized to Renilla activity. Experiments were performed in triplicate.

ChIP-qPCR Assay

A total of 2×10^7 HL60 cells were washed with phosphate-buffered saline (PBS) and cross-linked with 1% formaldehyde for 10 minutes at room temperature. Cross-linking was quenched with 0.125 M glycine for 5 minutes. Cells were pelleted and washed twice with PBS, then lysed on ice for 5 minutes in lysis buffer (10 mM HEPES, pH 7.5; 0.1 mM EDTA; 0.5% NP-40; protease inhibitors). Nuclei were collected by centrifugation (2000 × g, 10 minutes, 4 °C), and chromatin was sheared by sonication to an average size of 100–500 bp. Ten percent of the sonicated chromatin was reserved as input, while 80% was used for immunoprecipitation with an anti-CTCF antibody and 10% with rabbit IgG as a negative control. Immunocomplexes were washed, eluted, and subjected to reverse cross-linking. DNA from input and IP samples was purified using the phenol-chloroform method and quantified with a Qubit 3 fluorometer.

Chromatin fragmentation was verified by agarose gel electrophoresis of input DNA. Quantitative PCR was performed using SYBR Green Master Mix on a real-time PCR system to assess enrichment of target regions. Experiments were performed in triplicate.

Enzyme-linked immunosorbent assay (ELISA)

The levels of IL-1β and NAMPT in cell culture supernatants were quantified using ELISA kits according to the manufacturer's instructions. Briefly, culture supernatants were collected without replenishing the medium to avoid dilution. Samples were transferred to fresh tubes and centrifuged at 3500 rpm for 10 minutes at room temperature to remove debris. The optical density (OD) at 450 nm was measured using a microplate reader (ELX800, Bio-Tek, USA). All assays were performed in triplicate.

Transmission Electron Microscopy (TEM)

HL60 cells and CAFs were fixed with 2.5% glutaraldehyde in 0.1 M phosphate buffer (pH 7.4) at 4°C, followed by post-fixation with 1% osmium tetroxide. The cells were dehydrated in a graded ethanol series, embedded in epoxy resin, and sectioned into ultrathin slices (70–90 nm). Sections were stained with 0.3% lead citrate and uranyl acetate. Images were captured using a transmission electron microscope (HT7800, Hitachi, Japan).

Phalloidin Staining

F-actin, a major component of microfilaments, was stained using rhodamine-labeled phalloidin. Briefly, cells were fixed with 4% paraformaldehyde for 15 minutes at room

temperature, permeabilized with 0.1% Triton X-100 for 5 minutes, and incubated with rhodamine-phalloidin for 30 minutes in the dark. After washing with PBS, cellular microfilaments were visualized under a fluorescence microscope as previously described.

Tumor cell invasion Assay

CRC cell invasion was assessed using Matrigel-coated Transwell chambers (24-well, 8 μm pore size; Corning). CRC cells (2×10^4/well) were resuspended in 200 μL serum-free DMEM and added to the upper chamber. Differentiated HL60 cells (2×10^4/well), treated under different conditions, were seeded into the bottom chamber containing 500 μL DMEM supplemented with 10% FBS as a chemoattractant.

After 24 hours of incubation at 37°C, non-invaded cells on the upper membrane surface were removed by gentle washing and swabbing. Cells that traversed the membrane and adhered to the lower surface were fixed with 4% formaldehyde and stained with 0.1% crystal violet. Migrated cells were quantified under a phase-contrast microscope (CKX 41, Olympus, Japan) by counting at 6 random fields per membrane.

Wound Healing Assay in Non-Contact Co-Culture System

The wound healing assay was performed in a non-contact co-culture system to assess CRC cell migration influenced by HL60-derived neutrophils. CRC cells (4×10^5/well) were seeded into the lower chamber of a Transwell system (0.4 μm pore size, Corning) and cultured in serum-free medium for 24 hours. A linear wound was introduced in the

CRC cell monolayer using a 200-μL pipette tip. Differentiated HL60 cells treated under different conditions, were seeded into the upper chamber at appropriate densities. The system was incubated at 37°C in a humidified environment. Wound closure was observed and photographed at 0, 24, and 48 hours under a phase-contrast microscope (CKX 41, Olympus, Japan). The wound healing rate was calculated by measuring the reduction in wound area using ImageJ software.

Tumor Sphere Formation Assay in Non-Contact Co-Culture System

To investigate the effect of HL60-derived neutrophils on tumor sphere formation and cancer stemness, a non-contact co-culture system was employed. SW480 and SW620 were seeded at 5×10^3/well in ultra-low attachment 6-well plates (Corning) in serum-free DMEM medium supplemented with 20 ng/mL EGF, 10 ng/mL bFGF, 1× B27 supplement, and 1% penicillin/streptomycin. Differentiated HL60-derived neutrophils (2×10^4/well) were added to the upper chamber of a 0.4 μm Transwell insert (Corning) to allow paracrine signaling without direct cell contact. The co-culture system was maintained at 37°C with 5% CO_2 for 7 days, with medium replaced every 3 days. Tumor sphere formation was monitored under a phase-contrast microscope (Olympus CKX 41, Olympus, Hachioji, Japan), and spheres >50 μm in diameter were counted using ImageJ software. Sphere number were quantified to evaluate the influence of HL60-derived neutrophils on CRC cell stemness.

Chromatin Immunoprecipitation (ChIP) and Quantitative PCR (qPCR)

Cells were crosslinked with 1% formaldehyde, lysed, and sonicated to shear chromatin. Chromatin was immunoprecipitated with anti-CTCF or control IgG antibodies using Protein A/G magnetic beads. After washing and reverse crosslinking, DNA was purified and analyzed by qPCR using primers targeting the MIEN1 promoter. Results were normalized to input DNA and expressed as % input.

Western blotting (WB)

Proteins were extracted using Radioimmunoprecipitation Assay (RIPA) buffer, and concentrations were determined using the Bradford assay. Equal amounts of protein (20 μg per sample) were separated on SurePAGE Bis–Tris gradient gels (10% or 12%) and transferred onto polyvinylidene fluoride (PVDF) membranes. The membranes were blocked with blocking solution at room temperature for 1 hour, followed by overnight incubation at 4°C with primary antibodies. After three washes with Tris-buffered saline containing 0.05% Tween-20 (TBST), membranes were incubated with fluorescently labeled secondary antibodies (Licor Odyssey) for 1 hour at room temperature (22-25°C). Protein bands were visualized using the Licor Odyssey fluorescence imaging system (Licor biotechnology, US), with β-tubulin, β-actin, or GAPDH as loading controls for normalization.

Hematoxylin/eosin (HE) staining

We followed standard procedures for HE staining[3]. After deparaffinization and

rehydration, tissue sections were stained with a hematoxylin solution for 5 minutes, followed by 5 dips in 1% acid ethanol (1% HCl in 75% ethanol), and then rinsed in distilled water. The sections were stained with eosin solution for 3 minutes, then dehydrated with graded alcohol and cleared in xylene. We examined and photographed the mounted slides with a microscope (Nikon Eclipse NI-E, Nikon, Japan).

Immunofluorescence

Cells were seeded onto glass slides in 24-well culture plates. After indicated treatment, cells were fixed with formaldehyde (4%) and permeabilized with 0.3% Triton X-100. The slides were then washed by PBS and incubated with primary antibodies overnight. Next, the slides were stained with appropriate secondary antibodies and 4, 6-diamidino-2-phenylindole (DAPI).

The multicolor immunofluorescence assessment for tumor tissue was based on the tyramide signal amplification (TSA) system. In brief, the sliced tissue specimens were dewaxed, rehydrated, treated for Heating-induced epitope retrieval (HIER) with H2O2, blocked using 3% Bovine serum albumin (BSA) to inhibit nonspecific interaction, labeled with primary and then with horseradish peroxidase (HRP)-conjugated anti-rabbit secondary antibodies and fluorescent tyramide successively. Then the sections were treated for HIER, BSA blocking, and antibody staining again; lastly, the nuclei were dyed with DAPI, and imaged under fluorescence microscope (DS-QilMC, Nikon, Japan). The mean fluorescence intensity (MFI) of each cell was calculated from the total fluorescence intensity of a whole cell divided by the cell area. Statistics were based

on measurements for at least 30 cells[4].

Mitochondrial Morphology Analysis Using MitoTracker Red

To evaluate mitochondrial structure, CAFs were cultured in 24-well glass-bottom confocal plates in a non-contact co-culture system with HL60-derived neutrophils (upper chamber), which were subjected to different treatments. After 24 hours of co-culture, CAFs were stained with MitoTracker Red (100 nM) for 30 minutes at 37°C in the dark and washed with PBS. Nuclei were counterstained with DAPI for 10 minutes. Images were acquired using a confocal microscope (FV4000, Olympus, Japan), and mitochondrial morphology was assessed by quantifying the average area (μm^2) and average perimeter (μm) of mitochondria. Fragmented mitochondria were characterized by smaller areas and shorter perimeters, whereas filamentous mitochondria displayed larger areas and longer perimeters. ImageJ software was used for morphological measurements across six random fields of view per group.

Mitochondrial Membrane Potential Assay (JC-1 Staining)

CAFs were seeded in 24-well glass-bottom confocal plates and cultured in the lower chambers of a non-contact co-culture system, while HL60-derived neutrophils, subjected to different treatments were placed in the upper Transwell inserts. After 24 hours of co-culture, CAFs were incubated with JC-1 dye in serum-free medium for 20 minutes at 37°C in the dark, washed twice with PBS, and fixed with 4% paraformaldehyde for 10 minutes. Fluorescent images of JC-1 aggregates (red,

polarized mitochondria) and monomers (green, depolarized mitochondria) were captured using a confocal microscope (FV4000, Olympus, Japan). The red-to-green fluorescence ratio was quantified using ImageJ software to assess mitochondrial membrane potential.

Mitochondrial respiration and glycolysis Analysis

Live-cell respiration and glycolysis were assessed in CAFs using the XFe24 Extracellular Flux Analyzer (Seahorse Bioscience, USA). CAFs (5×10^4 cells/well) were cultured in 24-well plates within a non-contact co-culture system, where HL60-derived neutrophils, treated under different conditions, were seeded into the upper Transwell inserts (0.4 μm pore size). After 24 hours, the culture medium was replaced with 500 μL of XF assay medium (supplemented with 1 mM pyruvate, 2 mM glutamine, and 10 mM glucose), centrifuged at 200×g for 3 minutes, and incubated at 37°C without CO_2 for 1 hour. Mitochondrial function was measured using the Mito Stress Test with sequential injections of oligomycin (1.3 μM), FCCP (2 μM followed by 3 μM), and antimycin A (2.5 μM) to determine basal respiration (BR), maximal respiration (MR), spare respiratory capacity (SRC), and mitochondrial ATP production. Glycolysis was assessed using the Glycolysis Stress Test with sequential injections of glucose (10 mM), oligomycin (4 μM), and 2-deoxy-D-glucose (2-DG, 100 mM), from which basal glycolysis, glycolytic capacity, and non-glycolytic acidification were calculated. Following measurements, cells were lysed with M-PER reagent, and total protein content was quantified using the bicinchoninic acid (BCA) assay (Pierce). OCR and

ECAR data were normalized to protein content.

3D Multicellular Spheroid Formation Assay

The 3D co-culture spheroid experiment was modified from Dolznig et al[5]. To assess the effects of neutrophil-conditioned medium on 3D multicellular spheroid formation, experiments were performed in a 96-well ultra-low attachment, glass-bottom confocal plate. On Day 1, mCherry-labeled CRC cells (1,000 cells/well) were seeded into each well and allowed to aggregate into spheroids by incubating the plate undisturbed for 24 hours. On Day 3, GFP-labeled CAFs (1,000 cells/well) and CellTracker Blue CMAC-labeled macrophages (1,000 cells/well) were added to each well. The plate was centrifuged at 200 × g for 4 minutes to promote cell sedimentation, and the supernatant was carefully removed. Neutrophil-conditioned medium (50 μL), collected from HL60-derived neutrophils treated under different conditions, was mixed 1:1 with 50 μL of Matrigel and added to each well to promote spheroid formation. The plate was incubated at 37°C with 5% CO_2 to allow spheroid maturation. On Day 7, spheroid structure and cellular distribution were visualized using a confocal microscope (FV4000, Olympus, Japan). mCherry (red, tumor cells), GFP (green, CAFs), and CellTracker™ Blue CMAC (blue, macrophages) fluorescence signals were captured, and merged images were analyzed to assess spheroid formation and cellular interactions.

Flow cytometry

T cells/neutrophils were resuspended in 50 μL of PBS and stained with specific

antibody panels in the dark at 4°C for 30 minutes. After staining, cells were washed with PBS containing 5% FBS to remove unbound antibodies. Flow cytometry was performed using a multiple parameter cytometer (Guilin Ulead Medical Electronics, URIT bf-730, China), and the acquired data were analyzed using FlowJo v10 software.

Orthotopic mouse tumor model

Orthotopic CRC mouse model was established using MC38-luc cells. Five-week-old male C57BL/6J mice (20g-22g), purchased from the Beijing Weitong Lihua Experimental Animal Technology Co., Ltd. (Certificate No. SYXK2019-0010), were housed under standard conditions and cared for according to institutional guidelines for animal care. Mice were anesthetized with 2% isoflurane, and a small right-sided abdominal incision was made to expose the cecum. The cecum was gently placed on a scalpel holder, flattened, and stabilized with forceps to prevent tumor cell leakage into the cecal lumen or abdominal cavity. MC38-luc cells (1×10^6 cells in 50 μL PBS) were injected into the cecal wall using a sterile insulin syringe. Light pressure was applied to the injection site to prevent leakage, and the cecum was carefully returned to the abdominal cavity. The peritoneum and skin were closed using sutures and wound clips.

Experiment 1: In Vivo Bioluminescence Imaging of Tumor Progression

In the first experiment, one-week post-surgery, mice were randomized into four groups based on different genetic manipulations of ER-Hoxb8-DNs: NC, oe-CTCF, oe-CTCF + sh-MIEN1, oe-CTCF + anti-IL-1β, oe-CTCF with anti-mouse IL-1β treatment. Mice in this group received anti-mouse IL-1β monoclonal antibody (1.0 mg/kg, i.p., twice

weekly). ER-Hoxb8-DNs (1×10^5 cells per injection) were administered intraperitoneally every three days across all groups. Tumor progression and metastasis were monitored in the fourth postoperative week using bioluminescence imaging. Mice were intraperitoneally injected with 100 mg/kg d-luciferin and anesthetized by inhaling 2.5% isoflurane for 5–6 minutes in a volatilization chamber. Bioluminescence imaging was performed using the BLT multimodal animal imaging system (BLT Photon Tech, Aniview SE, China). The software automatically superimposed grayscale photographic images and pseudocolored bioluminescent images to match the luciferase signals to their respective anatomical locations, facilitating tumor burden and metastasis assessment. After imaging, tumor-bearing mice were euthanized using CO_2, and primary tumors and livers were excised for further examination. The euthanasia was performed in accordance with the American Veterinary Medical Association (AVMA's) Guidelines for Humane Animal Euthanasia.

Experiment 2: In the second experiment, one-week post-surgery, mice were randomized into five groups: NC, oe-CTCF, oe-CTCF + sh-MIEN1, oe-CTCF + anti-IL-1β, and oe-CTCF + anti-NAMPT. The anti-NAMPT group received FK866 (20mg/kg, i.p., once daily)[6], while other treatment regimens were consistent with Experiment 1. ER-Hoxb8-DNs (1×10^5 cells per injection) were administered intraperitoneally every three days. Four weeks post-surgery, primary tumors were excised following CO_2 euthanasia for immunofluorescence analysis.

All in vivo investigations were authorized by the ethical board of the Animal Ethics Committee of Jiangsu Province Hospital of Chinese Medicine (2024DW-033-01).

liver metastatic model

The intrasplenic injection model was established with reference to previously published studies [7]. The liver metastasis model was established using MC38-luc cells with procedures consistent with the orthotopic colorectal cancer model, including mouse care, anesthesia, imaging, and ethical considerations. Mice were anesthetized with 2% isoflurane, and a small abdominal incision was made to expose the spleen. The spleen was carefully divided into two independent sections. MC38-luc cells (5×10^5 cells in 50 μL PBS) were injected into one half of the spleen using a sterile insulin syringe. After 90 seconds to allow tumor cells to seed the liver, the injected half-spleen was surgically removed to prevent ectopic tumor growth while preserving the tumor-free half of the spleen to maintain normal immune function. The peritoneum and skin were closed using sutures. Post-surgery, mice were randomized into four groups consistent with the orthotopic tumor model. Four weeks post-surgery, bioluminescence imaging was performed as described in the orthotopic model. After imaging, mice were euthanized using CO_2, and the livers were excised for examination and quantification of metastatic lesions.

Orthotopic and Liver Metastasis Tumor Models with Immunotherapy

We modified the immunotherapy model from a previously described protocol[8]. Tumor models were established as described above. Briefly, orthotopic and liver metastasis models were created using MC38-luc cells in C57BL/6J mice. ER-Hoxb8-DNs with different genetic manipulations (NC, oe-CTCF, oe-CTCF + sh-MIEN1) were

administered intraperitoneally every three days starting one-week post-surgery.

The treatment groups included:

NC (negative control); NC + anti-PD-L1: Anti-PD-L1 monoclonal antibody (200 μg, i.p., twice weekly for 3 weeks); oe-CTCF + anti-PD-L1: Over-expressing CTCF with anti-PD-L1 treatment; oe-CTCF + sh-MIEN1 + anti-PD-L1: Over-expressing CTCF with *MIEN1* knockdown and anti-PD-L1 treatment; oe-CTCF + anti-IL-1β + anti-PD-L1: Over-expressing CTCF with anti-IL-1β (1.0 mg/kg, i.p., twice weekly for 3 weeks) and anti-PD-L1. Immunotherapy was administered for 3 weeks. Following the final dose, therapeutic efficacy was evaluated by in vivo bioluminescence imaging. Mice were intraperitoneally injected with 100 mg/kg d-luciferin and anesthetized with 2% isoflurane. Imaging was performed using the BLT multimodal imaging system. Bioluminescence intensity (p/s/cm²/sr) was measured to assess tumor progression and treatment response. For the liver metastasis model, livers were collected after imaging for histological analysis, including HE staining, to quantify metastatic lesions.

Supplementary Figures

Figure S1

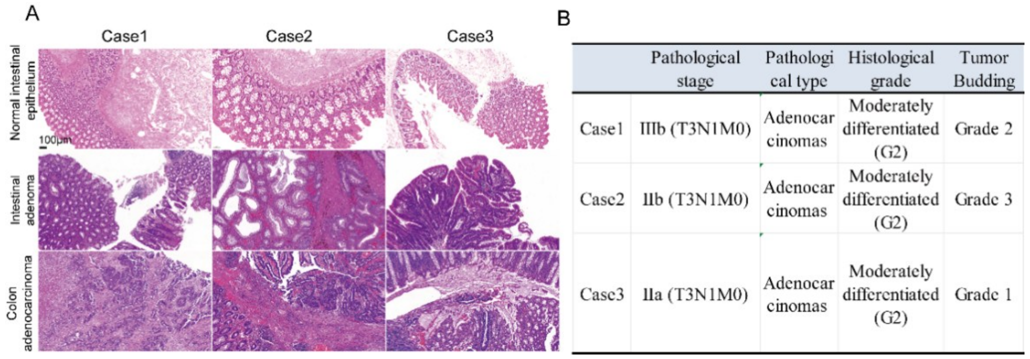

	Pathological stage	Pathological type	Histological grade	Tumor Budding
Case1	IIIb (T3N1M0)	Adenocarcinomas	Moderately differentiated (G2)	Grade 2
Case2	IIb (T3N1M0)	Adenocarcinomas	Moderately differentiated (G2)	Grade 3
Case3	IIa (T3N1M0)	Adenocarcinomas	Moderately differentiated (G2)	Grade 1

Figure S1. (A) H&E-stained sections of matched normal mucosa, adenoma, and

adenocarcinoma from three patients. Scale bar: 100 μm.

(B) Clinicopathological features of the three cases. These matched samples were used for single-cell sequencing.

Figure S2

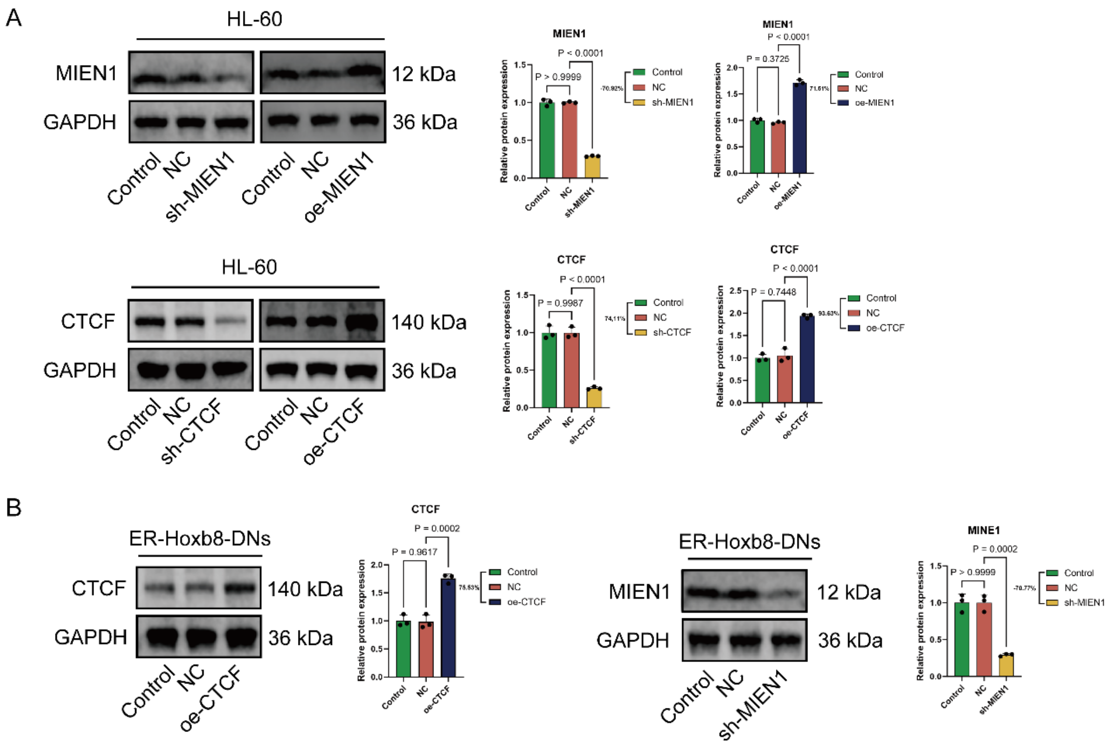

Figure S2. Validation of Transfection Efficiency for CTCF and MIEN1 in HL-60 and ER-Hoxb8-DNs Cells.

(A) Western blot analysis of MIEN1 and CTCF expression in HL-60 cells following shRNA knockdown (sh-MIEN1, sh-CTCF) or over-expression (oe-MIEN1, oe-CTCF). GAPDH serves as a loading control. Quantified protein expression levels are shown on the right.

(B) Western blot analysis of CTCF over-expression (oe-CTCF) and MIEN1 knockdown (sh-MIEN1) in ER-Hoxb8-DNs cells. GAPDH serves as a loading control. Quantifications are presented on the right.

Data are represented as mean ± SD from three independent experiments ($P < 0.0001$, $P = 0.0002$).

Figure S3

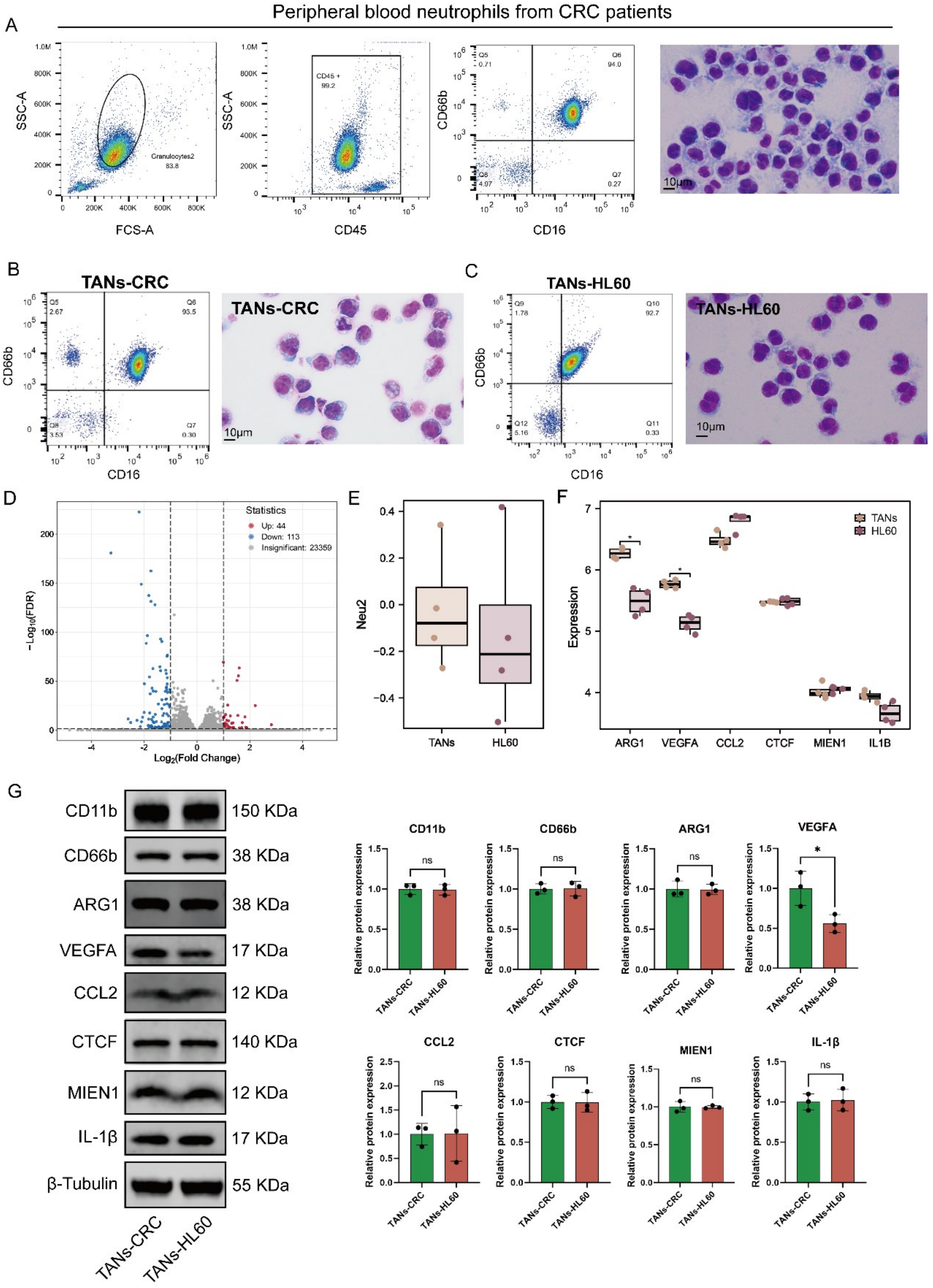

Figure S3. Characterization of Neutrophils from Peripheral Blood, CRC Tumor Tissues, and HL60-Derived Cells.

(A-C) Flow cytometry and Giemsa staining were performed to assess the purity and morphology of neutrophils isolated from peripheral blood of CRC patients (A), CRC tumor tissues (B), and HL60-derived TANs (C). All three sources yielded high-purity $CD45^+CD11b^+CD66b^+$ neutrophils with characteristic segmented nuclear morphology, confirming successful isolation or induction. Scale bar: 10 μm.

(D) Volcano plot showing differential gene expression between primary tumor-associated neutrophils (TANs) and HL60-derived TANs. Genes up-regulated (red) and down-regulated (green) are highlighted, with the number of significant genes indicated.

(E) Box plot showing the expression level of the Neu2-associated gene signature in primary CRC-derived TANs and HL60-induced TANs.

(F-G) Validation of Neu2 marker expression in CRC-derived TANs and HL60-derived TANs at mRNA transcription level (F) and protein level (G).

Data are presented as mean ± SD, statistical significance was assessed using Student's t-test, with *P < 0.05, **P < 0.01, and ***P < 0.001 indicating significant differences.

Figure S4

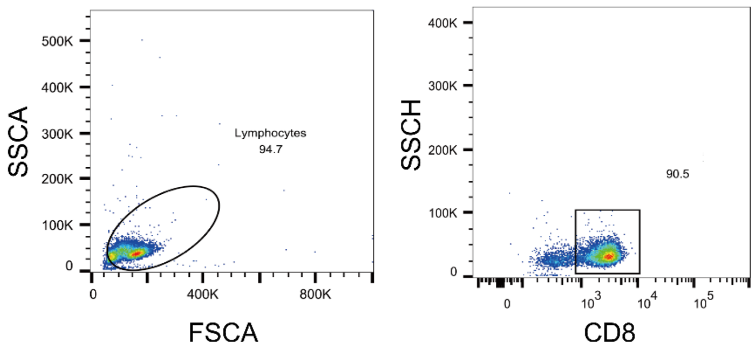

Figure S4. Flow cytometry analysis showed that 90.5% of the magnetic bead-sorted cells expressed CD8, confirming high purity.

Figure S5

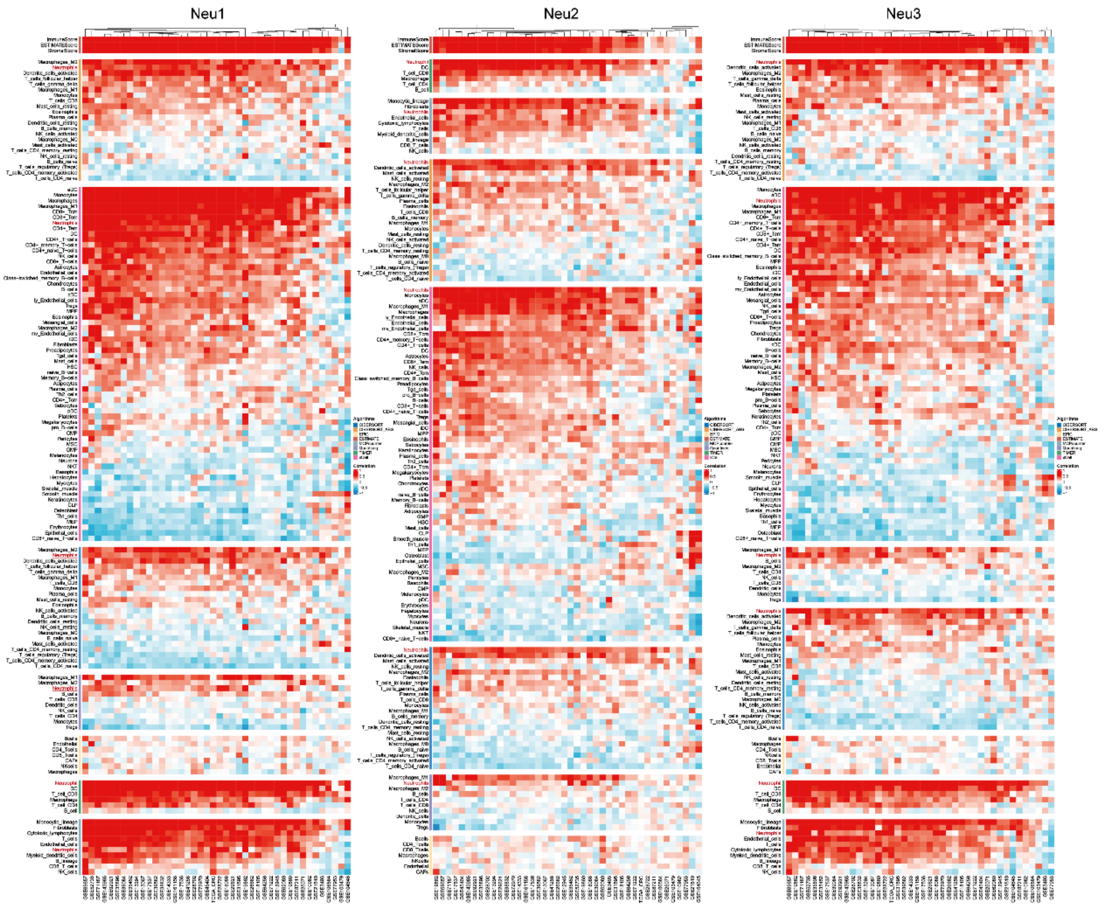

Figure S5. Correlation analysis between cell types and Neu1, Neu2, and Neu3 subpopulations across datasets.

The heatmap shows the correlation between cell types (rows, defined by different algorithms) and Neu1-3 (columns) across different datasets. Red represents positive correlation, while blue represents negative correlation. Neu1, Neu2, and Neu3 exhibit strong positive correlations with neutrophil-specific cell types across datasets, validating their classification as distinct neutrophil subpopulation.

Figure S6

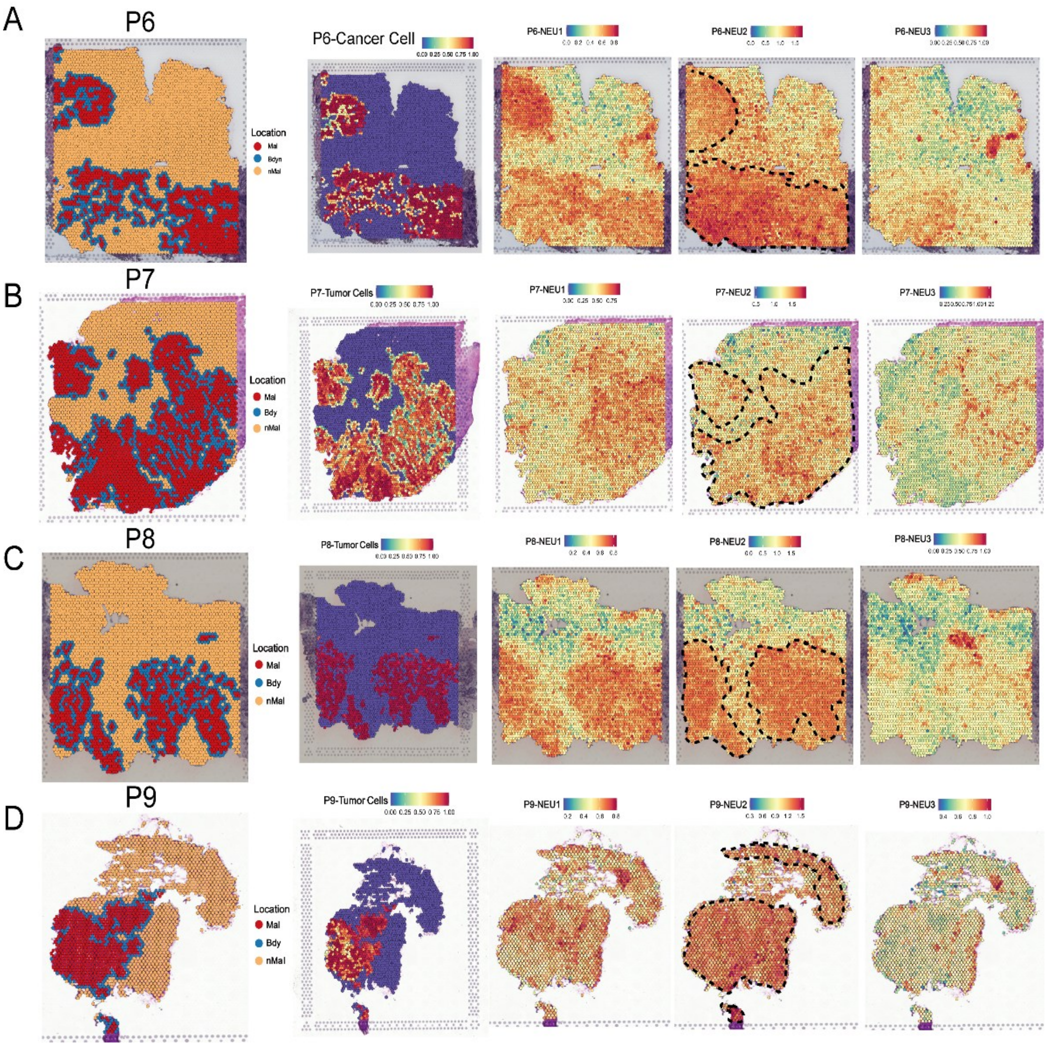

Figure S6. Spatial distribution and subtype analysis of Neu1, Neu2, and Neu3 across tumor samples (P6–P9).

(A-D) Spatial transcriptomics maps of four patient samples (P6–P9) showing defined regions: Malignant Area (Ma), Boundary Area (Bdy), and Non-malignant Area (nMal). Left panel: Classification of tissue areas into Ma, Bdy, and nMal regions based on transcriptomic profiles. Second panel: Spatial distribution of tumor cells, highlighting regions enriched in malignant cells. Right three panels: Expression scores of Neu1, Neu2, and Neu3 subpopulations, with contour lines outlining regions of higher

expression.

Figure S7

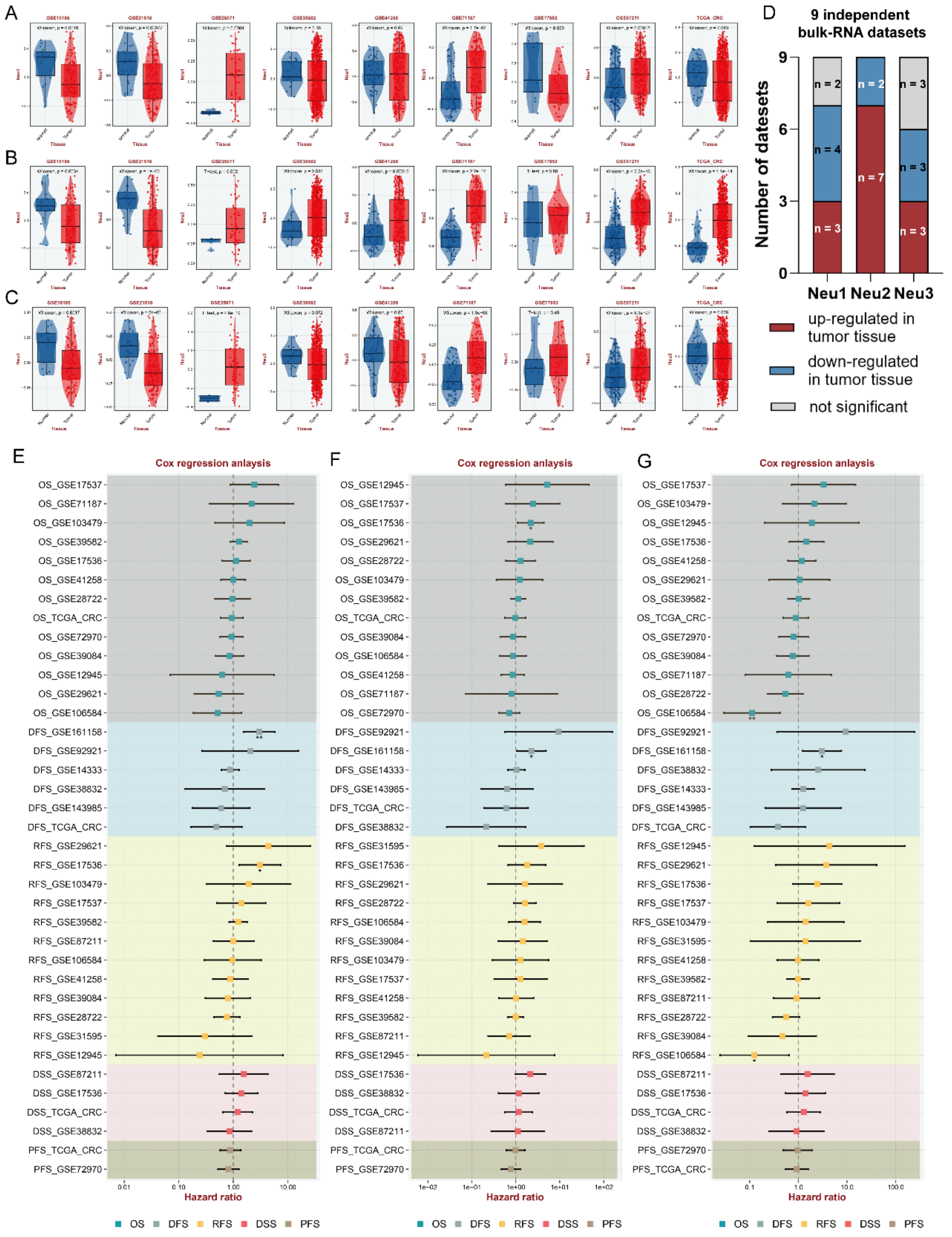

Figure S7. Expression levels of Neu1, Neu2, and Neu3 subpopulations in normal

and tumor tissues across multiple datasets.

(A-C) Violin plots showing the expression of Neu1 (A), Neu2 (B), and Neu3 (C) in normal (blue) and tumor (red) tissues across nine datasets (GSE18105, GSE21510, GSE25071, GSE39582, GSE41258, GSE71187, GSE77953, GSE87211, and TCGA_CRC).

(D) Summary of Neu subpopulation expression changes across 9 transcriptomic datasets. Neu2 is consistently up-regulated in tumor tissues (red), Neu3 is predominantly down-regulated (blue), and Neu1 shows variable expression patterns.

(E-G) Forest plots for overall survival of Neu1 (D), Neu2 (E), and Neu3 (F) in subgroups.

Figure S8

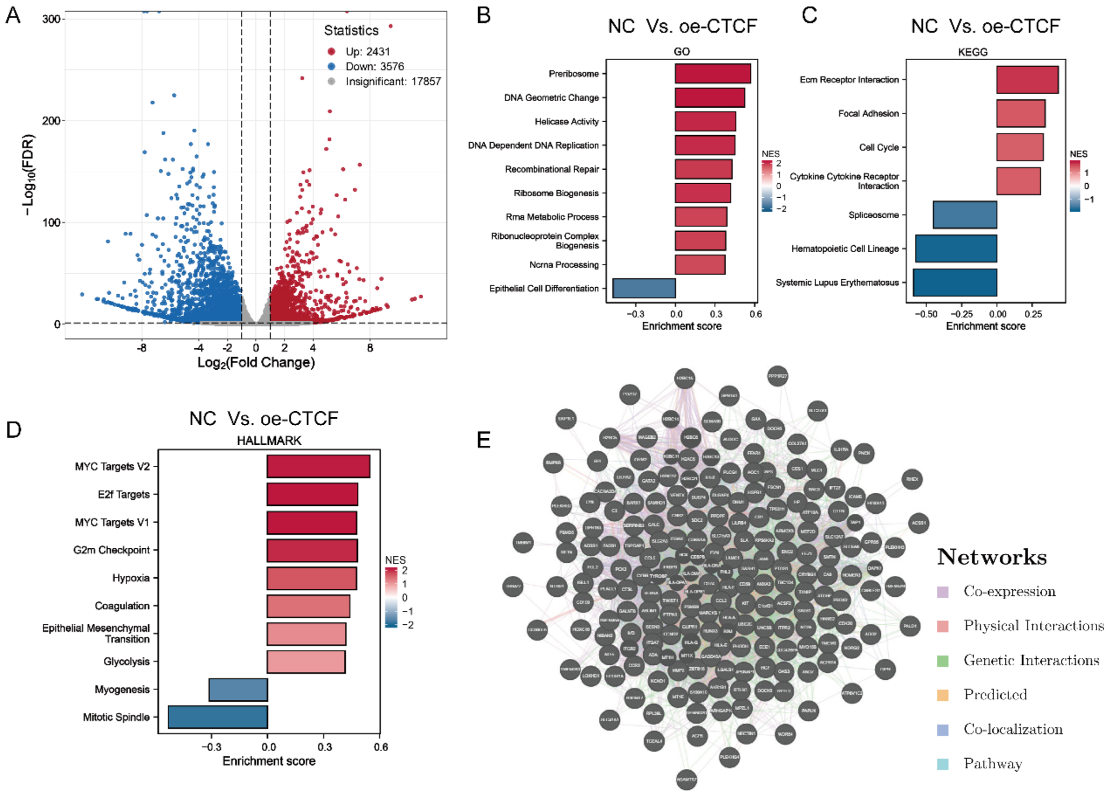

Figure S8. CTCF-regulated pathways and networks in HL60-derived tumor-

associated neutrophils (TANs).

(A) Volcano plot showing the differential expression of genes between NC and oe-CTCF groups. Genes up-regulated (red) and down-regulated (blue) are highlighted, with the number of significant genes indicated.

(B-D) Functional enrichment analyses, including Gene Ontology (GO) (B), Kyoto Encyclopedia of Genes and Genomes (KEGG) pathways (C), and Hallmark gene sets (D), demonstrate the biological processes and pathways affected by CTCF over-expression in TANs.

(E) Protein-protein interaction (PPI) network depicting the complex molecular interactions regulated by CTCF in TANs. Network edges represent various types of interactions.

Figure S9

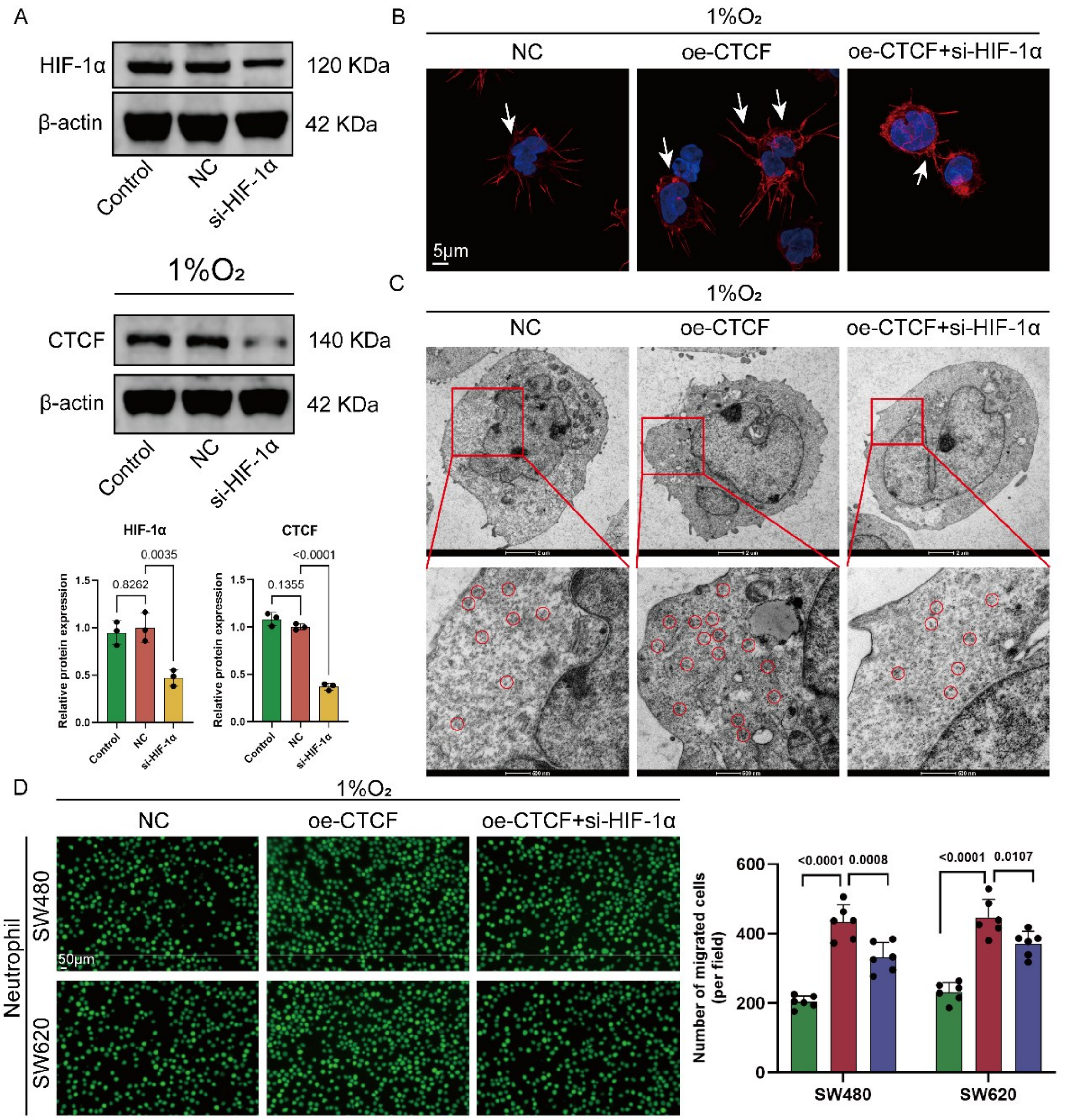

Figure S9. HIF-1α regulates CTCF expression and function in hypoxic HL60-derived TANs.

(A) Western blot analysis confirming siRNA-mediated knockdown of HIF-1α (Top row), showing reduced CTCF protein expression in hypoxic (1% O_2) TANs (Bottom row). Representative blot from three independent experiments is shown.

(B) Immunofluorescence staining of differentiated HL60 cells showing cytoskeletal

proteins (red) and nuclei (blue). Over-expression of CTCF promoted cytoskeletal remodeling, an effect that was markedly attenuated by HIF-1α knockdown. White arrows denote pseudopodia. Scale bar: 5 μm.

(C) Transmission electron microscopy of hypoxic differentiated HL60 cells demonstrated increased ribosome density in CTCF-over-expressing cells, reversed by HIF-1α silencing. Top row: Overview of cellular ultrastructure (scale bar: 2 μm). Bottom row: Magnified views showing ribosomes (circled in red, scale bar: 500 nm).

(D) Migration assays showed increased chemotactic response of hypoxic TANs toward CRC cells upon CTCF over-expression, which was significantly reduced by HIF-1α knockdown.

Data are presented as mean ± SD, statistical significance was determined by one-way ANOVA with Tukey's post hoc test. P values are indicated for each comparison.

Figure S10

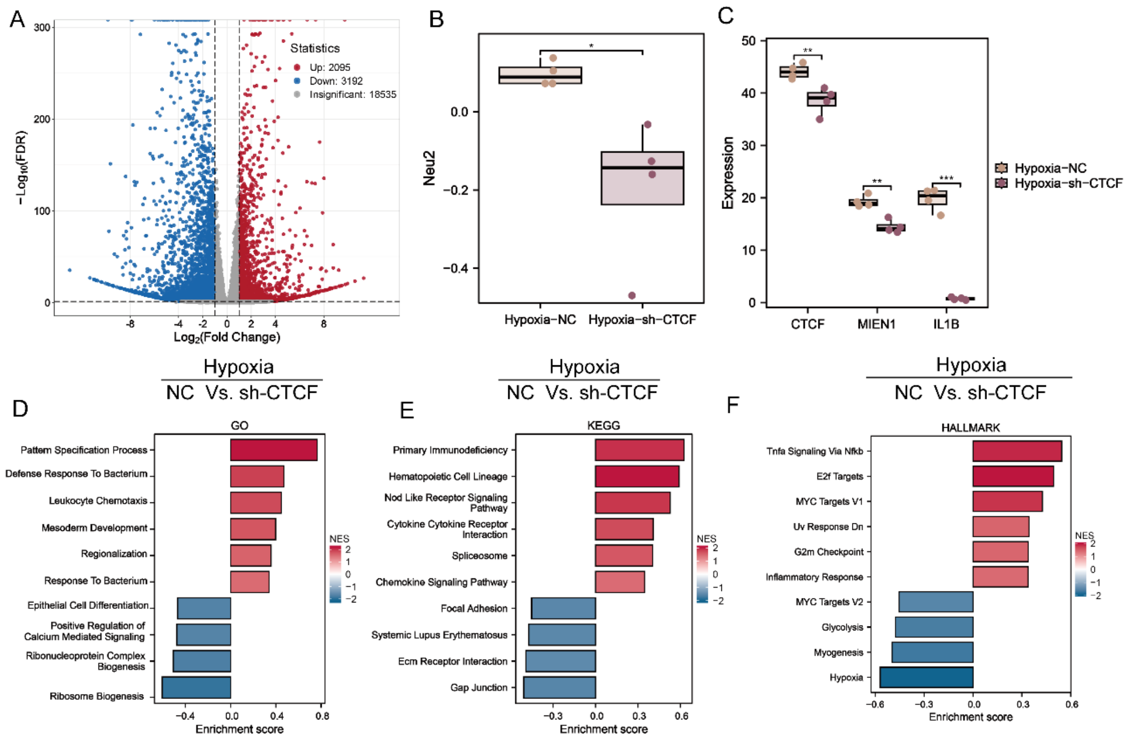

Figure S10. CTCF knockdown alters transcriptomic profiles and functional pathways in hypoxic HL60-derived TANs.

(A) Volcano plot showing differential gene expression between hypoxic CTCF-knockdown (sh-CTCF) and control (NC) TANs. Up-regulated genes (red) and down-regulated genes (blue) are indicated, with the number of significant genes highlighted.

(B) Box plot showing the overall expression level of the Neu2-associated gene signature in hypoxic TANs. CTCF knockdown (sh-CTCF) significantly reduced the collective expression of this gene set compared to the control (NC).

(C) Box plot of expression levels of CTCF, MIEN1, and IL1B in hypoxic HL60 (NC vs. sh-CTCF). CTCF knockdown significantly down-regulates these Neu2 markers.

(D–F) Functional enrichment analyses, including Gene Ontology (GO) (D), Kyoto Encyclopedia of Genes and Genomes (KEGG) pathways (E), and Hallmark gene sets

(F), demonstrate the biological processes and pathways affected by CTCF knockdown in TANs under hypoxic conditions.

Statistical significance was assessed using Student's t-test, with *P < 0.05, **P < 0.01, and ***P < 0.001 indicating significant differences

Figure S11

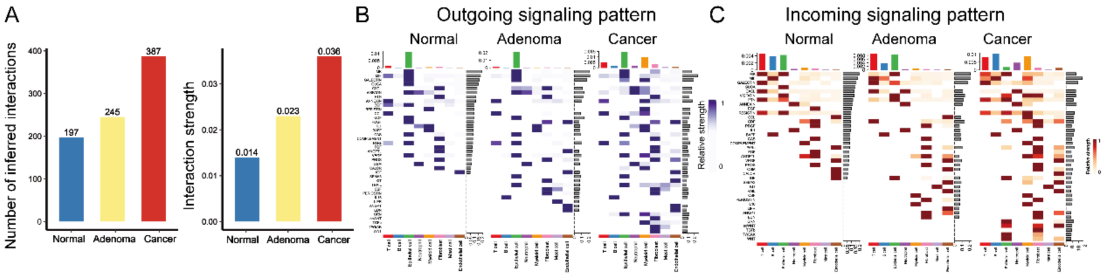

Figure S11. Progressive changes in cell-cell communication across normal, adenoma, and cancer tissues.

(A) Bar plots displaying the total number of inferred cell-cell interactions (left) and the average interaction strength (right) in normal (blue), adenoma (yellow), and cancer (red) tissues.

(B-C) Heatmaps illustrate outgoing (B) and incoming (C) signaling patterns across normal, adenoma, and cancer tissues. Rows represent signaling pathways, and columns correspond to cell type. Both patterns demonstrate a progressive increase in signaling complexity and strength from normal to adenoma to cancer.

Figure S12

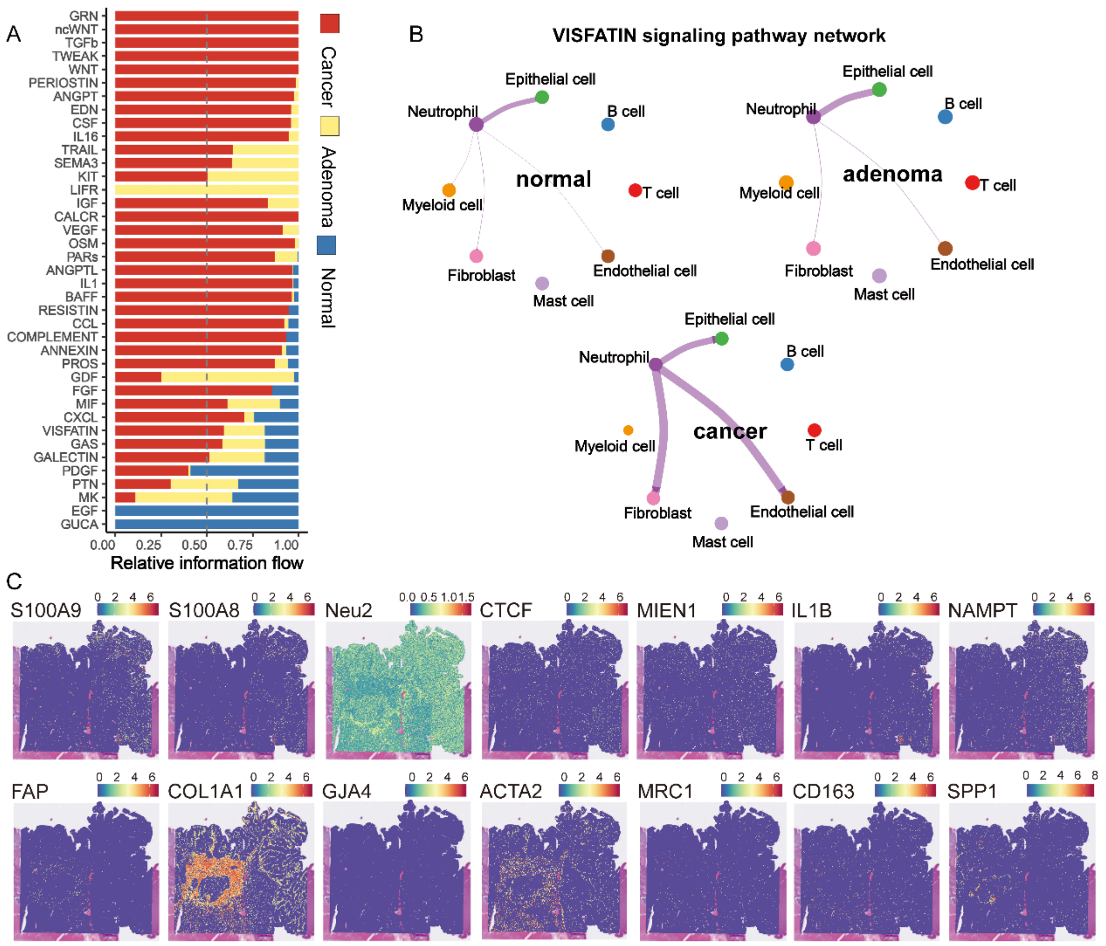

Figure S12. Signaling pathway analysis and spatial expression patterns in normal, adenoma, and cancer tissues.

(A) Bar plot showing the relative information flow of signaling pathways in normal (blue), adenoma (yellow), and cancer (red) tissues.

(B) Network diagrams of the VISFATIN signaling pathway in normal, adenoma, and cancer tissues. Node sizes represent cell types involved in the pathway (e.g., neutrophils, fibroblasts, T cells), and edge thickness indicates interaction strength.

(C) Spatial transcriptomics maps of a Visium HD sample (Data from the official 10x Genomics website) showing the distribution of Neu2 cells and the expression patterns

of key genes, including neutrophil markers (*S100A9*, *S100A8*), Neu2-associated markers (*CTCF*, *MIEN1*, *IL1B*), *NAMPT*, fibroblast markers (*FAP*, *COL1A1*, *GJA4*, *ACTA2*), and macrophage markers (*MRC1*, *CD163*, *SPP1*). The maps highlight the spatial localization of Neu2 cells themselves within the tumor microenvironment, alongside their associated gene signatures.

Figure S13

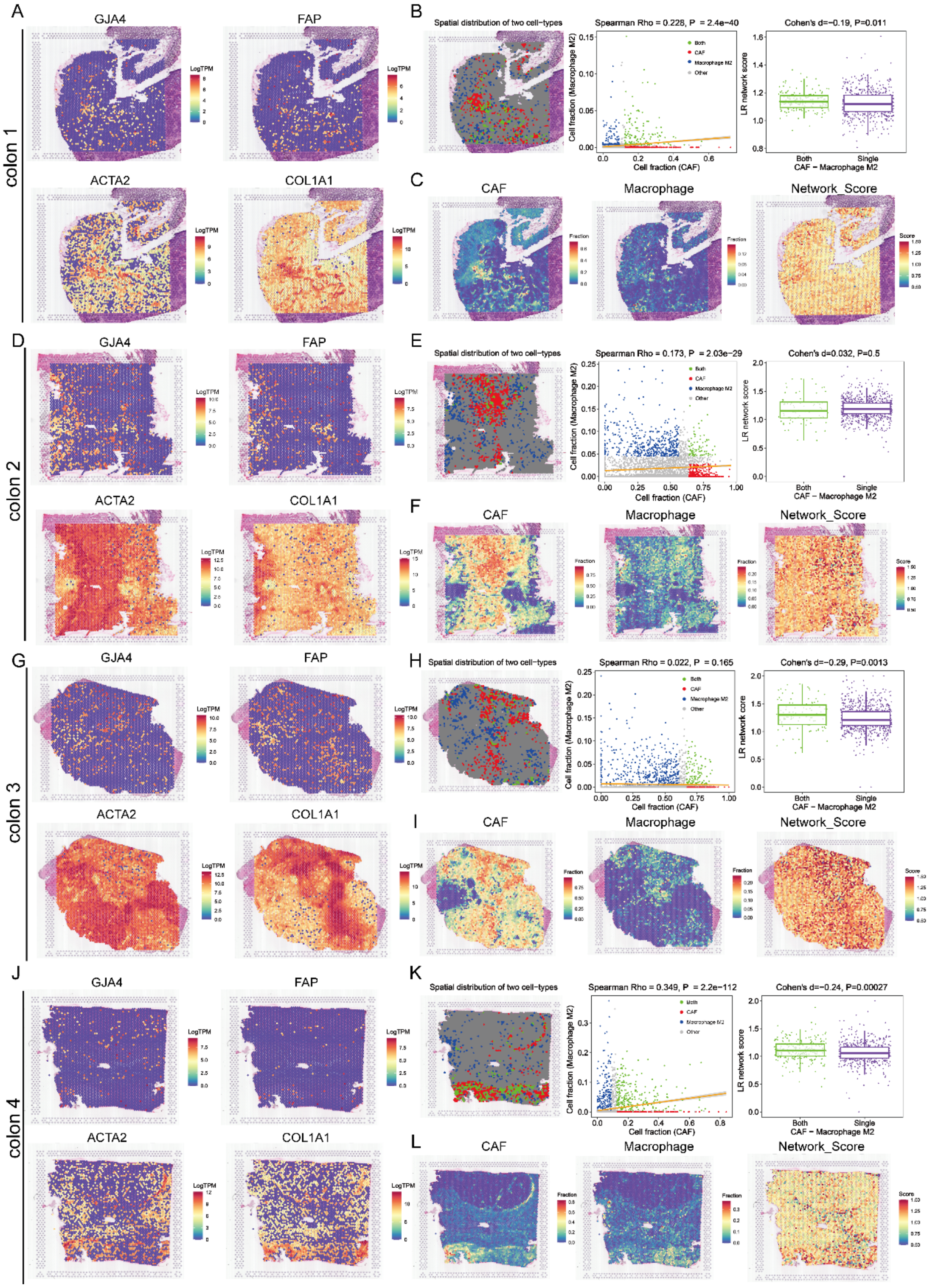

Figure S13. Spatial transcriptomics and ligand-receptor network interactions between CAFs and M2 macrophages across four colon tumor samples.

(A-L) Analysis of CAF markers, spatial distribution, and interactions between CAFs and M2 macrophages in four colon tumor samples (colon1: A-C, colon2: D-F, colon3: G-I, colon4: J-L).

CAF marker expression (A, D, G, J): Spatial transcriptomics maps showing the LogTPM expression levels of CAF markers (*GJA4*, *FAP*, *ACTA2*, *COL1A1*).

Spatial co-distribution and correlation (B, E, H, K): Maps illustrating the co-distribution of CAFs (red) and M2 macrophages (blue), highlighting overlapping regions in the tumor microenvironment. Scatter plots show a significant positive correlation between CAF and M2 macrophage fractions across all samples. Box plot comparing the ligand-receptor (LR) network score between areas with both CAFs and M2 macrophages ("Both") versus areas dominated by a single cell type ("Single").

LR network scores and spatial distribution (C, F, I, L): Spatial maps of CAF fractions, M2 macrophage fractions, and LR network scores. High LR network scores align with regions of CAF and M2 macrophage co-enrichment, emphasizing their synergistic role in enhancing cell-cell communication and remodeling the tumor microenvironment.

Figure S14

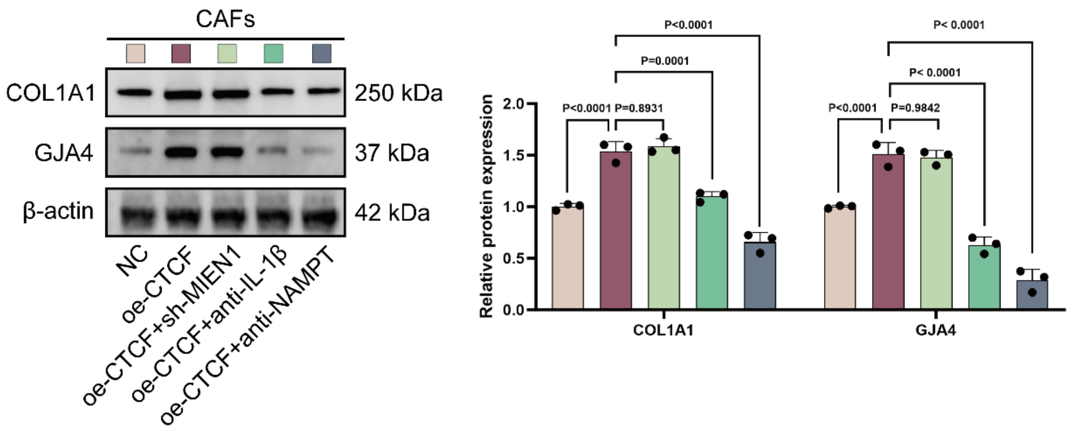

Figure S14. Western blot analysis showing COL1A1 and GJA4 expression in fibroblasts co-cultured with differentiated HL60 cells under different treatments: NC (control), oe-CTCF, oe-CTCF + sh-MIEN1, oe-CTCF + anti-IL-1β, and oe-CTCF + anti-NAMPT. β-actin serves as a loading control. Relative protein expression levels are quantified in the bar graph, with statistical significance indicated (P-values shown). Data are presented as mean ± SD. Statistical comparisons were performed using one-way ANOVA followed by Tukey's post-hoc test. P-values < 0.05 were considered statistically significant.

Figure S15

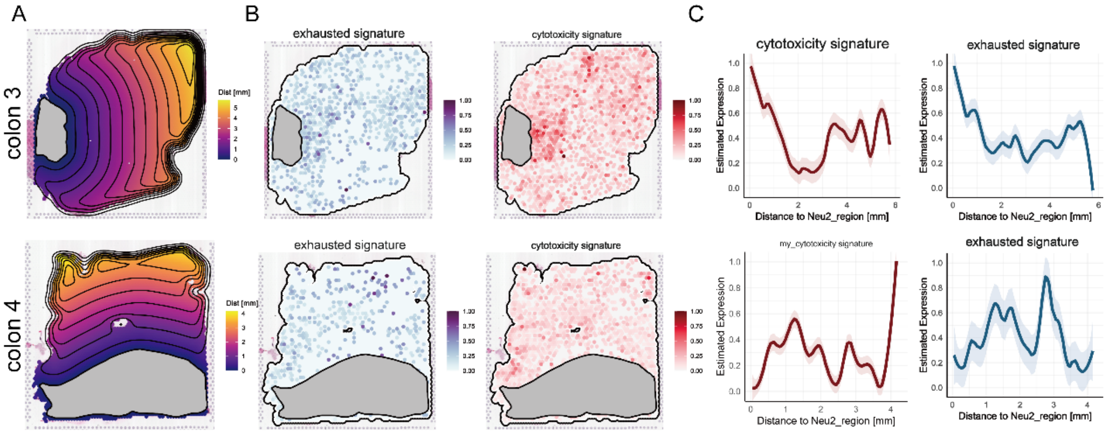

Figure S15. Spatial distribution and distance-based analysis of cytotoxic and exhausted immune signatures in colon tumors.

(A) Spatial maps of distance from Neu2-enriched regions in colon tumors (Colon3 and Colon4). Contour lines indicate the distance (in mm) from Neu2-dense regions, with closer areas shown in darker shades.

(B) Spatial transcriptomics maps displaying the distribution of cytotoxic (right, red gradient) and exhausted (left, blue gradient) immune signatures in Colon3 and Colon4.

(C) Line plots showing the estimated expression of cytotoxic (left) and exhausted (right) immune signatures as a function of distance from Neu2-enriched regions.

Figure S16

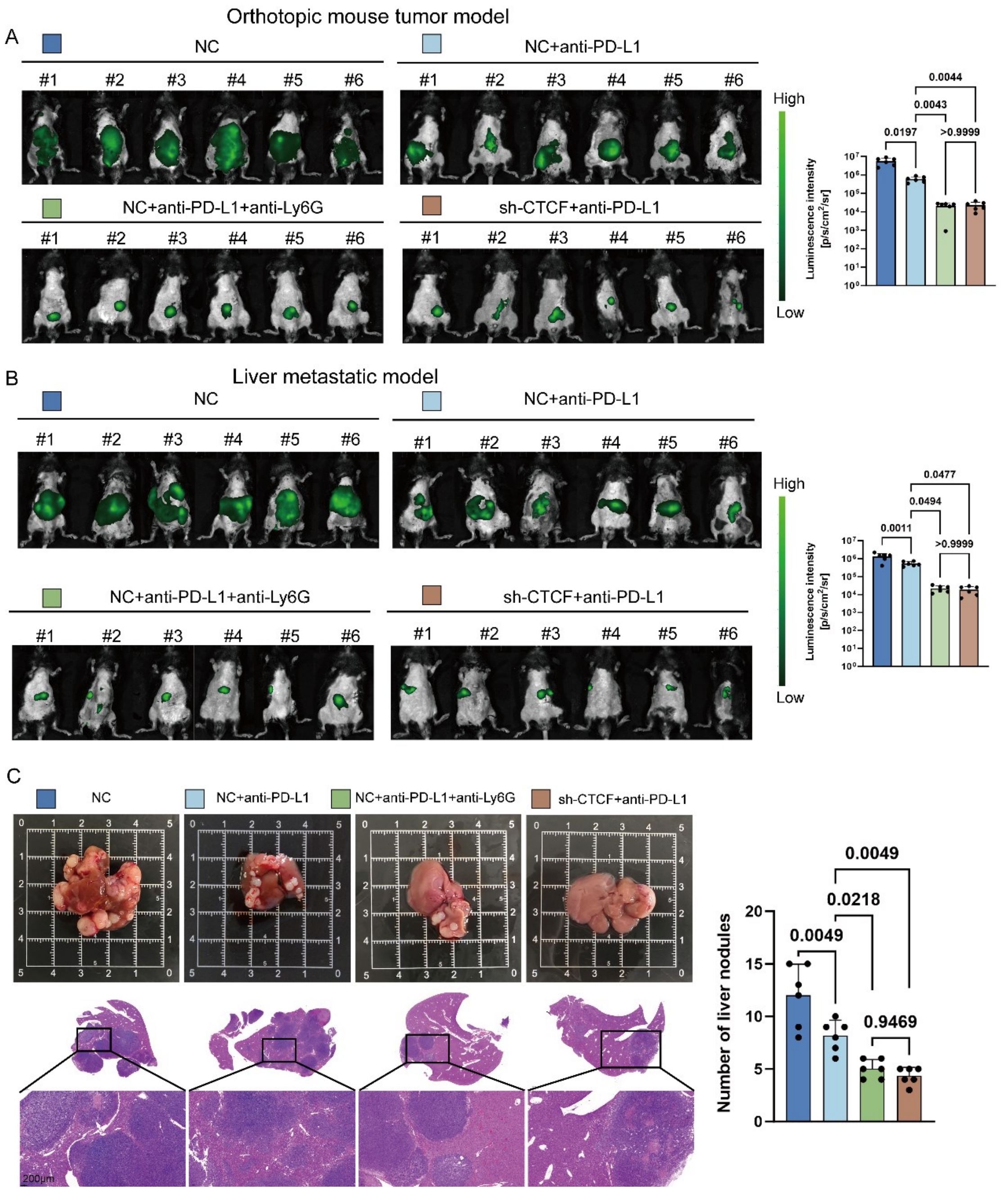

Figure S16. CTCF Silencing and neutrophil depletion show comparable enhancement of anti-PD-L1 therapy in CRC tumor and metastasis models.

(A-B) Orthotopic mouse tumor model (A) and liver metastatic model (B) showing

bioluminescence imaging of tumor growth in different treatment groups. (C) Tumor and liver metastasis morphology and histological analysis. The number of liver nodules is significantly reduced in the sh-CTCF+anti-PD-L1 and NC+anti-PD-L1+anti-Ly6G groups. Representative HE staining of liver tissue sections shows the reduction in metastasis in these treatment groups. Scale bar: 200 μm.

Data are presented as mean ± SD, statistical significance was assessed using one-way ANOVA followed by Tukey's post hoc test. P values are indicated for each comparison.

References

1. Collins SJ, Ruscetti FW, Gallagher RE, Gallo RC. Terminal differentiation of human promyelocytic leukemia cells induced by dimethyl sulfoxide and other polar compounds. Proc Natl Acad Sci U S A. 1978; 75: 2458-62.
2. Wang GG, Calvo KR, Pasillas MP, Sykes DB, Häcker H, Kamps MP. Quantitative production of macrophages or neutrophils ex vivo using conditional Hoxb8. Nature Methods. 2006; 3: 287-93.
3. Zou Z, Hu X, Luo T, Ming Z, Chen X, Xia L, et al. Naturally-occurring spinosyn A and its derivatives function as argininosuccinate synthase activator and tumor inhibitor. Nat Commun. 2021; 12: 2263.
4. Sun M, Li H, Hou Y, Huang N, Xia X, Zhu H, et al. Multifunctional tendon-mimetic hydrogels. Sci Adv. 2023; 9: eade6973.
5. Dolznig H, Rupp C, Puri C, Haslinger C, Schweifer N, Wieser E, et al. Modeling colon adenocarcinomas in vitro a 3D co-culture system induces cancer-relevant pathways upon tumor cell and stromal fibroblast interaction. Am J Pathol. 2011; 179: 487-501.
6. Tarragó MG, Chini CCS, Kanamori KS, Warner GM, Caride A, de Oliveira GC, et al. A Potent and Specific CD38 Inhibitor Ameliorates Age-Related Metabolic Dysfunction by Reversing Tissue NAD(+) Decline. Cell Metab. 2018; 27: 1081-95.e10.
7. Wang L, Li G, Cao L, Dong Y, Wang Y, Wang S, et al. An ultrasound-driven immune-boosting molecular machine for systemic tumor suppression. Sci Adv. 2021; 7: eabj4796.
8. Kang J, Zheng Z, Li X, Huang T, Rong D, Liu X, et al. Midazolam exhibits antitumour and enhances the efficiency of Anti-PD-1 immunotherapy in hepatocellular carcinoma. Cancer Cell Int. 2022; 22: 312.
